# Cardiopulmonary hospitalization risks from wildfire-specific and non-wildfire PM_2.5_ in 20 US states

**DOI:** 10.1038/s41467-026-76974-7

**Published:** 2026-09-17

**Authors:** Min Zhang, Edgar Castro, Minghao Qiu, Mahdieh Danesh Yazdi, Boyuan Li, Rosalind J. Wright, Joel D. Schwartz, Robert O. Wright, Yaguang Wei

**Affiliations:** 1 Department of Environmental Medicine, Icahn School of Medicine at Mount Sinai, New York, NY USA; 2 Department of Environmental Health, Harvard T.H. Chan School of Public Health, Boston, MA USA; 3 School of Marine and Atmospheric Sciences, Stony Brook University, Stony Brook, NY USA; 4 Program in Public Health, Department of Family, Population, and Preventive Medicine, Stony Brook University, Stony Brook, NY USA; 5 Department of Chemical and Biomolecular Engineering, Tandon School of Engineering, New York University, Brooklyn, NY USA; 6 Department of Public Health, Icahn School of Medicine at Mount Sinai, New York, NY USA; 7 Department of Epidemiology, Harvard T.H. Chan School of Public Health, Boston, MA USA

**Keywords:** Cardiovascular diseases, Environmental impact, Respiratory tract diseases

## Abstract

Increasing wildfire activity in the US has made wildfire-specific fine particulate matter (PM_2.5_) an important and growing source of air pollution, yet its long-term health impacts and relative toxicity compared with non-wildfire PM_2.5_ remain unclear. Using a self-controlled design, we examine associations between 2-year average wildfire-specific and non-wildfire PM_2.5_ and cardiopulmonary hospitalization risks across 20 US states during 2006–2019. Per 1 µg/m^3^ increase, wildfire-specific PM_2.5_ is associated with significantly higher hospitalization risks for all cardiopulmonary diseases, with relative risks ranging from 10% for heart failure to 16% for asthma. In contrast, a 1-µg/m^3^ increase in non-wildfire PM_2.5_ is associated with smaller risk elevations, ranging from 4.7% for chronic obstructive pulmonary disease to 8.5% for hypertension. Stronger associations for both wildfire-specific and non-wildfire PM_2.5_ are observed among minorities, metropolitan residents, those with fewer years of education, and more deprived communities. Overall, at an equivalent concentration increase, long-term exposure to wildfire-specific PM_2.5_ poses greater cardiopulmonary hospitalization risks than non-wildfire PM_2.5_, underscoring wildfire smoke as a growing public health threat that requires targeted mitigation alongside conventional air quality control strategies.

## Abstract (web summary)

Authors found wildfire-specific PM_2.5_ showed greater toxicity per unit exposure than non-wildfire PM_2.5_ for cardiopulmonary hospitalizations. As wildfire smoke becomes a major and growing source of air pollution, stronger mitigation strategies are needed.

## Introduction

Fine particulate matter (PM_2.5_), a major air pollutant, has been identified as the leading environmental risk factor for cardiopulmonary diseases^1–3^. PM_2.5_ can be drawn deep into the lungs and may pass into the bloodstream, damaging vital organs such as the lungs and heart^4^. Ambient PM_2.5_ originates from various sources, including traffic, industrial activities, and agriculture. More recently, wildfires are emerging as a significant source of PM_2.5_ due to their increasing frequency and intensity driven by the changing climate^5–7^, and the expansion of human activity into fire regions^8^. Globally, the extent of fire-prone areas is expected to expand by 29% towards the late 21st century^9^. In many parts of the US, wildfire smoke has accounted for over half of the annual PM_2.5_ concentration in extreme smoke years, and has led to reversal of the declining trend in ambient PM_2.5_ over the past decades due to the Clean Air Act^10^. As a major toxic component of wildfire smoke, PM_2.5_ can be transported over hundreds of miles, posing a threat to public health far beyond the active fire region^11–13^.

Wildfire-specific PM_2.5_ contains higher levels of submicron particles, carbonaceous material (i.e., elemental and organic carbon), and polar organic compounds compared to non-wildfire PM_2.5_^14–17^. Toxicological studies have shown that, at equivalent doses, wildfire-specific PM_2.5_ induces greater cardiopulmonary toxicity than PM_2.5_ from other sources^18,19^. However, large-scale evidence comparing the relative toxicity of wildfire-specific and non-wildfire PM_2.5_ is limited^20,21^. In addition, most existing studies on health effects of wildfire-specific PM_2.5_ have focused on short-term impacts^13,22,23^, which overlooked the potential accumulation of particles in the body and their sustained effects over time. Addressing these gaps is critical to fully quantify health impacts of wildfires and support the development of long-term strategies to mitigate these impacts in response to the increasing wildfire activity.

In this study, we utilized hospitalization data for the residents of 20 US states between 2006 and 2019 to explore the long-term effects of exposure to wildfire-specific and non-wildfire PM_2.5_ on hospitalization risks of all major types of cardiovascular and pulmonary diseases. We linked patients’ residential ZIP codes with two spatiotemporal models of ambient wildfire-specific and non-wildfire PM_2.5_ levels to estimate their exposure levels. We used a self-controlled study design that inherently adjusts for time-invariant unmeasured confounders, such as genetics, while also controlling for measured covariates.

## Results

### Hospitalized patient characteristics

A total of 57,264,966 hospitalizations for cardiovascular diseases and 32,691,394 for pulmonary diseases were included in this study (Supplementary Table 1). The largest proportion of hospitalization for cardiovascular diseases and pulmonary diseases were hypertension (27.5%) and other respiratory diseases (42.4%), respectively. Patients hospitalized for acute respiratory diseases and asthma were younger than those for other diseases. The majority of patients were White and residents of metropolitan areas. Comorbidity with obesity was most prevalent among patients hospitalized for asthma (12.0%), while diabetes was most prevalent among those hospitalized for heart failure (32.8%).

### Exposure levels

**Fig. 1:**
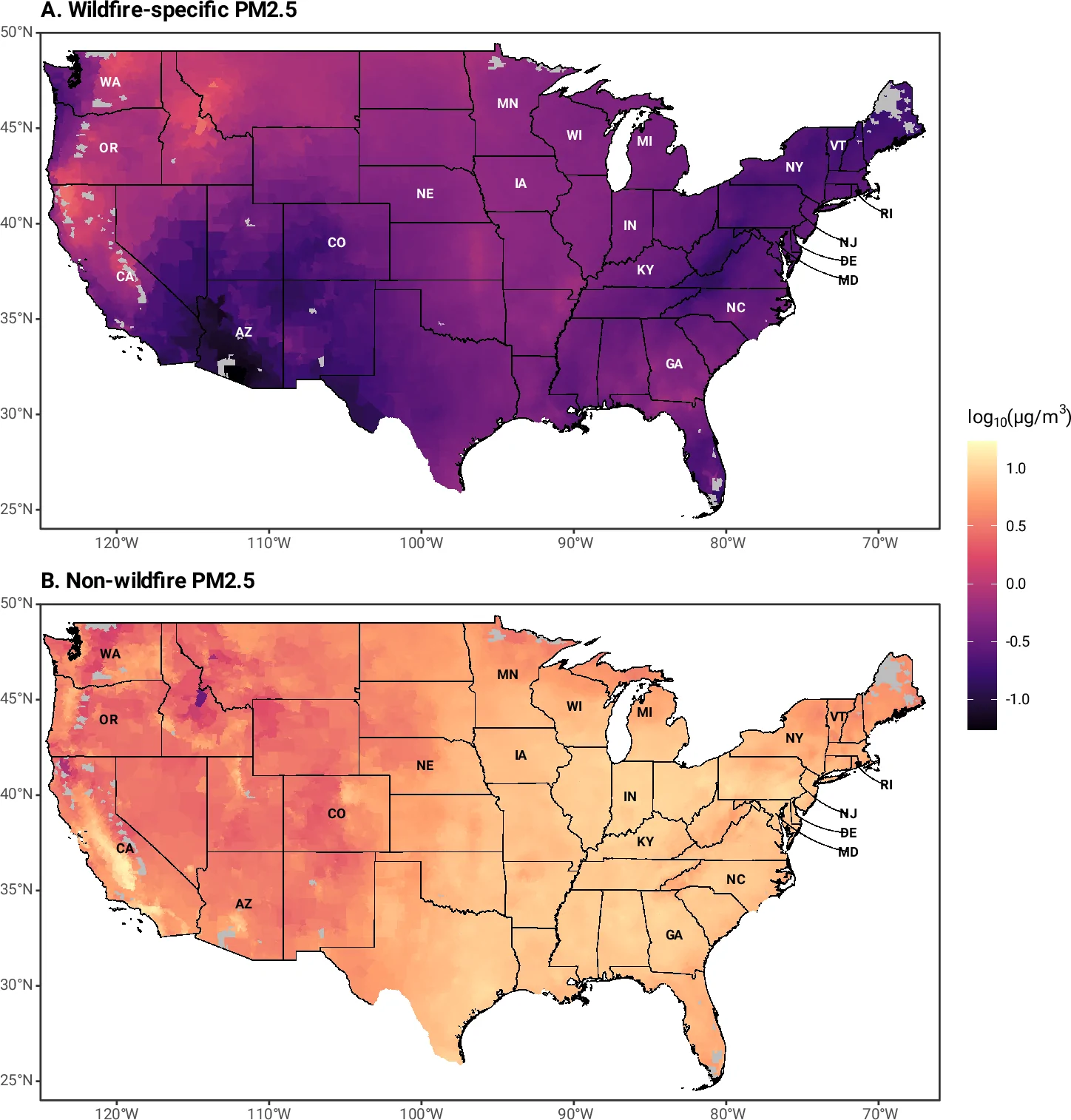
Average concentrations of PM_2.5_ (μg/m^3^) in the contiguous United States from 2006 to 2019. **A** wildfire-specific PM_2.5_; **B** non-wildfire PM_2.5_. States shown with abbreviations in the inset are included in the study. Values are plotted on a log_10_ scale, with yellow indicating higher concentrations, dark purple lower concentrations, and light yellow corresponding to log_10_ = 0 (i.e., 1 μg/m^3^). Source data are provided as a Source Data file. Abbreviations: PM_2.5_, fine particulate matter.

Among the 20 US states studied, California recorded the highest concentrations of both wildfire-specific PM_2.5_ and non-wildfire PM_2.5_ (Fig. 1). During the study period, the annual mean wildfire-specific PM_2.5_ at ZIP code level was 0.29 (range: 0.00–11.15) μg/m^3^, and 7.78 (range: 0.005–22.78) μg/m^3^ for non-wildfire PM_2.5_ (Supplementary Table 1). The 2-year average non-wildfire PM_2.5_ concentrations were approximately 20–27 times higher than that of wildfire-specific PM_2.5_ (Supplementary Table 2).

### Wildfire versus non-wildfire PM_2.5_ and hospitalization risks

**Fig. 2:**
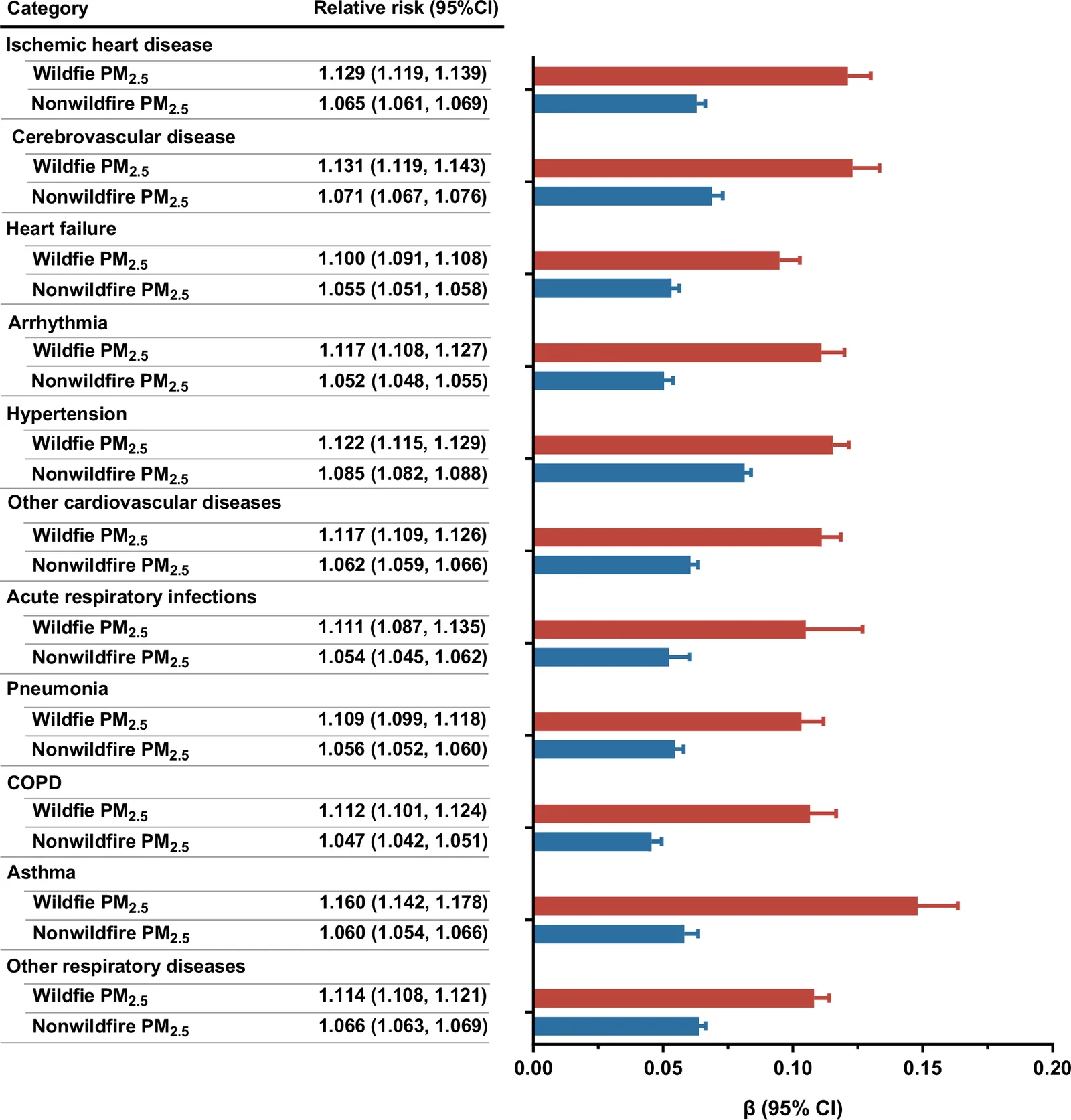
The hospitalization risks of cardiopulmonary diseases associated with a 1-μg/m^3^ increase in 2-year average of wildfire-specific PM_2.5_ and non-wildfire PM_2.5_ concentrations. The right panel shows regression coefficients (*β*), with error bars representing 95% confidence intervals. Red indicates wildfire-specific PM_2.5_, and blue indicates non-wildfire PM_2.5_. Relative risks (RR = exp(β)) and Bonferroni-corrected 95% confidence intervals were estimated from conditional logistic regression models adjusting for calendar year, percentage of White, percentage of Black, percentage of the population who graduated high school, median household income, Townsend Deprivation Index, mean temperature of summer and winter, and standard deviation of summer and winter temperature. All statistical tests were two-sided. Source data are provided as a Source Data file. Abbreviations: PM_2.5_ fine particulate matter, COPD chronic obstructive pulmonary disease.

Compared to non-wildfire PM_2.5_, a 1-μg/m^3^ increase in wildfire-specific PM_2.5_ was associated with greater risks of hospitalizations for all the examined cardiopulmonary diseases (Fig. 2). Each 1-μg/m^3^ increase in 2-year average wildfire-specific PM_2.5_ concentrations was statistically significantly associated with increased hospitalization risks for cerebrovascular disease (relative risk [RR] = 1.131; 95% confidence intervals [CI]: 1.119–1.143), ischemic heart disease (RR = 1.129; 95% CI:1.119–1.139), hypertension (RR = 1.122; 95% CI: 1.115–1.129), arrhythmia (RR = 1.117; 95% CI: 1.108–1.127), other cardiovascular diseases (RR = 1.117; 95% CI:1.109–1.126), and heart failure (RR = 1.100; 95% CI: 1.091–1.108). For pulmonary diseases, wildfire-specific PM_2.5_ had the greatest impact on asthma, with an RR of 1.160 (95% CI: 1.142–1.178); the smallest but still significant association was observed for pneumonia, with an RR of 1.109 (95% CI: 1.099–1.118). Positive associations were also observed between non-wildfire PM_2.5_ and hospitalization risks of all cardiopulmonary diseases (Fig. 2). A 1-μg/m^3^ increase in 2-year average non-wildfire PM_2.5_ concentrations was statistically significantly associated with increased risks of cardiovascular hospitalization: 1.085 (95% CI: 1.082–1.088) for hypertension, 1.071% (95% CI: 1.067–1.076) for cerebrovascular disease, 1.065 (95% CI: 1.061–1.069) for ischemic heart disease, 1.062 (95% CI: 1.059–1.066) for other cardiovascular diseases, 1.055 (95% CI: 1.051–1.058) for heart failure, and 1.052 (95% CI: 1.048–1.055) for arrhythmia. Among all pulmonary diseases, the association was the largest for other respiratory diseases (RR = 1.066; 95% CI: 1.063–1.069), followed by asthma (RR = 1.060; 95% CI: 1.054–1.066), and pneumonia (RR = 1.0576; 95% CI: 1.052–1.060). However, given the substantially lower concentrations of wildfire-specific PM_2.5_, the cardiopulmonary hospitalization risks increase per standard deviation (SD), which may better capture the relative magnitude of exposure changes in reality, was greater for non-wildfire PM_2.5_ (Supplementary Table 3).

### Sensitivity analysis

Overall, the associations between long-term exposure to wildfire-specific PM_2.5_ and non-wildfire PM_2.5_ and risks of cardiopulmonary hospitalizations remained robust after changing covariates adjusted in the model (Supplementary Tables 4, 5). The effect estimates remained largely unchanged when hospitalizations were identified based solely on the principal diagnosis code at discharge (Supplementary Table 6) or when negative values of non-wildfire PM_2.5_ were modeled as zero instead of being excluded (Supplementary Table 7). When alternative exposure windows were considered using 3- or 4-year moving averages, the association between wildfire-specific PM_2.5_ and cardiopulmonary hospitalizations became stronger with longer exposure duration, whereas the association with non-wildfire PM_2.5_ remained relatively stable (Supplementary Table 8). Furthermore, results were consistent under alternative control-year specifications, indicating that the self-controlled design was not materially sensitive to the selection of control years (Supplementary Table 9). In stratified analyses by US state, associations appeared stronger in states with high PM_2.5_ levels, particularly in California (Supplementary Table 10).

### Stratification analysis

**Fig. 3:**
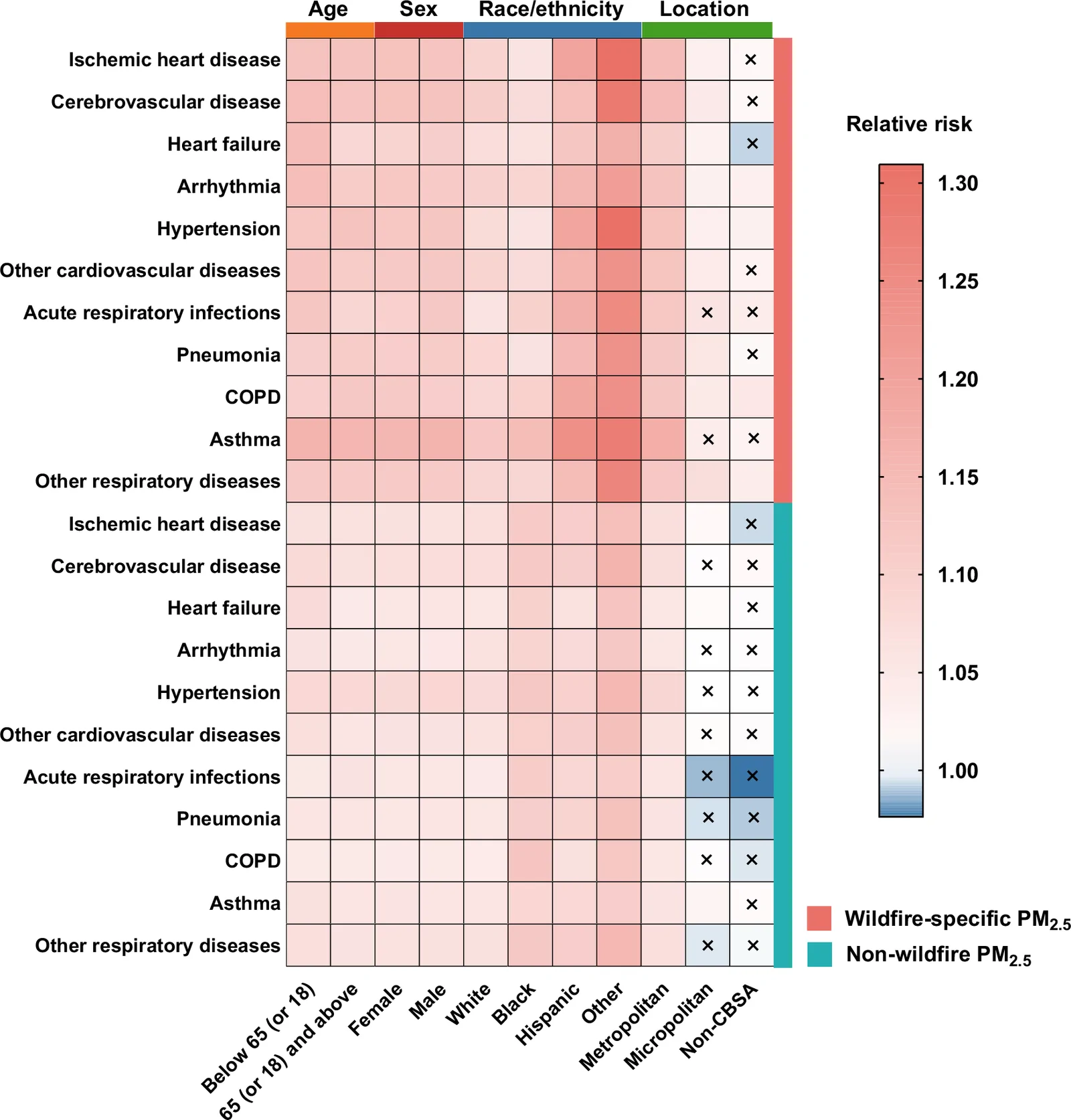
Heatmap of relative risks (RRs) from subgroup analyses by age, sex, race/ethnicity, and patient location. In the heatmap, cell colors represent point estimates of RRs, with white indicating a value of 1, red indicating higher RRs (>1), and blue indicating lower RRs (<1). RRs were estimated from conditional logistic regression models. Top bars of the heatmap indicate subgroup categories: orange for age, red for sex, blue for race/ethnicity, and green for patient location. The vertical bars on the right indicate exposure type, with light red representing wildfire-specific PM_2.5_ and light blue representing non-wildfire PM_2.5_. Tiles marked with a cross symbol (×) indicate that the corresponding 95% confidence interval overlaps 1 and therefore should not be interpreted as statistically significant changes in relative risk. Tiles without a cross (×) indicate that the corresponding 95% confidence interval does not overlap 1. Age was stratified at 18 years for acute respiratory infections and asthma, and at 65 years for all other disease outcomes. All numerical results, as well as the results of between-group comparisons, can be found in Supplementary Tables 11–14. All statistical tests were two-sided. Source data are provided as a Source Data file. Abbreviations: COPD chronic obstructive pulmonary disease, non-CBSA non-Core Based Statistical Areas.

**Fig. 4:**
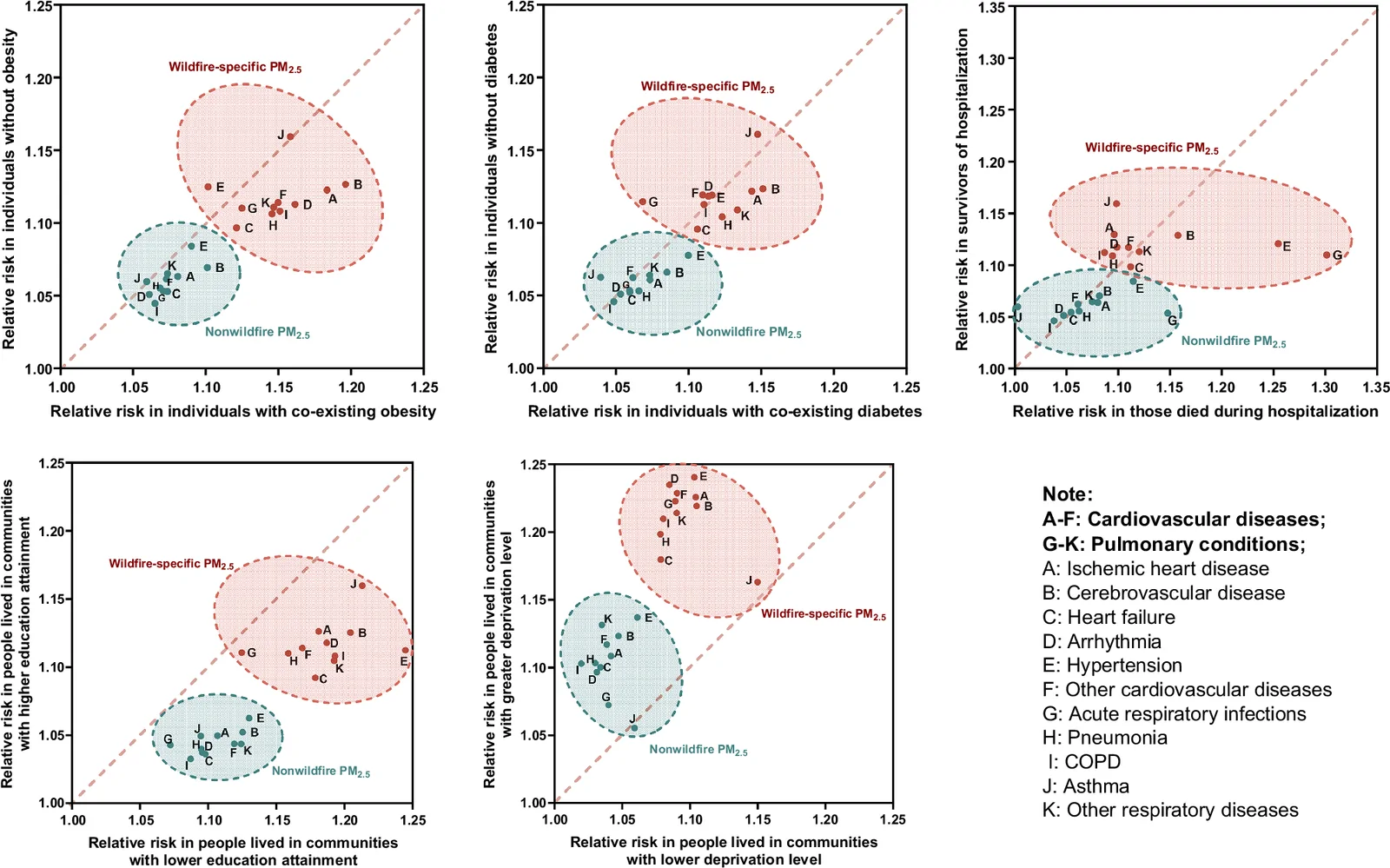
Scatter plot of relative risks (RRs) from subgroup analyses based on comorbidities, disease severity, and community-level factors. RRs were estimated from conditional logistic regression models. Red indicates wildfire-specific PM_2.5_, and blue indicates non-wildfire PM_2.5_. The greater the distance of a point from the diagonal line, the higher the relative risk it represents within a specific subgroup. The shaded areas are used solely for visual differentiation between wildfire-specific PM_2.5_ and non-wildfire PM_2.5_ and do not indicate statistical significance. All relative risks shown have 95% confidence intervals entirely above 1. The percentage of the population who graduated high school was divided into two groups based on the lower quartile (25th percentile): lower and higher educational attainment. Townsend Deprivation Index was categorized into two groups based on the upper quartile (75th percentile): lower and higher deprivation levels. Detailed numerical results, as well as the results of between-group comparisons, are provided in Supplementary Tables 15–19. All statistical tests were two-sided. Source data are provided as a Source Data file. Abbreviations: PM_2.5_ fine particulate matter, COPD chronic obstructive pulmonary disease.

Hispanic individuals and those from other racial/ethnic groups experienced greater cardiopulmonary hospitalization risks (Fig. 3). Stronger particle associated risk was observed among people who lived in metropolitan areas (Fig. 3), communities with lower education attainment, or communities with greater deprivation level (Fig. 4). Moreover, the presence of co-existing obesity or diabetes was generally associated with higher PM_2.5_-related hospitalization risks for most types of cardiopulmonary diseases (Fig. 4). There were few differences in PM_2.5_-related cardiopulmonary hospitalization risks across subpopulations defined by age, sex, and disease severity (Figs. 3–4; Supplementary Tables 11–19).

## Discussion

In this study across 20 US states, a 2-year average exposure to both wildfire-specific PM_2.5_ and non-wildfire PM_2.5_ was associated with increased hospitalization risks for all major types of cardiopulmonary diseases. Although most wildfires are abrupt events, our findings suggest that their health effects are persistent, and there is a need for long-term wildfire management strategies and protective measures to reduce exposure to smoke. Importantly, we found that wildfire-specific PM_2.5_ posed greater hospitalization risks per unit exposure, suggesting greater effort should be placed on wildfire management in addition to relying solely on traditional air quality control strategies. Wildfire has been increasing in frequency, size, and intensity across the US over recent decades, and has become the fastest-growing source of ambient air pollution. Currently, these strategies largely exempt wildfire smoke from determining attainment of the National Ambient Air Quality Standards (NAAQS)^24^. Moreover, traditional wildfire management approaches, such as controlled burns, have primarily focused on protecting property rather than minimizing population exposure to smoke, an aspect that should be more fully integrated in future efforts. Greater susceptibility to both wildfire-specific PM_2.5_ and non-wildfire PM_2.5_ was found among minorities, those with co-existing obesity or diabetes, individuals in metropolitan areas, and communities with lower education attainment and greater deprivation level.

We used a self-controlled design to evaluate the long-term effects of exposure. Unlike the conventional case-crossover design, which is primarily used to assess short-term effects^25^, our design selects control years both before and after the case year to carry out the assessment over a longer time horizon. We adjusted for a wide range of slow-varying confounders that are likely stable over short-term exposures but may change over longer exposure windows, such as calendar year, seasonal temperature, and neighborhood-level statistics. This approach preserves the strengths of the case-crossover design, in particular its ability to control for time-invariant confounders, such as genetic susceptibility, smoking history, and educational attainment, while expanding its applicability to study long-term effects. This is especially relevant because older adults accounted for the majority of hospitalizations in our study.

Our findings are consistent with the emerging evidence on the associations between exposure to wildfire-specific PM_2.5_ and increased risks of pulmonary diseases, especially asthma^21,26,27^. We observed that a 1-μg/m^3^ increase in wildfire-specific PM_2.5_ concentrations was associated with a 10.9%–16.0% increase in pulmonary hospitalization risks, which is generally higher than estimates reported in previous studies (0.25–2.3%)^21,28^. The larger magnitudes of associations might be due to the longer exposure window than previous studies. Our earlier work showed that chronic exposure to ambient air toxicants posed substantially larger risks than acute exposures over a few days, potentially due to the accumulation of toxicants in the body and their sustained adverse effects on various organs over time^29^.

For the effects of wildfire emissions on cardiovascular diseases, existing evidence remains less conclusive^30–33^. Some studies have reported positive associations^28,34^, whereas others have found little evidence of increased risk during wildfire episodes^35–39^ or in association with short-term exposure to wildfire-specific PM_2.5_^26,27,40–42^. Previous studies used short exposure windows (typically within 1 week), which may fail to capture the long-term health impacts of smoke on cardiovascular outcomes. One study suggested that the increase in emergency department visits for cardiovascular diseases associated with high levels of wildfire-related PM_2.5_ exposure appeared on lag day 10, considerably later than the effect observed for pulmonary diseases, which began on lag day 0^43^. The relatively small effect of short-term exposure may reduce statistical power, which increases the likelihood of nonsignificant or even reversing results. Differences in exposure assessment, study population, and study design may also explain varied results across studies.

Our research demonstrated that each 1-μg/m^3^ increase in wildfire-specific PM_2.5_ was associated with a greater risk of cardiopulmonary hospitalizations compared to non-wildfire PM_2.5_. Similar results were reported in a multi-city study across eight countries and territories^21^ and in another US study from Southern California^20^, which suggested that wildfire-specific PM_2.5_ led to 3 to 10 times higher risk of pulmonary hospital admissions compared to PM_2.5_ from non-wildfire sources. Aligned with these findings, a mouse model study showed that exposure to wildfire-derived PM at doses equivalent to ambient PM induced stronger pulmonary inflammation and histological lung damage^19^. In mice exposed to PM from smoldering peat fire, lung inflammation associated with endotoxins and reactive oxygen species, reduced cardiac function, and post-ischemia-associated myocardial infarction were observed^18^. Furthermore, aligned with Hao, et al.^44^, we observed that at longer exposure windows, the risk of cardiopulmonary hospitalization associated with wildfire-specific PM_2.5_ increased, whereas the risk associated with non-wildfire PM_2.5_ remained relatively stable. These findings suggested that wildfire-specific PM_2.5_ may exert stronger cumulative toxic effects and be more harmful per unit than non-wildfire PM_2.5_.This elevated risk associated with wildfire-specific PM_2.5_ may be attributed to its unique chemical composition, which tends to contain a higher proportion of submicron and ultrafine particles, as well as elevated concentrations of oxidative and pro-inflammatory substances, such as polycyclic aromatic hydrocarbons^14–17,23,45–47^. Because components of wildfire-specific PM_2.5_ are smaller in size, they may more easily penetrate the human body, which facilitate their adverse effects. Although the risk per SD increase was approximately three times higher for non-wildfire PM_2.5_ than for wildfire-specific PM_2.5_, the SD of non-wildfire PM_2.5_ was about six times larger. Importantly, under future climate change scenarios, wildfire activity in the US is expected to increase substantially, with annual wildfire-specific PM_2.5_ concentrations potentially reaching 10 µg/m^3^ in some regions^48^. Therefore, despite its currently lower concentrations, the public health impact of wildfire-specific PM_2.5_ should not be underestimated.

We found that vulnerable subgroups were similar for wildfire-specific and non-wildfire PM_2.5_, with greater susceptibility to both observed among minorities, residents of communities with lower education attainment, and communities with greater deprivation level can be attributed to structural racism and socioeconomic disparities. Compared to those identifying as white or persons with higher social class, both minorities and persons with lower social class face multiple inequities, including poorer healthcare access, higher pollution burden, fewer neighborhood resources, and fewer resources to mitigate the health impacts of PM_2.5_^49–51^. In addition, individuals residing in metropolitan areas (larger urban centers) were more susceptible than those in micropolitan areas or non-Core Based Statistical Areas [non-CBSA], which represent smaller urban clusters and rural areas, respectively. This may be explained by higher background other pollutants (e.g., nitrogen dioxide and ozone) in metropolitan areas^52,53^, which may interact with particles and further enhance oxidative stress and cardiopulmonary toxicity^54–56^. Additionally, higher levels of social stress in metropolitan areas may activate neuroendocrine stress responses and promote chronic systemic inflammation^57,58^, leading to immune dysregulation and increased susceptibility to cardiopulmonary disease^59,60^, particularly in the presence of environmental stressors such as particles^61^. Moreover, both wildfire-specific and non-wildfire PM_2.5_ were generally associated with stronger effects for most types of cardiopulmonary diseases among individuals with co-existing obesity or diabetes, highlighting the synergistic effect of PM_2.5_ exposure and cardiopulmonary risk factors^62–64^. Notably, individuals with obesity were generally more sensitive to PM_2.5_ exposure than those with diabetes. One possible explanation is that individuals with obesity often have reduced cardiopulmonary function, along with systemic chronic inflammation and elevated oxidative stress, all of which may exacerbate the burden of PM_2.5_ exposure^64^. Consistent with previous studies^3,65^, we observed generally stronger associations between PM_2.5_ exposure and cardiovascular hospitalization risk among patients under 65 years of age. This pattern may be partly explained by survivor bias, whereby individuals who are more susceptible to PM_2.5_-related cardiovascular effects may experience events or even mortality earlier in life, resulting in attenuated associations among older adults. Our results also support the finding that both wildfire-specific and non-wildfire PM_2.5_ were associated with deaths caused by hospitalization for cardiopulmonary diseases, although the difference in risk between patients who died during hospitalization and those who survived was minimal.

Our study has several strengths. First, we included a large sample covering residents of all ages from 20 US states, including those most heavily affected by wildfires, such as California, Oregon, and Washington. Second, we used modeled wildfire-specific PM_2.5_ data with full spatial and temporal coverage, allowing comprehensive exposure assessment across all study areas. Additional strength includes comprehensive coverage of all major cardiopulmonary conditions, inclusion of individuals of all ages, and the use of a novel self-controlled design that minimizes bias from unmeasured time-invariant and slowly varying confounders. Our study also has a few limitations. First, the spatial resolution and predictive performance of our wildfire-specific PM_2.5_ prediction model were suboptimal, which may introduce exposure measurement error that may widen confidence intervals^66^. Second, estimating non-wildfire PM_2.5_ by subtracting wildfire-specific PM_2.5_ from total PM_2.5_ may introduce non-differential measurement error due to differences in modeling approaches; however, sensitivity analyses treating negative values as zero yielded nearly identical effect sizes (Supplementary Table S7), indicating that our findings are robust to this error. Third, due to data availability, this study included only 20 states with varying observation periods, and the most recent data are nearly 6 years old, which may limit generalizability. Fourth, the State Inpatient Databases (SID) lacks unique patient identifiers, and therefore repeated hospitalizations by the same individuals could not be identified. We were unable to account for potential within-person correlation, which may have led to underestimated standard errors and overly narrow confidence intervals^67^. Fifth, residual confounding may remain due to unmeasured time-varying factors, including individual behaviors, indoor exposures, medication use, and co-pollutants (e.g., nitrogen dioxide and ozone), as well as differences between ambient PM_2.5_ concentrations and actual personal exposure. Furthermore, the use of 2-year average exposure windows creates overlap between cases and controls, likely attenuating exposure contrasts and biasing effect estimates toward the null rather than inflate associations. Finally, although we distinguished wildfire-specific and non-wildfire PM_2.5_, chemical composition may vary by fuel type^68^, which we could not assess; future studies incorporating source characterization and chemical speciation are needed to explore potential heterogeneity in health effects.

In conclusion, our results demonstrate that, at an equivalent concentration increase, long-term exposure to wildfire-specific PM_2.5_ poses a greater risk of cardiopulmonary hospitalization than non-wildfire PM_2.5_. Furthermore, both wildfire-specific and non-wildfire PM_2.5_ had stronger effects among minorities, residents of metropolitan areas, communities with lower education attainment, and greater deprivation level. Considering the reversal of air quality improvements driven by rapidly increasing wildfire activity and the greater toxicity of wildfire-specific PM_2.5_, greater efforts should be placed on wildfire management in addition to relying solely on traditional air quality control strategies.

## Methods

This study was reviewed and approved as exempt by the Institutional Review Board of the Harvard T.H. Chan School of Public Health (Protocol No. IRB20-1905) under 45 CFR 46.104(d)(4), because the study used de-identified administrative data.

### Study population

The hospitalization data of 20 US states (Arizona, California, Colorado, Delaware, Georgia, Iowa, Indiana, Kentucky, Maryland, Michigan, Minnesota, North Carolina, Nebraska, New Jersey, New York, Oregon, Rhode Island, Vermont, Washington, and Wisconsin) from 2006 to 2019 was obtained from the SID. The SID, a set of state-specific databases developed as part of the Healthcare Cost and Utilization Project (HCUP), contains inpatient discharge records from community hospitals within each participating state^69^. The SID represents over 95% of all inpatient discharges nationwide^70^. The availability of SID data varies by year and state, and detailed information is provided in the Supplementary Materials (Supplementary Table 20).

Individual-level elements, including hospital admission year, age, sex, residential zip code, race/ethnicity (White, Black, Hispanic, or other), patient location (metropolitan areas, micropolitan areas, or non-CBSA), diagnosis codes and died during hospitalization (yes vs. no), were extracted from the SID. The cardiopulmonary hospitalizations were identified using the first 3 diagnosis codes at discharge from the *International Classification of Diseases, Ninth Revision or Tenth Revision (ICD-9 or ICD-10)*. The specific ICD codes can be found in Supplementary Table 21. The cardiopulmonary diseases of interest included ischemic heart disease, cerebrovascular disease, heart failure, arrhythmia, all other cardiovascular diseases, acute respiratory infections, pneumonia, Chronic obstructive pulmonary disease (COPD), asthma, and other respiratory diseases. In addition, co-existing conditions, including obesity or diabetes, were also identified from all secondary diagnosis codes at discharge.

### Wildfire-specific PM_2.5_

We obtained daily, ground-level wildfire-specific PM_2.5_ concentrations at a 10 × 10 km^2^ spatial resolution for the contiguous US from 2006 to 2019 from an open source. These data were estimated using a machine learning model that incorporated ground measurements of PM_2.5_, satellite, and reanalysis data sets^71^. The model achieved reasonable predictive capability, yielding a spatial out-of-sample *R*^2^ of 0.67 and a within-location *R*^2^ of 0.65. The grid-level wildfire-specific PM_2.5_ concentrations were aggregated to ZIP Code Tabulation Areas (ZCTAs) using population- and intersection area-weighted averages based on TIGER/Line Shapefiles from the US Census Bureau. These estimates were mapped to zip codes and then linked to the subjects based on their residential address recorded in the SID. For each hospitalization, the exposure window of interest was defined as the 2-year averages of wildfire-specific PM_2.5_ concentrations, covering both the year of hospitalization and the year before. We selected this exposure window a priori to balance capturing longer-term exposure with minimizing potential exposure misclassification. Using only the hospitalization year could introduce misclassification, as individuals admitted early in the year would be assigned exposure averaged over the entire calendar year. Conversely, restricting exposure to the year prior to hospitalization would exclude the hospitalization year, which may have the strongest effect based on prior evidence^3^.

### Non-wildfire PM_2.5_

We subtracted the wildfire-specific PM_2.5_ predictions from the all-sourced PM_2.5_ concentrations to obtain the estimated non-wildfire PM_2.5_ concentrations. Those with negative values, which accounted for less than 0.01% of the data, were excluded from this study. We developed high resolution (1 km × 1 km) daily all-sourced PM_2.5_ estimates across the contiguous US during 2006–2019 from a spatio-temporal ensemble model^72,73^. In brief, a set of predictors from satellite observations, land-use regression, meteorological data, and chemical transport models was used as input for three machine learning algorithms to estimate PM_2.5_ concentrations. The outputs from these models were then integrated using a geographically weighted generalized additive model, which accounts for spatial heterogeneity. The final model demonstrated well performance, with an average cross-validation *R*^2^ of 0.82. These grid-level estimates were aggregated to the zip code level, and 2-year averages were assigned to each hospitalization.

### Covariates

We extracted zip code-level characteristics from databases generated by US Decennial Census and American Community Survey (ACS)^74–78^. Percentage of White and percentage of Black were obtained from the Decennial Census (2000, 2010, and 2020), with values for intermediate years estimated using linear interpolation. Percentage of the population aged ≥65 years who graduated high school, median household income, and Townsend Deprivation Index (TDI) were derived from the 2000 Decennial Census and the 2011 ACS, interpolated for intermediate years, and ACS estimates were used for 2012–2019. TDI, an indicator of overall deprivation, is calculated based on four key elements: unemployment, overcrowded households, non-ownership of a car, and non-ownership of a home. A higher score indicates a greater level of deprivation^79^. These variables were adjusted as potential confounders.

Previous studies have linked ambient temperature to cardiopulmonary outcomes^69,80,81^. Temperature also directly influences wildfire activity and, therefore, acts as a confounder. The daily maximum and minimum temperatures (°C) at 1-km^2^ grid cells from 2006 to 2019 were derived from Daymet V4, which was developed by the Oak Ridge National Laboratory^82^. Daily average temperatures were obtained by averaging the minimum and maximum values. Using daily average temperatures, we calculated the seasonal mean and standard deviation for summer (June, July, and August) and winter (December of the previous year, January, and February) in each year. These meteorological data were aggregated to the zip code level to align with the spatial resolution of hospitalization records.

### Statistical analysis

In our previous study^28^, we extended the traditional case-crossover design, a self-controlled design originally developed for studying short-term transient effects and widely used in environmental epidemiology^83–85^, to evaluate medium-term effects. The design compares exposure during a case period with exposure during matched control periods within the same individual using conditional logistic regression, thereby inherently controlling for time-invariant confounders. Although traditionally applied to short-term exposures, the same framework can be extended to multi-year average exposures provided that exposure contrasts exist between case and control periods.

In this study, we further extended this framework to evaluate long-term effects of 2-year average exposures to wildfire-specific and non-wildfire PM_2.5_ on cardiopulmonary hospitalization risks. Specifically, for each case year (year of hospitalization), the years before and after served as that hospitalization’s own controls. With this self-controlled design, unmeasured time-invariant and slowly varying confounders, such as genetic susceptibility, chronic coexisting conditions (e.g., diabetes), smoking status, and educational attainment, are inherently adjusted by design. The symmetric bidirectional selection of control years before and after a case year removed the unmeasured confounding from long-term temporal trends (e.g., gradual changes in population-level wildfire smoke level). Selecting one single year before and after the case minimized temporal variations in unmeasured confounders.

We constructed a dataset for each disease and fitted a conditional logistic regression to estimate the simultaneous effects of long-term wildfire-specific and non-wildfire PM_2.5_ on the hospitalization risk, adjusting for measured time-varying confounders, including neighborhood-level covariates, temperatures, and calendar year. Computational details for the fast implementation of conditional logistic regression are provided in Supplementary Methods. We reported estimated RRs of cardiopulmonary hospitalization associated with a 1 μg/m^3^ increase in 2-year average wildfire-specific and non-wildfire PM_2.5_ concentrations. To address the potential inflation of type I error due to multiple comparisons, we applied Bonferroni-corrected 95% CI. As a conservative approach, this correction widens confidence intervals and reduces the probability of false-positive findings.

To test the robustness of our results, we performed the following sensitivity analyses: (1) changed the degrees of freedom for temperature to 9; (2) additionally adjusted for the mean and standard deviation of summer and winter relative humidity; (3) additionally adjusted for annual concentrations of nitrogen dioxide and ozone; (4) identified hospitalizations based on the principal diagnosis code at discharge; (5) truncated negative non-wildfire PM_2.5_ values to zero in the models, rather than deleted the negative values; (6) assigned 3-year and 5-year moving averages of PM_2.5_ concentrations as alternative long-term exposure measures; (7) selected two, three, and 4 years before and after the year of hospitalization (case year) as control years, respectively; (8) to determine region-specific differences, we further performed stratified analyses by US state.

We further conducted stratified analyses based on individual-level factors (age, sex, race/ethnicity, patient location, comorbidity with obesity, and comorbidity with diabetes), disease severity (died/not died during hospitalization) and neighborhood-level characteristics (upper or lower quartiles of percentage of the population who graduated high school and TDI) to determine whether the associations varied across different groups. The differences between subgroups were assessed by the *Z*-test or the Wald test, as appropriate. All analyses were conducted by R (version 4.5.1) using “mgcv” packages.

### Reporting summary

Further information on research design is available in the Nature Portfolio Reporting Summary linked to this article.

## Supplementary information

Supplementary Information

Peer Review file

Reporting Summary

## Source data

Source Data

## Data Availability

The State Inpatient Databases (SID) are available upon request to the Healthcare Cost and Utilization Project (HCUP) upon application and approval, subject to data use agreements (https://hcup-us.ahrq.gov/db/state/siddbdocumentation.jsp). Access requires completion of a data use agreement and institutional approval through HCUP. The local-level wildfire-specific PM_2.5_ data are publicly available at https://github.com/echolab-stanford/daily-10km-smokePM. The originally predicted, grid level total PM_2.5_ data are publicly available at https://www.earthdata.nasa.gov/data/catalog/sedac-ciesin-sedac-aqdh-dapm25-us-1km-1.0. Meteorological data, including temperature, precipitation, and vapor pressure, are publicly available at https://daac.ornl.gov/DAYMET/guides/Daymet_Daily_V4.html. Other data are publicly available with sources described in the manuscript. Source data are provided with this paper.

## Code availability

The analysis code is publicly available on GitHub at https://github.com/yycome/Intro_CCS.
