# Supplementary material for: Cardiopulmonary hospitalization risks from wildfire-specific and non-wildfire PM2.5 in 20 US states
Source: Nat Commun. 2026 Sep 17;17:9349. doi: 10.1038/s41467-026-76974-7 (PMC13586224; doi:10.1038/s41467-026-76974-7)
Supplement: Supplementary file 1 [file 41467_2026_76974_MOESM1_ESM.pdf]

Cardiopulmonary hospitalization risks from wildfire and non-wildfire $PM_{2.5}$ in 20 US states

Supplementary Information

Min Zhang, PhD[1*]; Edgar Castro, PhD[2]; Minghao Qiu, PhD[3,4]; Mahdieh Danesh Yazdi, PhD[4]; Boyuan Li, PhD[5]; Rosalind J. Wright, MD, MPH[1,6]; Joel D. Schwartz, PhD[2,7]; Robert O. Wright, MD, MPH[1]; Yaguang Wei, PhD[1]

[1] Department of Environmental Medicine, Icahn School of Medicine at Mount Sinai, New York, NY, USA

[2] Department of Environmental Health, Harvard T.H. Chan School of Public Health, Boston, MA, USA

[3] School of Marine and Atmospheric Sciences, Stony Brook University, Stony Brook, NY, USA

[4] Program in Public Health, Department of Family, Population, and Preventive Medicine, Stony Brook University, Stony Brook, NY, USA

[5] Department of Chemical and Biomolecular Engineering, Tandon School of Engineering, New York University, Brooklyn, NY, USA

[6] Department of Public Health, Icahn School of Medicine at Mount Sinai, New York, NY, USA

[7] Department of Epidemiology, Harvard T.H. Chan School of Public Health, Boston, MA, USA.

*Corresponding Author: Min Zhang, PhD, Department of Environmental Medicine, Icahn School of Medicine at Mount Sinai, 17 E 102nd St, New York, NY 10029 (min.zhang@mssm.edu).

List of Supplementary Items

Supplementary Methods
Supplementary Table 1. Characteristics for hospitalized patients.
Supplementary Table 2. The distribution of 2-year averages of wildfire-specific $PM_{2.5}$ and non-wildfire $PM_{2.5}$ (μg/m^3).
Supplementary Table 3. The hospitalization risks of cardiopulmonary diseases associated with a SD increase in 2-year average of wildfire-specific $PM_{2.5}$ and non-wildfire $PM_{2.5}$ concentrations.
Supplementary Table 4. Sensitivity analysis results adjusting degrees of freedom for temperature to nine.
Supplementary Table 5. Sensitivity analysis results adjusting for annual precipitation and vapor pressure.
Supplementary Table 6. Sensitivity analysis results under case definition based on the principal diagnosis code at discharge.
Supplementary Table 7. Sensitivity analysis results using alternative approaches to handle negative values of non-wildfire $PM_{2.5}$.
Supplementary Table 8. Sensitivity analysis results using 3- or 4-year moving averages as alternative exposure windows.
Supplementary Table 9. Sensitivity analysis results using alternative control-year specifications.
Supplementary Table 10. Sensitivity analysis results stratifying the model by US state.
Supplementary Table 11. Subgroup analysis by age.
Supplementary Table 12. Subgroup analysis by sex.
Supplementary Table 13. Subgroup analysis by race/ethnicity.
Supplementary Table 14. Subgroup analysis by patient location.
Supplementary Table 15. Subgroup analysis by comorbidity status of obesity.
Supplementary Table 16. Subgroup analysis by comorbidity status of diabetes.
Supplementary Table 17. Subgroup analysis by severity of the disease.
Supplementary Table 18. Subgroup analysis by percentage of the population who graduated high school.
Supplementary Table 19. Subgroup analysis by townsend deprivation index.
Supplementary Table 20. Study periods of each state.
Supplementary Table 21. The ICD codes used for the disease identification in this study.

Supplementary Methods

In this study, we used conditional logistic regression models to estimate the effects of long-term wildfire-specific and non-wildfire $PM_{2.5}$ on the hospitalization risk, adjusting for measured time-varying confounders, including calendar year, temperature values, and neighborhood-level covariates.

In the conditional logistic regression model, the 2-year average wildfire-specific and non-wildfire $PM_{2.5}$ concentrations were simultaneously included as continuous variables to mutually adjust for each other's effects. Calendar year was included as a categorical variable to control for potential unmeasured confounders varying over time. Surface temperature variables–including mean summer temperature, standard deviation of summer temperature, mean winter temperature, and standard deviation of winter temperature–were modeled using penalized cubic splines with 5 degrees of freedom to account for potential nonlinear relationships. Other covariates included neighborhood-level statistics, such as percentage of White, percentage of Black, percentage of the population who graduated high school, median household income, and Townsend Deprivation Index, all adjusted as continuous variables. The model was provided below.

Let $l(\mu_1(\beta), \cdots, \mu_n(\beta))$ denotes the log likelihood for a conditional logistic regression for observations $i = 1, \ldots, n$, where

$$\mu_i(\beta) = \beta_1 \boldsymbol{W}_i + \beta_2 \boldsymbol{X}_i + \beta_T \boldsymbol{T}_i + \beta_Y \boldsymbol{Y}_i + \beta_N \boldsymbol{N}_i$$

$\boldsymbol{W}_{i,1}$ and $\boldsymbol{X}_{i,1}$ are the exposure of interest, respectively (2-year average wildfire-specific and non-wildfire $PM_{2.5}$ concentrations, respective), and $\boldsymbol{T}_i$ are the vectors of cubic spline expansions of air temperature. The cubic spline expansion has five degrees of freedom. $\boldsymbol{Y}_i$ and $\boldsymbol{N}_i$ represents calendar year and neighborhood-level statistics, respectively.

Supplementary Table 1. Characteristics for hospitalized patients.

Characteristics	Cardiovascular diseases (CVD)						Respiratory diseases (RD)				
	Ischemic heart disease	Cerebrovascular disease	Heart failure	Arrhythmia	Hypertension	Other cardiovascular diseases	Acute respiratory infections	Pneumonia	COPD	Asthma	Other respiratory diseases
Number of individuals, n (%)	8300356	5171077	9867248	7632375	15754362	10539548	1404055	7976711	6048248	3416258	13846122
Age, y, mean (SD)	68.5 (13.6)	69.6 (15.2)	72.8 (14.6)	71.4 (15.8)	65.2 (15.5)	64.9 (18.0)	26.3 (31.5)	64.5 (23.2)	68.8 (12.7)	40.9 (25.2)	63.8 (21.5)
Sex, n (%)											
Female	3278182 (39.5)	2650863 (51.3)	5004769 (50.7)	3660338 (48.0)	8229910 (52.2)	5225091 (49.6)	706010 (50.3)	4108802 (51.5)	3268094 (54.0)	2200754 (64.4)	7105455 (51.3)
Male	5021667 (60.5)	2519843 (48.7)	4861715 (49.3)	3971418 (52.0)	7522757 (47.8)	5313291 (50.4)	697943 (49.7)	3867166 (48.5)	2779638 (46.0)	1215106 (35.6)	6739172 (48.7)
Missing	507 (0.01)	371 (0.01)	764 (0.01)	619 (0.01)	1695 (0.01)	1166 (0.01)	102 (0.01)	743 (0.01)	516 (0.01)	398 (0.01)	1495 (0.01)
Race/ethnicity, n (%)											
White	5726781 (69.0)	3318759 (64.2)	6505047 (65.9)	5559589 (72.8)	9465162 (60.1)	6880790 (65.3)	692307 (49.3)	5403834 (67.7)	4525708 (74.8)	1620777 (47.4)	9414885 (68.0)
Black	848768 (10.2)	799576 (15.5)	1580404 (16.0)	697758 (9.1)	3024803 (19.2)	1578797 (15.0)	226230 (16.1)	958272 (12.0)	604069 (10.0)	803557 (23.5)	1693453 (12.2)
Hispanic	457647 (5.5)	296785 (5.7)	503589 (5.1)	325244 (4.3)	1154959 (7.3)	606597 (5.8)	211843 (15.1)	501809 (6.3)	192384 (3.2)	442716 (13.0)	833581 (6.0)
Other	499943 (6.0)	303494 (5.9)	448917 (4.5)	330025 (4.3)	938269 (6.0)	524991 (5.0)	121507 (8.7)	412588 (5.2)	189628 (3.1)	242964 (7.1)	732452 (5.3)
Missing	767217 (9.2)	452463 (8.7)	829291 (8.4)	719759 (9.4)	1171169 (7.4)	948373 (9.0)	152168 (10.8)	700208 (8.8)	536459 (8.9)	306244 (9.0)	1171751 (8.5)
Patient location, n (%)											
Metropolitan areas	6612443 (79.7)	4269116 (82.6)	7998579 (81.1)	6223616 (81.5)	13109443 (83.2)	8761543 (83.1)	1171856 (83.5)	6203349 (77.8)	4548566 (75.2)	2980905 (87.3)	11345511 (81.9)
Micropolitan areas	973852 (11.7)	530530 (10.3)	1086703 (11.0)	819194 (10.7)	1554821 (9.9)	1051594 (10.0)	132460 (9.4)	987304 (12.4)	814618 (13.5)	265354 (7.8)	1495092 (10.8)

Non-CBSA	710461 (8.6)	369970 (7.2)	779060 (7.9)	586538 (7.7)	1085088 (6.9)	723156 (6.9)	99417 (7.1)	784077 (9.8)	682621 (11.3)	168903 (4.9)	1002881 (7.2)
Missing	3600 (0.04)	1461 (0.03)	2906 (0.03)	3027 (0.04)	5010 (0.03)	3255 (0.03)	322 (0.02)	1981 (0.02)	2443 (0.04)	1096 (0.03)	2638 (0.02)
Died during hospitalization, n (%)	293836 (3.5)	258276 (5.0)	287764 (2.9)	179291 (2.3)	112885 (0.7)	273830 (2.6)	1423 (0.1)	409586 (5.1)	89868 (1.5)	8201 (0.2)	1317231 (9.5)
Comorbidity with obesity, n (%)	774415 (9.3)	306140 (5.9)	939909 (9.5)	655384 (8.6)	1740327 (11.0)	953261 (9.0)	66801 (4.8)	474999 (6.0)	569813 (9.4)	410053 (12.0)	1078374 (7.8)
Comorbidity with diabetes, n (%)	2560949 (30.9)	1422334 (27.5)	3233121 (32.8)	1722448 (22.6)	4323743 (27.4)	2337610 (22.2)	130744 (9.3)	1671658 (21.0)	1478897 (24.5)	470441 (13.8)	2622252 (18.9)
Neighborhood-level statistics, mean (SD)											
Percentage of White, %	71.2 (24.5)	68.3 (25.6)	68.3 (26.0)	72.5 (23.3)	65.5 (27.0)	68.8 (25.3)	63.9 (27.2)	70.4 (24.8)	72.9 (23.8)	60.8 (28.7)	69.2 (24.4)
Percentage of Black, %	12.7 (19.1)	14.7 (21.0)	15.3 (21.7)	11.8 (18.1)	16.7 (22.5)	14.4 (20.8)	16.1 (21.5)	13.2 (19.4)	13.0 (19.1)	19.8 (24.2)	13.2 (19.3)
Percentage of the population who graduated high school, %	85.6 (8.4)	85.9 (8.3)	85.5 (8.4)	86.7 (8.05)	85.2 (8.7)	86.1 (8.3)	83.8 (9.7)	85.2 (8.5)	84.8 (8.3)	83.5 (9.6)	86.0 (8.3)
Median household income, $	56726.8 (23379.9)	57187.2 (23627.4)	55516.5 (22987.9)	58990.5 (24311.3)	56687.8 (23728.4)	57508.0 (23889.9)	54716.8 (23244.8)	54949.8 (22452.5)	52519.0 (20819.6)	53429.9 (22936.5)	57081.7 (23316.3)
Townsend deprivation index	1.25 (3.15)	1.32 (3.12)	1.47 (3.25)	1.04 (2.98)	1.68 (3.52)	1.31 (3.16)	2.03 (3.76)	1.28 (3.05)	1.15 (2.80)	2.63 (4.24)	1.18 (2.88)
Environmental data, median (ranges)											
Wildfire-specific $PM_{2.5}$, μg/m^3	0.30 (0.00, 11.15)	0.29 (0.00, 11.15)	0.31 (0.00, 11.15)	0.30 (0.00, 11.15)	0.29 (0.00, 11.15)	0.30 (0.00. 11.15)	0.29 (0.00, 10.26)	0.30 (0.00, 11.15)	0.30 (0.00, 11.15)	0.29 (0.00, 10.26)	0.29 (0.00, 11.15)
Non-wildfire $PM_{2.5}$, μg/m^3	8.06 (0.02, 22.78)	7.96 (0.02, 20.06)	8.04 (0.02, 20.85)	7.94 (0.02, 20.67)	7.99 (0.005, 21.30)	7.95 (0.02, 20.67)	8.05 (0.006, 19.24)	7.96 (0.02, 20.85)	8.03 (0.005, 20.85)	8.32 (0.005, 20.85)	7.78 (0.005, 20.85)
Mean summer temperature, °C	22.89 (11.80, 35.53)	22.97 (11.44, 35.53)	22.93 (11.44, 35.53)	22.82 (11.44, 35.53)	23.14 (11.47, 35.53)	22.96 (11.44, 35.53)	23.07 (11.60, 35.53)	23.01 (11.44, 35.53)	22.90 (11.80, 35.53)	23.11 (11.60, 35.53)	22.96 (11.44, 35.53)
SD of summer temperature, °C	1.51 (0.001, 4.41)	1.49 (0.001, 4.35)	1.51 (0.001, 4.41)	1.50 (0.001, 4.46)	1.51 (0.001, 4.46)	1.50 (0.001, 4.33)	1.55 (0.001, 4.35)	1.47 (0.001, 4.46)	1.44 (0.001, 4.46)	1.58 (0.001, 4.35)	1.47 (0.001, 4.41)
Mean winter temperature, °C	1.46 (-19.26,	1.84 (-19.26,	1.49 (-19.22,	1.46 (-19.26,	1.84 (-19.26,	1.63 (-19.26,	1.69 (-19.22,	1.69 (-19.26,	1.43 (-19.26,	1.45 (-19.26,	1.98 (-19.26,

	23.71)	23.23)	23.71)	23.28)	23.71)	23.71)	23.19)	23.22)	23.22)	23.71)	23.71)
SD of summer temperature, °C	2.07 (0.005, 9.44)	2.08 (0.005, 9.50)	2.21 (0.005, 9.46)	2.13 (0.005, 9.60)	2.13 (0.005, 9.64)	2.13 (0.005, 9.61)	2.05 (0.005, 9.37)	2.13 (0.005, 9.44)	2.22 (0.005, 9.54)	2.06 (0.005, 9.42)	2.14 (0.005, 9.60)
Abbreviations: SD, standard deviation; non-CBSA, non-Core Based Statistical Areas.											

Supplementary Table 2. The distribution of 2-year averages of wildfire-specific $PM_{2.5}$ and non-wildfire $PM_{2.5}$ ($\mu g/m^3$).

Variables	Mean (SD)	Median (IQR)	Percentiles				
			P5	P25	P75	P95	Min-Max
Ischemic heart disease							
Wildfire-specific $PM_{2.5}$	0.376 (0.336)	0.322 (0.261)	0.085	0.200	0.461	0.721	0.004-7.787
Non-wildfire $PM_{2.5}$	8.292 (2.124)	8.271 (2.568)	4.604	6.998	9.567	11.92	0.290-21.566
Cerebrovascular disease							
Wildfire-specific $PM_{2.5}$	0.383 (0.360)	0.318 (0.266)	0.086	0.196	0.462	0.773	0.004-7.787
Non-wildfire $PM_{2.5}$	8.156 (2.061)	8.154 (2.409)	4.570	6.936	9.345	11.723	0.290-20.687
Heart failure							
Wildfire-specific $PM_{2.5}$	0.386 (0.346)	0.328 (0.264)	0.095	0.205	0.469	0.750	0.004-7.787
Non-wildfire $PM_{2.5}$	8.250 (2.049)	8.242 (2.412)	4.739	7.013	9.425	11.819	0.290-20.195
Arrhythmia							
Wildfire-specific $PM_{2.5}$	0.378 (0.340)	0.322 (0.263)	0.082	0.199	0.462	0.733	0.004-7.787
Non-wildfire $PM_{2.5}$	8.134 (2.084)	8.124 (2.463)	4.509	6.889	9.352	11.754	0.290-20.687
Hypertension							
Wildfire-specific $PM_{2.5}$	0.375 (0.342)	0.315 (0.255)	0.094	0.199	0.454	0.734	0.004-7.787
Non-wildfire $PM_{2.5}$	8.266 (2.059)	8.210 (2.454)	4.782	7.013	9.467	11.854	0.290-19.797
Other cardiovascular diseases							
Wildfire-specific $PM_{2.5}$	0.379 (0.349)	0.318 (0.265)	0.084	0.196	0.461	0.747	0.004-7.787
Non-wildfire $PM_{2.5}$	8.148 (2.055)	8.142 (2.401)	4.576	6.934	9.335	11.710	0.290-20.195
Acute respiratory infections							
Wildfire-specific $PM_{2.5}$	0.371 (0.333)	0.315 (0.266)	0.080	0.194	0.459	0.723	0.004-7.787
Non-wildfire $PM_{2.5}$	8.300 (2.061)	8.259 (2.446)	4.794	7.054	9.500	11.870	0.290-19.414
Pneumonia							
Wildfire-specific $PM_{2.5}$	0.379 (0.351)	0.319 (0.270)	0.084	0.194	0.465	0.759	0.004-7.787
Non-wildfire $PM_{2.5}$	8.162 (2.087)	8.159 (2.454)	4.527	6.922	9.376	11.766	0.290-20.175
COPD							
Wildfire-specific $PM_{2.5}$	0.370 (0.330)	0.319 (0.261)	0.086	0.198	0.459	0.710	0.004-7.787
Non-wildfire $PM_{2.5}$	8.276 (2.124)	8.248 (2.532)	4.612	6.989	9.521	11.947	0.290-20.195
Asthma							
Wildfire-specific $PM_{2.5}$	0.364 (0.297)	0.319 (0.258)	0.084	0.199	0.457	0.682	0.004-7.787
Non-wildfire $PM_{2.5}$	8.527 (2.022)	8.501 (2.399)	5.015	7.325	9.725	11.929	0.290-19.586
Other respiratory diseases							
Wildfire-specific $PM_{2.5}$	0.392 (0.396)	0.317 (0.272)	0.084	0.194	0.465	0.817	0.004-7.787
Non-wildfire $PM_{2.5}$	7.981 (2.044)	7.952 (2.356)	4.478	6.783	9.139	11.614	0.290-19.797

Abbreviations: SD, standard deviation; IQR, interquartile Range; COPD, chronic obstructive pulmonary disease.

Supplementary Table 3. The hospitalization risks of cardiopulmonary diseases associated with a SD increase in 2-year average of wildfire-specific $PM_{2.5}$ and non-wildfire $PM_{2.5}$ concentrations.

Outcomes	Wildfire-specific $PM_{2.5}$		Non-wildfire $PM_{2.5}$	
	SD, $\mu g/m^3$	Relative risk (95% CI)	SD, $\mu g/m^3$	Relative risk (95% CI)
Ischemic heart disease	0.336	1.042 (1.039, 1.045)	2.124	1.143 (1.135, 1.151)
Cerebrovascular disease	0.360	1.045 (1.041, 1.049)	2.061	1.152 (1.142, 1.162)
Heart failure	0.346	1.033 (1.031, 1.036)	2.049	1.115 (1.108, 1.122)
Arrhythmia	0.340	1.038 (1.035, 1.042)	2.084	1.111 (1.103, 1.119)
Hypertension	0.342	1.040 (1.038, 1.042)	2.059	1.183 (1.177, 1.189)
Other cardiovascular diseases	0.349	1.040 (1.037, 1.042)	2.055	1.133 (1.126, 1.139)
Acute respiratory infections	0.333	1.036 (1.028, 1.043)	2.061	1.114 (1.095, 1.133)
Pneumonia	0.351	1.037 (1.034, 1.040)	2.087	1.121 (1.113, 1.128)
COPD	0.330	1.036 (1.032, 1.039)	2.124	1.101 (1.092, 1.111)
Asthma	0.297	1.045 (1.040, 1.050)	2.022	1.125 (1.113, 1.137)
Other respiratory diseases	0.396	1.044 (1.041, 1.046)	2.044	1.139 (1.133, 1.145)
Ischemic heart disease	0.336	1.042 (1.039, 1.045)	2.124	1.143 (1.135, 1.151)

Abbreviations: SD, standard deviation; COPD, chronic obstructive pulmonary disease.

Note: The model was adjusted for calendar year, percentage of White, percentage of Black, percentage of the population who graduated high school, median household income, townsend deprivation index, mean temperature of summer and winter, and standard deviation of summer and winter temperature, annual NO2 concentration, and annual O3 concentration. All statistical tests were two-sided.

Supplementary Table 4. Sensitivity analysis results adjusting degrees of freedom for temperature to nine.

Outcome	Relative risk (95% CI) per 1 μg/m³ increase		Relative risk (95% CI) per SD μg/m³ increase	
	Wildfire-specific $PM_{2.5}$	Non-wildfire $PM_{2.5}$	Wildfire-specific $PM_{2.5}$	Non-wildfire $PM_{2.5}$
**CVD**				
Ischemic heart disease	1.110 (1.100, 1.120)	1.061 (1.057, 1.064)	1.036 (1.033, 1.039)	1.133 (1.125, 1.142)
Cerebrovascular disease	1.117 (1.105, 1.129)	1.067 (1.062, 1.071)	1.041 (1.037, 1.045)	1.142 (1.132, 1.152)
Heart failure	1.091 (1.082, 1.099)	1.053 (1.050, 1.056)	1.030 (1.028, 1.033)	1.112 (1.104, 1.119)
Arrhythmia	1.102 (1.092, 1.112)	1.049 (1.045, 1.052)	1.034 (1.031, 1.037)	1.104 (1.096, 1.112)
Hypertension	1.111 (1.105, 1.118)	1.077 (1.075, 1.080)	1.037 (1.035, 1.039)	1.166 (1.160, 1.172)
Other cardiovascular diseases	1.103 (1.095, 1.111)	1.059 (1.055, 1.062)	1.035 (1.032, 1.038)	1.124 (1.117, 1.131)
**RD**				
Acute respiratory infections	1.098 (1.074, 1.123)	1.050 (1.041, 1.059)	1.032 (1.024, 1.039)	1.106 (1.087, 1.124)
Pneumonia	1.100 (1.091, 1.109)	1.054 (1.050, 1.058)	1.034 (1.031, 1.037)	1.116 (1.108, 1.124)
COPD	1.100 (1.089, 1.112)	1.046 (1.042, 1.050)	1.032 (1.029, 1.036)	1.100 (1.090, 1.109)
Asthma	1.135 (1.118, 1.153)	1.055 (1.050, 1.061)	1.038 (1.034, 1.043)	1.115 (1.103, 1.127)
Other respiratory diseases	1.108 (1.102, 1.115)	1.063 (1.060, 1.066)	1.041 (1.039, 1.044)	1.133 (1.127, 1.139)

Abbreviations: CVD, cardiovascular diseases; RD, respiratory diseases; COPD, chronic obstructive pulmonary disease.

The model was adjusted for calendar year, percentage of White, percentage of Black, percentage of the population who graduated high school, median household income, townsend deprivation index, mean temperature of summer and winter, and standard deviation of summer and winter temperature. All statistical tests were two-sided.

Supplementary Table 5. Sensitivity analysis results adjusting for annual precipitation and vapor pressure.

Outcome	Relative risk (95% CI) per 1 μg/m^3 increase		Relative risk (95% CI) per SD μg/m^3 increase	
**CVD**	Wildfire-specific $PM_{2.5}$	Non-wildfire $PM_{2.5}$	Wildfire-specific $PM_{2.5}$	Non-wildfire $PM_{2.5}$
Ischemic heart disease	1.105 (1.095, 1.115)	1.062 (1.058, 1.066)	1.034 (1.031, 1.037)	1.136 (1.128, 1.144)
Cerebrovascular disease	1.112 (1.100, 1.124)	1.069 (1.065, 1.074)	1.039 (1.035, 1.043)	1.148 (1.138, 1.158)
Heart failure	1.083 (1.074, 1.092)	1.054 (1.051, 1.057)	1.028 (1.025, 1.031)	1.114 (1.107, 1.121)
Arrhythmia	1.100 (1.090, 1.110)	1.050 (1.047, 1.054)	1.033 (1.030, 1.036)	1.107 (1.099, 1.115)
Hypertension	1.096 (1.090, 1.103)	1.082 (1.079, 1.085)	1.032 (1.030, 1.034)	1.176 (1.171, 1.183)
Other cardiovascular diseases	1.102 (1.094, 1.111)	1.061 (1.058, 1.064)	1.035 (1.032, 1.037)	1.130 (1.123, 1.137)
**RD**				
Acute respiratory infections	1.103 (1.078, 1.128)	1.052 (1.044, 1.061)	1.033 (1.025, 1.041)	1.111 (1.092, 1.129)
Pneumonia	1.096 (1.087, 1.106)	1.055 (1.052, 1.059)	1.033 (1.030, 1.036)	1.119 (1.111, 1.127)
COPD	1.093 (1.081, 1.104)	1.045 (1.040, 1.049)	1.030 (1.026, 1.033)	1.097 (1.088, 1.107)
Asthma	1.134 (1.116, 1.152)	1.056 (1.051, 1.062)	1.038 (1.033, 1.043)	1.117 (1.105, 1.129)
Other respiratory diseases	1.101 (1.094, 1.108)	1.066 (1.063, 1.069)	1.039 (1.036, 1.041)	1.139 (1.133, 1.145)

Abbreviations: CVD, cardiovascular diseases; RD, respiratory diseases; COPD, chronic obstructive pulmonary disease.

The model was adjusted for calendar year, percentage of White, percentage of Black, percentage of the population who graduated high school, median household income, townsend deprivation index, mean temperature of summer and winter, standard deviation of summer and winter temperature, annual precipitation, and annual vapor pressure. Daily precipitation vapor pressure (Pa) were derived from Daymet V4. All statistical tests were two-sided.

Supplementary Table 6. Sensitivity analysis results under case definition based on the principal diagnosis code at discharge.

Outcome	Relative risk (95% CI) per 1 μg/m^3 increase		Relative risk (95% CI) per SD μg/m^3 increase	
	Wildfire-specific $PM_{2.5}$	Non-wildfire $PM_{2.5}$	Wildfire-specific $PM_{2.5}$	Non-wildfire $PM_{2.5}$
**CVD**				
Ischemic heart disease	1.150 (1.136, 1.164)	1.071 (1.066, 1.076)	1.048 (1.044, 1.052)	1.157 (1.146, 1.168)
Cerebrovascular disease	1.137 (1.124, 1.151)	1.079 (1.073, 1.084)	1.047 (1.043, 1.052)	1.169 (1.157, 1.181)
Heart failure	1.092 (1.072, 1.113)	1.020 (1.014, 1.027)	1.031 (1.024, 1.038)	1.042 (1.030, 1.055)
Arrhythmia	1.118 (1.102, 1.135)	1.057 (1.051, 1.062)	1.039 (1.034, 1.044)	1.121 (1.109, 1.134)
Hypertension	1.278 (1.259, 1.297)	1.148 (1.140, 1.156)	1.088 (1.082, 1.093)	1.329 (1.310, 1.348)
Other cardiovascular diseases	1.144 (1.130, 1.157)	1.069 (1.064, 1.074)	1.048 (1.044, 1.052)	1.147 (1.135, 1.158)
**RD**				
Acute respiratory infections	1.125 (1.094, 1.158)	1.052 (1.041, 1.063)	1.040 (1.030, 1.050)	1.111 (1.087, 1.135)
Pneumonia	1.086 (1.070, 1.101)	1.049 (1.044, 1.055)	1.029 (1.024, 1.034)	1.105 (1.093, 1.117)
COPD	1.141 (1.122, 1.160)	1.060 (1.053, 1.067)	1.044 (1.039, 1.050)	1.132 (1.117, 1.148)
Asthma	1.158 (1.126, 1.191)	1.056 (1.046, 1.065)	1.045 (1.036, 1.053)	1.116 (1.095, 1.137)
Other respiratory diseases	1.117 (1.104, 1.130)	1.071 (1.066, 1.076)	1.045 (1.040, 1.049)	1.150 (1.139, 1.161)

Abbreviations: CVD, cardiovascular diseases; RD, respiratory diseases; COPD, chronic obstructive pulmonary disease.

The model was adjusted for calendar year, percentage of White, percentage of Black, percentage of the population who graduated high school, median household income, townsend deprivation index, mean temperature of summer and winter, and standard deviation of summer and winter temperature. All statistical tests were two-sided.

Supplementary Table 7. Sensitivity analysis results using alternative approaches to handle negative values of non-wildfire $PM_{2.5}$.

Outcomes [a]	Relative risk (95% CI) per 1 μg/m^3 increase		Relative risk (95% CI) per SD μg/m^3 increase	
	Wildfire-specific $PM_{2.5}$	Non-wildfire $PM_{2.5}$	Wildfire-specific $PM_{2.5}$	Non-wildfire $PM_{2.5}$
**CVD**				
Ischemic heart disease	1.128 (1.118, 1.138)	1.065 (1.062, 1.069)	1.041 (1.038, 1.044)	1.143 (1.135, 1.151)
Cerebrovascular disease	1.130 (1.118, 1.141)	1.071 (1.067, 1.076)	1.045 (1.041, 1.049)	1.153 (1.143, 1.163)
Heart failure	1.099 (1.091, 1.108)	1.055 (1.051, 1.058)	1.033 (1.030, 1.036)	1.115 (1.108, 1.123)
Arrhythmia	1.116 (1.107, 1.126)	1.052 (1.048, 1.055)	1.038 (1.035, 1.041)	1.111 (1.103, 1.119)
Hypertension	1.122 (1.115, 1.129)	1.085 (1.082, 1.088)	1.040 (1.038, 1.042)	1.183 (1.177, 1.189)
Other cardiovascular diseases	1.116 (1.108, 1.125)	1.063 (1.059, 1.066)	1.039 (1.037, 1.042)	1.133 (1.126, 1.140)
**RD**				
Acute respiratory infections	1.111 (1.087, 1.136)	1.054 (1.045, 1.062)	1.036 (1.028, 1.043)	1.114 (1.095, 1.133)
Pneumonia	1.108 (1.099, 1.118)	1.056 (1.053, 1.060)	1.037 (1.034, 1.040)	1.121 (1.113, 1.129)
COPD	1.112 (1.101, 1.123)	1.047 (1.042, 1.051)	1.036 (1.032, 1.039)	1.102 (1.092, 1.111)
Asthma	1.159 (1.141, 1.177)	1.060 (1.054, 1.066)	1.045 (1.040, 1.050)	1.125 (1.113, 1.137)
Other respiratory diseases	1.114 (1.108, 1.120)	1.066 (1.063, 1.069)	1.044 (1.041, 1.046)	1.139 (1.134, 1.145)

Abbreviations: CVD, cardiovascular diseases; RD, respiratory diseases; COPD, chronic obstructive pulmonary disease.

Note: The model was adjusted for calendar year, percentage of White, percentage of Black, percentage of the population who graduated high school, median household income, townsend deprivation index, mean temperature of summer and winter, and standard deviation of summer and winter temperature. All statistical tests were two-sided.

[a] The negative values of non-wildfire $PM_{2.5}$ resulting from the subtraction were treated as zero.

Supplementary Table 8. Sensitivity analysis results using 3- or 4-year moving averages as alternative exposure windows.

Outcomes	Relative risk (95% CI) per 1 μg/m^3 increase		Relative risk (95% CI) per SD μg/m^3 increase	
	Wildfire-specific $PM_{2.5}$	Non-wildfire $PM_{2.5}$	Wildfire-specific $PM_{2.5}$	Non-wildfire $PM_{2.5}$
Lag02 (3-year averages)				
Ischemic heart disease	1.372 (1.352, 1.393)	1.036 (1.031, 1.040)	1.112 (1.107, 1.118)	1.080 (1.070, 1.091)
Cerebrovascular disease	1.425 (1.400, 1.450)	1.032 (1.026, 1.037)	1.136 (1.129, 1.143)	1.070 (1.058, 1.082)
Heart failure	1.319 (1.301, 1.337)	1.030 (1.025, 1.034)	1.101 (1.096, 1.106)	1.065 (1.056, 1.074)
Arrhythmia	1.367 (1.346, 1.387)	1.024 (1.019, 1.028)	1.112 (1.106, 1.118)	1.053 (1.043, 1.063)
Hypertension	1.409 (1.394, 1.424)	1.037 (1.034, 1.041)	1.125 (1.121, 1.129)	1.083 (1.076, 1.091)
Other cardiovascular diseases	1.369 (1.352, 1.386)	1.027 (1.023, 1.031)	1.116 (1.111, 1.121)	1.060 (1.052, 1.069)
Acute respiratory infections	1.442 (1.389, 1.497)	1.038 (1.027, 1.049)	1.130 (1.116, 1.144)	1.084 (1.06, 1.1090)
Pneumonia	1.334 (1.314, 1.353)	1.020 (1.015, 1.024)	1.106 (1.101, 1.112)	1.045 (1.035, 1.055)
COPD	1.267 (1.245, 1.288)	1.013 (1.008, 1.019)	1.081 (1.075, 1.087)	1.031 (1.020, 1.043)
Asthma	1.472 (1.434, 1.511)	1.042 (1.035, 1.050)	1.122 (1.113, 1.130)	1.090 (1.075, 1.106)
Other respiratory diseases	1.408 (1.393, 1.422)	1.029 (1.025, 1.032)	1.145 (1.141, 1.150)	1.064 (1.056, 1.071)
Ischemic heart disease	1.372 (1.352, 1.393)	1.036 (1.031, 1.040)	1.112 (1.107, 1.118)	1.080 (1.070, 1.091)
Lag03 (4-year averages)				
Ischemic heart disease	1.560 (1.531, 1.590)	1.066 (1.060, 1.072)	1.161 (1.154, 1.169)	1.150 (1.136, 1.164)
Cerebrovascular disease	1.590 (1.555, 1.625)	0.991 (0.987, 0.996)	1.175 (1.166, 1.184)	1.149 (1.133, 1.166)
Heart failure	1.550 (1.524, 1.575)	1.053 (1.048, 1.059)	1.164 (1.157, 1.170)	1.117 (1.105, 1.129)
Arrhythmia	1.526 (1.498, 1.554)	1.055 (1.049, 1.061)	1.154 (1.147, 1.162)	1.122 (1.108, 1.136)
Hypertension	1.676 (1.655, 1.698)	1.082 (1.078, 1.087)	1.193 (1.188, 1.199)	1.183 (1.172, 1.193)
Other cardiovascular diseases	1.540 (1.517, 1.564)	1.062 (1.056, 1.067)	1.163 (1.156, 1.169)	1.135 (1.124, 1.147)
Acute respiratory infections	1.634 (1.560, 1.711)	1.068 (1.053, 1.082)	1.178 (1.160, 1.196)	1.149 (1.117, 1.182)
Pneumonia	1.591 (1.563, 1.620)	1.042 (1.036, 1.048)	1.177 (1.170, 1.184)	1.093 (1.081, 1.106)
COPD	1.497 (1.466, 1.529)	1.025 (1.018, 1.032)	1.143 (1.135, 1.150)	1.057 (1.042, 1.072)
Asthma	1.688 (1.633, 1.745)	1.069 (1.059, 1.079)	1.168 (1.157, 1.180)	1.147 (1.126, 1.169)
Other respiratory diseases	1.583 (1.564, 1.602)	1.055 (1.051, 1.060)	1.200 (1.194, 1.205)	1.122 (1.113, 1.132)
Ischemic heart disease	1.560 (1.531, 1.590)	1.066 (1.060, 1.072)	1.161 (1.154, 1.169)	1.150 (1.136, 1.164)

Abbreviations: COPD, chronic obstructive pulmonary disease.

Note: The model was adjusted for calendar year, percentage of White, percentage of Black, percentage of the population who graduated high school, median household income, townsend deprivation index, mean temperature of summer and winter, and standard deviation of summer and winter temperature. All statistical tests were two-sided.

Supplementary Table 9. Sensitivity analysis results using alternative control-year specifications.

Outcomes	Relative risk (95% CI) per 1 μg/m^3 increase		Relative risk (95% CI) per SD μg/m^3 increase	
	Wildfire-specific $PM_{2.5}$	Non-wildfire $PM_{2.5}$	Wildfire-specific $PM_{2.5}$	Non-wildfire $PM_{2.5}$
Two years before and after the year of hospitalization (case year)				
Ischemic heart disease	1.200 (1.192, 1.208)	1.071 (1.069, 1.074)	1.063 (1.061, 1.066)	1.157 (1.152, 1.163)
Cerebrovascular disease	1.189 (1.180, 1.198)	1.078 (1.075, 1.081)	1.064 (1.061, 1.067)	1.167 (1.160, 1.174)
Heart failure	1.082 (1.076, 1.088)	1.062 (1.060, 1.064)	1.028 (1.026, 1.030)	1.131 (1.126, 1.137)
Arrhythmia	1.152 (1.144, 1.159)	1.047 (1.044, 1.049)	1.049 (1.047, 1.052)	1.100 (1.094, 1.105)
Hypertension	1.254 (1.248, 1.260)	1.082 (1.080, 1.084)	1.080 (1.079, 1.082)	1.175 (1.171, 1.180)
Other cardiovascular diseases	1.160 (1.154, 1.167)	1.064 (1.062, 1.066)	1.053 (1.051, 1.055)	1.137 (1.132, 1.141)
Acute respiratory infections	1.229 (1.209, 1.249)	1.069 (1.063, 1.075)	1.071 (1.065, 1.077)	1.148 (1.135, 1.162)
Pneumonia	1.170 (1.162, 1.177)	1.062 (1.060, 1.065)	1.057 (1.054, 1.059)	1.135 (1.129, 1.140)
COPD	1.161 (1.152, 1.170)	1.037 (1.034, 1.040)	1.050 (1.048, 1.053)	1.080 (1.073, 1.086)
Asthma	1.241 (1.227, 1.255)	1.068 (1.065, 1.072)	1.066 (1.063, 1.070)	1.143 (1.135, 1.152)
Other respiratory diseases	1.183 (1.178, 1.189)	1.079 (1.077, 1.081)	1.069 (1.067, 1.071)	1.168 (1.164, 1.172)
Ischemic heart disease	1.200 (1.192, 1.208)	1.071 (1.069, 1.074)	1.063 (1.061, 1.066)	1.157 (1.152, 1.163)
Three years before and after the year of hospitalization (case year)				
Ischemic heart disease	1.264 (1.256, 1.271)	1.080 (1.078, 1.082)	1.082 (1.080, 1.084)	1.177 (1.172, 1.182)
Cerebrovascular disease	1.295 (1.286, 1.304)	1.064 (1.061, 1.066)	1.098 (1.095, 1.100)	1.136 (1.130, 1.141)
Heart failure	1.312 (1.305, 1.319)	1.076 (1.074, 1.078)	1.099 (1.097, 1.101)	1.163 (1.158, 1.167)
Arrhythmia	1.292 (1.284, 1.299)	1.064 (1.062, 1.066)	1.091 (1.089, 1.093)	1.138 (1.133, 1.142)
Hypertension	1.256 (1.250, 1.261)	1.096 (1.095, 1.098)	1.081 (1.079, 1.083)	1.209 (1.205, 1.212)
Other cardiovascular diseases	1.241 (1.234, 1.247)	1.057 (1.055, 1.059)	1.078 (1.076, 1.080)	1.121 (1.117, 1.125)
Acute respiratory infections	1.215 (1.197, 1.233)	1.077 (1.072, 1.082)	1.067 (1.062, 1.072)	1.165 (1.154, 1.177)
Pneumonia	1.264 (1.257, 1.271)	1.048 (1.046, 1.050)	1.086 (1.084, 1.088)	1.103 (1.098, 1.107)
COPD	1.253 (1.245, 1.262)	1.055 (1.053, 1.058)	1.077 (1.075, 1.080)	1.121 (1.116, 1.127)
Asthma	1.274 (1.261, 1.287)	1.056 (1.053, 1.060)	1.075 (1.071, 1.078)	1.118 (1.110, 1.125)
Other respiratory diseases	1.308 (1.302, 1.313)	1.070 (1.068, 1.071)	1.112 (1.110, 1.114)	1.148 (1.144, 1.151)
Ischemic heart disease	1.264 (1.256, 1.271)	1.080 (1.078, 1.082)	1.082 (1.080, 1.084)	1.177 (1.172, 1.182)
Four years before and after the year of hospitalization (case year)				
Ischemic heart disease	1.222 (1.215, 1.229)	1.085 (1.083, 1.087)	1.070 (1.068, 1.072)	1.190 (1.185, 1.195)
Cerebrovascular disease	1.257 (1.249, 1.266)	1.062 (1.059, 1.064)	1.086 (1.083, 1.088)	1.131 (1.126, 1.137)
Heart failure	1.340 (1.334, 1.347)	1.065 (1.063, 1.067)	1.107 (1.105, 1.109)	1.138 (1.134, 1.142)
Arrhythmia	1.273 (1.266, 1.281)	1.049 (1.047, 1.051)	1.086 (1.084, 1.088)	1.104 (1.100, 1.109)
Hypertension	1.181 (1.176, 1.186)	1.123 (1.121, 1.125)	1.059 (1.057, 1.060)	1.270 (1.266, 1.273)
Other cardiovascular diseases	1.220 (1.215, 1.226)	1.058 (1.056, 1.060)	1.072 (1.070, 1.074)	1.122 (1.118, 1.126)
Acute respiratory infections	1.160 (1.144, 1.176)	1.094 (1.089, 1.099)	1.051 (1.046, 1.056)	1.202 (1.191, 1.214)
Pneumonia	1.248 (1.242, 1.255)	1.040 (1.038, 1.042)	1.081 (1.079, 1.083)	1.086 (1.082, 1.090)

COPD	1.163 (1.156, 1.171)	1.041 (1.039, 1.044)	1.051 (1.049, 1.053)	1.090 (1.085, 1.095)
Asthma	1.230 (1.218, 1.241)	1.064 (1.061, 1.067)	1.063 (1.060, 1.066)	1.134 (1.127, 1.141)
Other respiratory diseases	1.303 (1.297, 1.308)	1.073 (1.071, 1.074)	1.110 (1.109, 1.112)	1.155 (1.151, 1.158)
Ischemic heart disease	1.222 (1.215, 1.229)	1.085 (1.083, 1.087)	1.070 (1.068, 1.072)	1.190 (1.185, 1.195)

Abbreviations: COPD, chronic obstructive pulmonary disease.

Note: The model was adjusted for calendar year, percentage of White, percentage of Black, percentage of the population who graduated high school, median household income, townsend deprivation index, mean temperature of summer and winter, and standard deviation of summer and winter temperature. All statistical tests were two-sided.

Supplementary Table 10. Sensitivity analysis results stratifying the model by US state.

States	Cardiovascular diseases (CVD)						Respiratory diseases (RD)				
	Ischemic heart disease	Cerebrovascular disease	Heart failure	Arrhythmia	Hypertension	Other cardiovascular diseases	Acute respiratory infections	Pneumonia	COPD	Asthma	Other respiratory diseases
Wildfire-specific $PM_{2.5}$											
AZ	1.052 (0.953, 1.162)	1.051 (0.936, 1.18)	0.997 (0.894, 1.112)	1.059 (0.963, 1.164)	1.007 (0.929, 1.091)	1.033 (0.946, 1.128)	1.299 (0.987, 1.708)	0.996 (0.904, 1.098)	1.022 (0.901, 1.159)	1.122 (0.917, 1.373)	1.026 (0.955, 1.101)
CA	1.084 (1.034, 1.137)	1.354 (1.29, 1.422)	1.101 (1.057, 1.147)	1.058 (1.01, 1.108)	1.062 (1.029, 1.096)	1.049 (1.01, 1.089)	1.034 (0.927, 1.153)	1.238 (1.188, 1.289)	1.154 (1.088, 1.224)	1.022 (0.935, 1.116)	1.234 (1.202, 1.266)
CO	0.541 (0.471, 0.622)	0.936 (0.746, 1.175)	0.277 (0.237, 0.324)	0.288 (0.249, 0.334)	1.016 (0.869, 1.186)	1.022 (0.876, 1.193)	0.852 (0.56, 1.297)	0.961 (0.787, 1.174)	0.888 (0.674, 1.169)	0.997 (0.715, 1.391)	1.048 (0.929, 1.183)
DE	1.056 (0.232, 4.812)	0.606 (0.041, 9.006)	2.133 (0.256, 17.795)	0.645 (0.064, 6.48)	1.292 (0.256, 6.515)	0.268 (0.034, 2.129)	0.728 (0.011, 46.844)	2.089 (0.365, 11.944)	1.178 (0.044, 31.891)	1.897 (0.055, 64.926)	1.621 (0.352, 7.471)
GA	1.066 (0.997, 1.14)	1.006 (0.929, 1.089)	1.027 (0.968, 1.089)	1.058 (0.985, 1.135)	0.879 (0.848, 0.911)	1.034 (0.977, 1.094)	1.22 (1.041, 1.431)	0.974 (0.917, 1.035)	0.871 (0.825, 0.919)	0.912 (0.816, 1.019)	0.989 (0.942, 1.039)
IA	0.798 (0.627, 1.016)	0.781 (0.567, 1.077)	0.763 (0.603, 0.964)	0.762 (0.589, 0.985)	0.935 (0.761, 1.15)	0.957 (0.757, 1.211)	1.077 (0.55, 2.112)	0.831 (0.667, 1.037)	0.865 (0.656, 1.14)	1.07 (0.649, 1.764)	0.945 (0.776, 1.15)
IN	0.974 (0.521, 1.821)	1.242 (0.637, 2.421)	1.191 (0.676, 2.1)	0.843 (0.454, 1.565)	0.927 (0.599, 1.433)	0.847 (0.495, 1.45)	0.61 (0.106, 3.507)	1.029 (0.593, 1.785)	0.529 (0.249, 1.125)	1.003 (0.261, 3.847)	1.023 (0.709, 1.477)
KY	1.044 (0.901, 1.21)	0.915 (0.744, 1.126)	1.077 (0.938, 1.237)	0.882 (0.745, 1.045)	0.967 (0.855, 1.092)	0.924 (0.797, 1.07)	0.777 (0.537, 1.125)	0.985 (0.861, 1.127)	0.978 (0.853, 1.122)	0.755 (0.566, 1.008)	1.018 (0.907, 1.143)
MD	0.874 (0.234, 3.27)	1.746 (0.476, 6.4)	0.533 (0.087, 3.248)	3.35 (0.945, 11.871)	1.318 (0.495, 3.512)	1.471 (0.66, 3.282)	9.272 (0.04, 2159.764)	2.056 (0.494, 8.56)	2.185 (0.233, 20.523)	0.652 (0.063, 6.716)	0.853 (0.397, 1.829)
MI	0.874 (0.754, 1.014)	1.08 (0.889, 1.312)	1.033 (0.892,	1.012 (0.858,	0.678 (0.601,	0.937 (0.813,	1.126 (0.759,	1.062 (0.905,	0.91 (0.757,	0.961 (0.74,	1.01 (0.888,

			1.196)	1.192)	0.765)	1.08)	1.672)	1.247)	1.094)	1.249)	1.148)
MN	1.087 (0.918, 1.288)	0.935 (0.763, 1.145)	1.029 (0.899, 1.179)	1.015 (0.878, 1.174)	1.052 (0.928, 1.192)	1.086 (0.971, 1.214)	1.34 (0.868, 2.068)	0.954 (0.812, 1.121)	0.953 (0.759, 1.196)	1.175 (0.861, 1.604)	1.017 (0.907, 1.14)
NC	1.01 (0.941, 1.085)	0.999 (0.914, 1.091)	0.972 (0.91, 1.038)	0.988 (0.918, 1.063)	0.977 (0.927, 1.03)	0.881 (0.827, 0.938)	1.099 (0.907, 1.331)	0.984 (0.917, 1.055)	0.925 (0.854, 1.002)	1.048 (0.924, 1.187)	0.981 (0.93, 1.034)
NE	0.913 (0.606, 1.376)	0.872 (0.561, 1.355)	0.742 (0.513, 1.075)	0.798 (0.54, 1.18)	0.771 (0.572, 1.038)	0.612 (0.44, 0.851)	1.588 (0.612, 4.119)	0.786 (0.551, 1.121)	0.796 (0.497, 1.274)	0.302 (0.116, 0.783)	0.732 (0.581, 0.923)
NJ	0.856 (0.7, 1.046)	0.784 (0.592, 1.038)	0.857 (0.685, 1.073)	0.978 (0.784, 1.219)	0.758 (0.649, 0.886)	0.838 (0.688, 1.021)	0.781 (0.462, 1.32)	0.759 (0.598, 0.963)	0.845 (0.64, 1.116)	0.764 (0.557, 1.047)	0.785 (0.645, 0.956)
NY	0.991 (0.922, 1.065)	0.995 (0.901, 1.099)	1.184 (1.101, 1.274)	1.093 (1.012, 1.181)	1.057 (1.002, 1.116)	1.032 (0.964, 1.105)	1.127 (0.932, 1.364)	0.935 (0.859, 1.017)	1.128 (1.025, 1.241)	1.107 (0.993, 1.233)	0.92 (0.858, 0.987)
OR	1.034 (1.01, 1.059)	0.998 (0.969, 1.029)	0.997 (0.976, 1.018)	0.984 (0.961, 1.007)	0.982 (0.965, 0.999)	0.998 (0.978, 1.018)	1.031 (0.963, 1.104)	1.013 (0.989, 1.038)	1.02 (0.992, 1.048)	1.011 (0.965, 1.06)	1.018 (1.001, 1.034)
RI	1.152 (0.571, 2.324)	1.226 (0.438, 3.432)	0.52 (0.219, 1.234)	0.965 (0.447, 2.083)	1.021 (0.602, 1.731)	0.89 (0.404, 1.964)	0.605 (0.139, 2.629)	1.037 (0.417, 2.581)	0.751 (0.276, 2.046)	0.747 (0.237, 2.356)	0.631 (0.296, 1.346)
VT	0.683 (0.056, 8.385)	0.507 (0.026, 9.934)	1.491 (0.151, 14.776)	1.015 (0.142, 7.255)	0.111 (0.007, 1.676)	0.65 (0.087, 4.882)	6.131 (0, 18198027.834)	0.49 (0.052, 4.645)	0.279 (0.005, 15.597)	0.001 (0, 89.913)	0.999 (0.125, 8.001)
WA	1.007 (0.975, 1.039)	1.015 (0.979, 1.053)	0.99 (0.96, 1.02)	0.98 (0.95, 1.012)	1.009 (0.984, 1.034)	0.993 (0.966, 1.02)	0.955 (0.875, 1.042)	0.994 (0.963, 1.026)	0.987 (0.949, 1.027)	0.964 (0.908, 1.023)	0.994 (0.972, 1.017)
WI	1.21 (0.848, 1.727)	1.077 (0.702, 1.651)	0.912 (0.679, 1.223)	0.931 (0.661, 1.311)	1.028 (0.797, 1.325)	0.864 (0.644, 1.16)	1.26 (0.529, 3.002)	1.017 (0.736, 1.406)	0.934 (0.626, 1.394)	1.045 (0.597, 1.831)	1.277 (0.995, 1.639)
Non-wildfire $PM_{2.5}$											
AZ	0.997 (0.982, 1.011)	0.994 (0.976, 1.011)	0.996 (0.981, 1.012)	0.998 (0.983, 1.012)	0.993 (0.982, 1.005)	0.992 (0.98, 1.005)	0.981 (0.949, 1.014)	0.987 (0.974, 1.001)	0.994 (0.976, 1.012)	1.002 (0.979, 1.025)	0.994 (0.983, 1.004)
CA	1.037 (1.005, 1.071)	1.176 (1.136, 1.217)	1.015 (0.986, 1.043)	1.042 (1.008, 1.078)	1.036 (1.014, 1.059)	1.047 (1.019, 1.076)	0.989 (0.92, 1.062)	1.097 (1.066, 1.129)	1.08 (1.036, 1.126)	0.983 (0.93, 1.04)	1.094 (1.074, 1.115)

CO	0.966 (0.932, 1.001)	1.006 (0.967, 1.047)	0.918 (0.888, 0.95)	0.959 (0.926, 0.993)	0.983 (0.96, 1.006)	0.991 (0.965, 1.017)	0.985 (0.929, 1.044)	0.979 (0.947, 1.012)	0.978 (0.936, 1.021)	0.985 (0.938, 1.033)	0.983 (0.964, 1.002)
DE	0.923 (0.768, 1.111)	1.05 (0.875, 1.261)	0.881 (0.749, 1.038)	1.013 (0.854, 1.202)	0.958 (0.849, 1.081)	0.966 (0.841, 1.11)	1.089 (0.859, 1.38)	0.979 (0.838, 1.143)	1.001 (0.816, 1.229)	1.058 (0.814, 1.375)	0.983 (0.888, 1.088)
GA	1.004 (0.983, 1.025)	1.003 (0.979, 1.028)	1.013 (0.996, 1.031)	1.007 (0.985, 1.029)	1.07 (1.056, 1.083)	1.002 (0.985, 1.019)	1.002 (0.953, 1.053)	0.997 (0.978, 1.017)	1.027 (1.005, 1.049)	1.003 (0.97, 1.038)	0.996 (0.982, 1.011)
IA	1 (0.967, 1.034)	0.996 (0.953, 1.042)	0.986 (0.956, 1.016)	0.987 (0.954, 1.022)	1.034 (1.006, 1.062)	0.965 (0.936, 0.995)	0.993 (0.914, 1.079)	0.998 (0.968, 1.029)	0.982 (0.948, 1.018)	0.994 (0.929, 1.063)	0.989 (0.963, 1.016)
IN	0.952 (0.896, 1.012)	1.002 (0.931, 1.077)	0.996 (0.945, 1.05)	0.991 (0.928, 1.057)	1.079 (1.037, 1.123)	1.01 (0.959, 1.062)	1.016 (0.856, 1.205)	0.994 (0.941, 1.049)	1.021 (0.959, 1.088)	1.003 (0.88, 1.142)	0.969 (0.935, 1.005)
KY	0.99 (0.971, 1.01)	0.986 (0.96, 1.013)	1.016 (0.998, 1.034)	0.981 (0.959, 1.003)	0.991 (0.975, 1.006)	1.005 (0.986, 1.024)	0.947 (0.902, 0.995)	0.984 (0.967, 1.002)	0.982 (0.964, 1)	0.953 (0.919, 0.988)	0.986 (0.971, 1)
MD	0.979 (0.816, 1.174)	0.965 (0.805, 1.156)	0.91 (0.749, 1.105)	1.058 (0.887, 1.263)	1.011 (0.902, 1.134)	1.067 (0.945, 1.206)	0.899 (0.571, 1.416)	0.854 (0.727, 1.002)	1.051 (0.849, 1.302)	1.084 (0.785, 1.497)	0.947 (0.851, 1.053)
MI	0.992 (0.978, 1.006)	0.999 (0.981, 1.018)	0.999 (0.986, 1.013)	0.985 (0.97, 1.001)	0.972 (0.961, 0.982)	0.982 (0.97, 0.995)	1.004 (0.966, 1.044)	0.986 (0.971, 1.001)	0.986 (0.97, 1.002)	0.997 (0.973, 1.022)	1 (0.988, 1.011)
MN	1.016 (0.991, 1.043)	0.998 (0.968, 1.03)	1.008 (0.987, 1.03)	0.987 (0.964, 1.01)	1.007 (0.988, 1.027)	0.995 (0.976, 1.015)	0.992 (0.935, 1.053)	0.999 (0.975, 1.023)	1.002 (0.97, 1.034)	1.003 (0.963, 1.045)	0.996 (0.979, 1.014)
NC	1.007 (0.99, 1.024)	1 (0.98, 1.02)	1.011 (0.996, 1.027)	0.997 (0.98, 1.014)	1.008 (0.996, 1.02)	0.998 (0.983, 1.012)	1.008 (0.963, 1.055)	0.993 (0.976, 1.009)	0.999 (0.981, 1.018)	1.03 (1.001, 1.06)	0.992 (0.98, 1.004)
NE	1.037 (0.964, 1.115)	1.021 (0.938, 1.113)	1.033 (0.97, 1.1)	0.957 (0.893, 1.024)	0.962 (0.913, 1.013)	0.997 (0.941, 1.057)	1 (0.84, 1.19)	1.018 (0.954, 1.087)	0.984 (0.906, 1.068)	1.058 (0.903, 1.238)	0.995 (0.949, 1.043)
NJ	1.013 (0.994, 1.032)	1.001 (0.976, 1.027)	1.003 (0.983, 1.022)	0.998 (0.978, 1.018)	1.011 (0.998, 1.025)	1 (0.983, 1.017)	0.986 (0.941, 1.033)	0.999 (0.978, 1.021)	1.012 (0.987, 1.036)	0.996 (0.969, 1.024)	1.014 (0.997, 1.032)
NY	1.018 (1.009, 1.028)	1.013 (1, 1.026)	1.013 (1.004, 1.022)	1.002 (0.992, 1.012)	1.013 (1.006, 1.02)	1.013 (1.005, 1.022)	0.987 (0.964, 1.011)	1.013 (1.002, 1.024)	1.013 (1.001, 1.025)	1 (0.987, 1.014)	1.013 (1.004, 1.021)

OR	1.017 (0.996, 1.038)	0.997 (0.972, 1.023)	1.006 (0.987, 1.025)	0.991 (0.971, 1.012)	1.006 (0.989, 1.023)	0.992 (0.975, 1.011)	1.002 (0.947, 1.061)	1.016 (0.995, 1.039)	1.019 (0.992, 1.046)	1.001 (0.965, 1.039)	0.998 (0.982, 1.013)
RI	1.023 (0.966, 1.084)	1.001 (0.924, 1.083)	1.078 (1.014, 1.145)	1.003 (0.941, 1.068)	0.996 (0.954, 1.039)	0.987 (0.932, 1.046)	1.022 (0.885, 1.181)	1.024 (0.956, 1.097)	1.052 (0.977, 1.132)	0.983 (0.897, 1.079)	1.039 (0.984, 1.096)
VT	0.976 (0.737, 1.291)	1.045 (0.737, 1.482)	1.006 (0.77, 1.316)	0.987 (0.746, 1.305)	1.04 (0.829, 1.305)	0.937 (0.725, 1.212)	1.112 (0.47, 2.633)	1.042 (0.78, 1.393)	1.147 (0.797, 1.652)	0.782 (0.411, 1.488)	0.975 (0.796, 1.194)
WA	1.005 (0.989, 1.021)	1.001 (0.982, 1.02)	1.001 (0.987, 1.015)	1.009 (0.993, 1.025)	0.995 (0.982, 1.007)	1.008 (0.995, 1.022)	1.001 (0.962, 1.042)	0.997 (0.981, 1.013)	0.994 (0.975, 1.014)	0.99 (0.964, 1.017)	1 (0.989, 1.012)
WI	0.998 (0.967, 1.03)	1.006 (0.968, 1.046)	1 (0.973, 1.029)	0.976 (0.945, 1.007)	1.006 (0.983, 1.03)	0.976 (0.95, 1.003)	0.979 (0.902, 1.061)	1.003 (0.972, 1.035)	1.003 (0.965, 1.043)	0.997 (0.946, 1.051)	0.995 (0.972, 1.019)

Abbreviations: AZ, Arizona; CA, California; CO, Colorado; DE, Delaware; GA, Georgia; IA, Iowa; IN, Indiana; KY, Kentucky; MD, Maryland; MI, Michigan; MN, Minnesota; NC, North Carolina; NE, Nebraska; NJ, New Jersey; NY, New York; OR, Oregon; RI, Rhode Island; VT, Vermont; WA, Washington; WI, Wisconsin.

Note: The model was adjusted for calendar year, percentage of White, percentage of Black, percentage of the population who graduated high school, median household income, townsend deprivation index, mean temperature of summer and winter, and standard deviation of summer and winter temperature. Relative risks were reported per 1 μg/m³ increase in 2-year average $PM_{2.5}$ concentrations. All statistical tests were two-sided.

Supplementary Table 11. Subgroup analysis by age.

Outcome	Wildfire-specific $PM_{2.5}$				Non-wildfire $PM_{2.5}$		
	Under 65 years old	65 years old and above	*P*-value[a]		Under 65 years old	65 years old and above	*P*-value[b]
**CVD**							
Ischemic heart disease	1.129 (1.112, 1.146)	1.130 (1.118, 1.142)	0.876		1.067 (1.061, 1.072)	1.064 (1.059, 1.068)	0.247
Cerebrovascular disease	1.141 (1.120, 1.162)	1.127 (1.113, 1.142)	0.142		1.081 (1.073, 1.089)	1.067 (1.061, 1.072)	3.23E-05
Heart failure	1.144 (1.127, 1.162)	1.086 (1.076, 1.096)	1.08E-16		1.079 (1.072, 1.085)	1.046 (1.042, 1.050)	8.14E-34
Arrhythmia	1.141 (1.121, 1.162)	1.110 (1.099, 1.121)	0.000143		1.061 (1.054, 1.068)	1.049 (1.044, 1.053)	2.34E-05
Hypertension	1.121 (1.110, 1.131)	1.130 (1.121, 1.139)	0.054		1.084 (1.081, 1.088)	1.085 (1.081, 1.088)	0.907
Other cardiovascular diseases	1.127 (1.115, 1.140)	1.111 (1.100, 1.122)	0.005		1.072 (1.067, 1.076)	1.056 (1.052, 1.060)	6.48E-13
**RD**							
Acute respiratory infections [c]	1.123 (1.092, 1.156)	1.089 (1.052, 1.126)	0.045		1.050 (1.039, 1.061)	1.062 (1.049, 1.075)	0.053
Pneumonia	1.105 (1.090, 1.120)	1.111 (1.099, 1.123)	0.356		1.056 (1.051, 1.062)	1.056 (1.052, 1.061)	0.980
COPD	1.103 (1.083, 1.122)	1.119 (1.105, 1.132)	0.057		1.047 (1.040, 1.054)	1.047 (1.042, 1.052)	0.948
Asthma [c]	1.165 (1.127, 1.205)	1.158 (1.138, 1.178)	0.645		1.067 (1.055, 1.079)	1.058 (1.051, 1.064)	0.052
Other respiratory diseases	1.116 (1.106, 1.126)	1.113 (1.104, 1.121)	0.474		1.071 (1.067, 1.075)	1.062 (1.059, 1.066)	1.19E-05

Abbreviations: CVD, cardiovascular diseases; RD, respiratory diseases; COPD, chronic obstructive pulmonary disease.

The model was adjusted for calendar year, percentage of White, percentage of Black, percentage of the population who graduated high school, median household income, townsend deprivation index, mean temperature of summer and winter, standard deviation of summer and winter. Relative risks were reported per 1 μg/m³ increase in 2-year average $PM_{2.5}$ concentrations. All statistical tests were two-sided.

[a] The comparison of wildfire-specific $PM_{2.5}$ effect values between subgroups. *P*-value was calculated by Z-test.

[b] The comparison of non-wildfire $PM_{2.5}$ effect values between subgroups. *P*-value was calculated by Z-test.

[c] The group was divided into under 18 years old vs. 18 years old and above.

Supplementary Table 12. Subgroup analysis by sex.

Outcome	Wildfire-specific $PM_{2.5}$				Non-wildfire $PM_{2.5}$		
	Female	Male	*P*-value[a]		Female	Male	*P*-value[b]
**CVD**							
Ischemic heart disease	1.132 (1.116, 1.148)	1.127 (1.114, 1.139)	0.441		1.067 (1.062, 1.073)	1.063 (1.059, 1.068)	0.134
Cerebrovascular disease	1.131 (1.115, 1.148)	1.130 (1.114, 1.147)	0.868		1.070 (1.064, 1.076)	1.072 (1.066, 1.079)	0.422
Heart failure	1.093 (1.080, 1.105)	1.106 (1.094, 1.118)	0.032		1.052 (1.047, 1.057)	1.057 (1.053, 1.062)	0.024
Arrhythmia	1.122 (1.108, 1.137)	1.114 (1.100, 1.127)	0.223		1.053 (1.047, 1.058)	1.051 (1.046, 1.056)	0.453
Hypertension	1.121 (1.111, 1.131)	1.124 (1.114, 1.134)	0.510		1.082 (1.078, 1.086)	1.088 (1.084, 1.092)	0.002
Other cardiovascular diseases	1.116 (1.105, 1.128)	1.119 (1.107, 1.130)	0.727		1.062 (1.057, 1.066)	1.063 (1.059, 1.067)	0.530
**RD**							
Acute respiratory infections	1.101 (1.068, 1.136)	1.119 (1.085, 1.154)	0.305		1.053 (1.041, 1.066)	1.054 (1.042, 1.066)	0.914
Pneumonia	1.108 (1.095, 1.121)	1.109 (1.096, 1.123)	0.822		1.054 (1.049, 1.059)	1.058 (1.053, 1.063)	0.138
COPD	1.117 (1.101, 1.132)	1.107 (1.091, 1.124)	0.235		1.046 (1.041, 1.052)	1.047 (1.041, 1.053)	0.811
Asthma	1.156 (1.134, 1.178)	1.165 (1.135, 1.197)	0.479		1.060 (1.053, 1.067)	1.059 (1.049, 1.068)	0.748
Other respiratory diseases	1.115 (1.106, 1.125)	1.113 (1.104, 1.122)	0.579		1.064 (1.061, 1.068)	1.068 (1.064, 1.071)	0.101

Abbreviations: CVD, cardiovascular diseases; RD, respiratory diseases; COPD, chronic obstructive pulmonary disease.

The model was adjusted for calendar year, percentage of White, percentage of Black, percentage of the population who graduated high school, median household income, townsend deprivation index, mean temperature of summer and winter, standard deviation of summer and winter. Relative risks were reported per 1 $\mu g/m^3$ increase in 2-year average $PM_{2.5}$ concentrations. All statistical tests were two-sided.

[a] The comparison of wildfire-specific $PM_{2.5}$ effect values between subgroups. *P*-value was calculated by Z-test.

[b] The comparison of non-wildfire $PM_{2.5}$ effect values between subgroups. *P*-value was calculated by Z-test.

Supplementary Table 13. Subgroup analysis by race/ethnicity.

Outcome	Wildfire-specific $PM_{2.5}$					Non-wildfire $PM_{2.5}$				
	White	Black	Hispanic	Other	*P*-value	White	Black	Hispanic	Other	*P*-value
**CVD**										
Ischemic heart disease	1.095 (1.084, 1.106)	1.061 (1.017, 1.106)	1.199 (1.150, 1.249)	1.309 (1.258, 1.363)	9.16E-41	1.070 (1.066, 1.075)	1.112 (1.098, 1.126)	1.107 (1.091, 1.124)	1.137 (1.122, 1.153)	2.41E-46
Cerebrovascular disease	1.106 (1.093, 1.120)	1.077 (1.034, 1.123)	1.138 (1.089, 1.190)	1.289 (1.232, 1.349)	4.10E-20	1.073 (1.067, 1.079)	1.117 (1.102, 1.132)	1.105 (1.085, 1.125)	1.165 (1.145, 1.185)	3.60E-46
Heart failure	1.073 (1.063, 1.082)	1.065 (1.033, 1.097)	1.123 (1.080, 1.167)	1.169 (1.125, 1.216)	2.47E-10	1.053 (1.049, 1.057)	1.100 (1.089, 1.110)	1.063 (1.048, 1.079)	1.128 (1.112, 1.145)	6.32E-61
Arrhythmia	1.098 (1.087, 1.109)	1.090 (1.040, 1.143)	1.156 (1.101, 1.213)	1.212 (1.156, 1.272)	6.97E-09	1.063 (1.059, 1.068)	1.092 (1.077, 1.108)	1.079 (1.060, 1.098)	1.119 (1.101, 1.138)	9.06E-21
Hypertension	1.076 (1.068, 1.084)	1.059 (1.036, 1.083)	1.195 (1.165, 1.227)	1.308 (1.271, 1.346)	1.14E-100	1.079 (1.076, 1.083)	1.119 (1.112, 1.127)	1.102 (1.091, 1.112)	1.153 (1.141, 1.165)	5.49E-96
Other cardiovascular diseases	1.093 (1.084, 1.103)	1.075 (1.043, 1.107)	1.160 (1.123, 1.199)	1.238 (1.195, 1.283)	1.11E-25	1.064 (1.061, 1.068)	1.102 (1.092, 1.112)	1.105 (1.091, 1.119)	1.136 (1.121, 1.151)	6.59E-64
**RD**										
Acute respiratory infections	1.061 (1.031, 1.092)	1.100 (1.014, 1.193)	1.173 (1.108, 1.241)	1.253 (1.157, 1.356)	1.35E-09	1.046 (1.034, 1.058)	1.109 (1.083, 1.137)	1.086 (1.064, 1.110)	1.109 (1.078, 1.140)	2.91E-14
Pneumonia	1.088 (1.077, 1.098)	1.062 (1.022, 1.103)	1.149 (1.108, 1.191)	1.237 (1.191, 1.285)	2.22E-22	1.054 (1.049, 1.058)	1.106 (1.093, 1.119)	1.094 (1.079, 1.109)	1.133 (1.117, 1.150)	2.58E-66
COPD	1.087 (1.075, 1.099)	1.102 (1.050, 1.157)	1.190 (1.115, 1.270)	1.240 (1.171, 1.314)	1.00E-11	1.042 (1.037, 1.047)	1.125 (1.108, 1.142)	1.066 (1.042, 1.091)	1.121 (1.098, 1.146)	2.69E-57
Asthma	1.120 (1.098, 1.143)	1.143 (1.091, 1.199)	1.243 (1.184, 1.304)	1.280 (1.204, 1.361)	6.23E-13	1.059 (1.051, 1.067)	1.088 (1.074, 1.102)	1.084 (1.067, 1.101)	1.108 (1.086, 1.130)	2.54E-12
Other respiratory diseases	1.089 (1.082, 1.096)	1.089 (1.061, 1.118)	1.143 (1.115, 1.173)	1.264 (1.230, 1.298)	4.68E-55	1.066 (1.062, 1.069)	1.119 (1.109, 1.129)	1.108 (1.096, 1.119)	1.154 (1.141, 1.166)	4.28E-133

Abbreviations: CVD, cardiovascular diseases; RD, respiratory diseases; COPD, chronic obstructive pulmonary disease.

The model was adjusted for calendar year, percentage of White, percentage of Black, percentage of the population who graduated high school, median household income, mean temperature of summer and winter, standard deviation of summer and winter. Relative risks were reported per 1 $\mu g/m^3$ increase in 2-year average $PM_{2.5}$ concentrations. All statistical tests were two-sided.

[a] The comparison of wildfire-specific $PM_{2.5}$ effect values between subgroups. *P*-value was calculated by Wald test.

[b] The comparison of non-wildfire $PM_{2.5}$ effect values between subgroups. *P*-value was calculated by Wald test.

Supplementary Table 14. Subgroup analysis by patient location.

Outcome	Wildfire-specific $PM_{2.5}$				Non-wildfire $PM_{2.5}$			
	Metropolitan	Micropolitan	Non-CBSA	*P*-value[a]	Metropolitan	Micropolitan	Non-CBSA	*P*-value[b]
**CVD**								
Ischemic heart disease	1.144 (1.133, 1.156)	1.034 (1.008, 1.060)	1.017 (0.986, 1.050)	8.84E-44	1.069 (1.065, 1.073)	1.014 (1.004, 1.024)	0.994 (0.981, 1.007)	8.11E-85
Cerebrovascular disease	1.146 (1.133, 1.160)	1.046 (1.014, 1.079)	1.017 (0.978, 1.057)	6.72E-27	1.074 (1.069, 1.079)	1.006 (0.992, 1.020)	1.015 (0.996, 1.033)	4.37E-48
Heart failure	1.106 (1.097, 1.116)	1.029 (1.006, 1.052)	0.993 (0.963, 1.023)	3.99E-34	1.054 (1.050, 1.057)	1.010 (1.000, 1.020)	1.007 (0.994, 1.020)	4.95E-45
Arrhythmia	1.129 (1.118, 1.140)	1.032 (1.006, 1.059)	1.033 (1.000, 1.067)	3.13E-28	1.054 (1.050, 1.058)	1.001 (0.990, 1.012)	1.005 (0.991, 1.019)	1.47E-48
Hypertension	1.131 (1.123, 1.138)	1.034 (1.016, 1.053)	1.031 (1.007, 1.056)	6.50E-56	1.089 (1.086, 1.092)	1.002 (0.994, 1.011)	1.002 (0.991, 1.013)	3.92E-235
Other cardiovascular diseases	1.128 (1.119, 1.138)	1.043 (1.021, 1.066)	1.026 (0.998, 1.055)	1.63E-35	1.064 (1.061, 1.067)	1.006 (0.996, 1.016)	1.007 (0.995, 1.021)	1.33E-75
**RD**								
Acute respiratory infections	1.121 (1.094, 1.149)	1.061 (0.992, 1.134)	1.040 (0.954, 1.133)	0.008	1.056 (1.047, 1.065)	0.988 (0.961, 1.016)	0.976 (0.942, 1.012)	1.61E-16
Pneumonia	1.118 (1.108, 1.129)	1.052 (1.027, 1.077)	1.015 (0.985, 1.045)	4.19E-25	1.059 (1.055, 1.063)	0.995 (0.985, 1.005)	0.991 (0.978, 1.004)	1.24E-93
COPD	1.123 (1.110, 1.137)	1.047 (1.019, 1.075)	1.050 (1.015, 1.086)	2.72E-15	1.054 (1.049, 1.058)	1.005 (0.993, 1.016)	0.996 (0.982, 1.010)	2.06E-47
Asthma	1.175 (1.155, 1.195)	1.041 (0.992, 1.092)	1.026 (0.959, 1.098)	5.84E-16	1.060 (1.054, 1.066)	1.023 (1.003, 1.043)	1.010 (0.983, 1.037)	4.66E-11
Other respiratory diseases	1.120 (1.113, 1.127)	1.074 (1.056, 1.092)	1.040 (1.016, 1.065)	3.93E-24	1.069 (1.066, 1.072)	0.996 (0.988, 1.005)	0.999 (0.988, 1.010)	1.70E-160

Abbreviations: CVD, cardiovascular diseases; RD, respiratory diseases; COPD, chronic obstructive pulmonary disease.

The model was adjusted for calendar year, percentage of White, percentage of Black, percentage of the population who graduated high school, median household income, mean temperature of summer and winter, standard deviation of summer and winter. Relative risks were reported per 1 $\mu g/m^3$ increase in 2-year average $PM_{2.5}$ concentrations. All statistical tests were two-sided.

[a] The comparison of wildfire-specific $PM_{2.5}$ effect values between subgroups. *P*-value was calculated by Wald test.

[b] The comparison of non-wildfire $PM_{2.5}$ effect values between subgroups. *P*-value was calculated by Wald test.

Supplementary Table 15. Subgroup analysis by ICD-9-CM diagnoses of obesity.

Outcome	Wildfire-specific $PM_{2.5}$				Non-wildfire $PM_{2.5}$		
	Obesity	Non-obesity	*P*-value[a]		Obesity	Non-obesity	*P*-value[b]
**CVD**							
Ischemic heart disease	1.184 (1.152, 1.216)	1.123 (1.112, 1.133)	1.92E-07		1.081 (1.069, 1.093)	1.063 (1.059, 1.067)	5.31E-05
Cerebrovascular disease	1.196 (1.148, 1.247)	1.127 (1.115, 1.139)	7.27E-05		1.101 (1.082, 1.120)	1.069 (1.065, 1.074)	4.71E-06
Heart failure	1.121 (1.095, 1.148)	1.097 (1.088, 1.106)	0.013		1.074 (1.063, 1.085)	1.053 (1.049, 1.056)	2.31E-07
Arrhythmia	1.161 (1.129, 1.195)	1.113 (1.102, 1.123)	4.51E-05		1.061 (1.049, 1.074)	1.051 (1.047, 1.055)	0.026
Hypertension	1.102 (1.082, 1.121)	1.125 (1.117, 1.132)	0.0015		1.090 (1.082, 1.098)	1.084 (1.081, 1.087)	0.046
Other cardiovascular diseases	1.150 (1.123, 1.177)	1.114 (1.105, 1.123)	0.000316		1.073 (1.062, 1.084)	1.061 (1.058, 1.065)	0.003
**RD**							
Acute respiratory infections	1.125 (1.016, 1.246)	1.110 (1.086, 1.135)	0.720		1.071 (1.031, 1.113)	1.053 (1.044, 1.062)	0.222
Pneumonia	1.146 (1.107, 1.185)	1.106 (1.097, 1.116)	0.005		1.068 (1.054, 1.083)	1.055 (1.052, 1.059)	0.014
COPD	1.151 (1.115, 1.189)	1.108 (1.096, 1.120)	0.001		1.065 (1.051, 1.079)	1.045 (1.040, 1.049)	6.59E-05
Asthma	1.158 (1.108, 1.211)	1.159 (1.140, 1.179)	0.950		1.059 (1.043, 1.076)	1.060 (1.054, 1.066)	0.952
Other respiratory diseases	1.147 (1.124, 1.170)	1.111 (1.104, 1.118)	1.27E-05		1.074 (1.064, 1.084)	1.065 (1.062, 1.068)	0.020

Abbreviations: CVD, cardiovascular diseases; RD, respiratory diseases; COPD, chronic obstructive pulmonary disease.
The model was adjusted for calendar year, percentage of White, percentage of Black, percentage of the population who graduated high school, median household income, townsend deprivation index, mean temperature of summer and winter, standard deviation of summer and winter. Relative risks were reported per 1 μg/m³ increase in 2-year average $PM_{2.5}$ concentrations. All statistical tests were two-sided.
[a] The comparison of wildfire-specific $PM_{2.5}$ effect values between subgroups. *P*-value was calculated by Z-test.
[b] The comparison of non-wildfire $PM_{2.5}$ effect values between subgroups. *P*-value was calculated by Z-test.

Supplementary Table 16. Subgroup analysis by comorbidity status of diabetes.

Outcome	Wildfire-specific $PM_{2.5}$				Non-wildfire $PM_{2.5}$		
	Diabetes	Non-diabetes	*P*-value[a]		Diabetes	Non-diabetes	*P*-value[b]
**CVD**							
Ischemic heart disease	1.144 (1.125, 1.162)	1.122 (1.110, 1.134)	0.004		1.073 (1.067, 1.080)	1.061 (1.057, 1.065)	3.93E-06
Cerebrovascular disease	1.151 (1.128, 1.175)	1.123 (1.110, 1.137)	0.003		1.085 (1.076, 1.094)	1.066 (1.061, 1.071)	1.01E-07
Heart failure	1.106 (1.091, 1.121)	1.096 (1.085, 1.106)	0.109		1.059 (1.054, 1.065)	1.052 (1.048, 1.056)	0.004
Arrhythmia	1.114 (1.093, 1.135)	1.118 (1.107, 1.129)	0.582		1.053 (1.045, 1.061)	1.051 (1.047, 1.055)	0.498
Hypertension	1.116 (1.103, 1.129)	1.119 (1.111, 1.127)	0.600		1.100 (1.094, 1.105)	1.078 (1.075, 1.081)	6.65E-25
Other cardiovascular diseases	1.110 (1.092, 1.127)	1.119 (1.110, 1.128)	0.178		1.062 (1.055, 1.068)	1.062 (1.059, 1.066)	0.776
**RD**							
Acute respiratory infections	1.068 (0.993, 1.150)	1.114 (1.089, 1.140)	0.120		1.059 (1.031, 1.089)	1.054 (1.045, 1.062)	0.595
Pneumonia	1.123 (1.103, 1.144)	1.104 (1.094, 1.115)	0.020		1.066 (1.058, 1.073)	1.053 (1.049, 1.057)	5.07E-05
COPD	1.111 (1.087, 1.134)	1.113 (1.100, 1.126)	0.821		1.048 (1.040, 1.057)	1.046 (1.041, 1.051)	0.437
Asthma	1.147 (1.095, 1.202)	1.161 (1.142, 1.180)	0.505		1.039 (1.024, 1.055)	1.062 (1.056, 1.069)	8.69E-05
Other respiratory diseases	1.134 (1.119, 1.148)	1.109 (1.102, 1.116)	1.74E-05		1.073 (1.067, 1.079)	1.064 (1.061, 1.067)	0.000152

Abbreviations: CVD, cardiovascular diseases; RD, respiratory diseases; COPD, chronic obstructive pulmonary disease.

The model was adjusted for calendar year, percentage of White, percentage of Black, percentage of the population who graduated high school, median household income, townsend deprivation index, mean temperature of summer and winter, standard deviation of summer and winter. Relative risks were reported per 1 $\mu g/m^3$ increase in 2-year average $PM_{2.5}$ concentrations. All statistical tests were two-sided.

[a] The comparison of wildfire-specific $PM_{2.5}$ effect values between subgroups. *P*-value was calculated by Z-test.

[b] The comparison of non-wildfire $PM_{2.5}$ effect values between subgroups. *P*-value was calculated by Z-test.

Supplementary Table 17. Subgroup analysis by severity of the disease.

Outcome	Wildfire-specific $PM_{2.5}$				Non-wildfire $PM_{2.5}$		
	Died during hospitalization	Not died during hospitalization	*P*-value[a]		Died during hospitalization	Not died during hospitalization	*P*-value[b]
**CVD**							
Ischemic heart disease	1.096 (1.051, 1.143)	1.130 (1.120, 1.140)	0.045		1.080 (1.061, 1.099)	1.064 (1.060, 1.068)	0.016
Cerebrovascular disease	1.158 (1.108, 1.210)	1.129 (1.117, 1.141)	0.115		1.082 (1.062, 1.102)	1.070 (1.066, 1.075)	0.113
Heart failure	1.112 (1.064, 1.162)	1.099 (1.090, 1.107)	0.445		1.054 (1.035, 1.074)	1.054 (1.051, 1.058)	0.990
Arrhythmia	1.099 (1.033, 1.168)	1.117 (1.107, 1.127)	0.443		1.047 (1.023, 1.071)	1.051 (1.048, 1.055)	0.618
Hypertension	1.254 (1.175, 1.339)	1.120 (1.114, 1.127)	1.04E-06		1.114 (1.081, 1.149)	1.084 (1.082, 1.087)	0.011
Other cardiovascular diseases	1.110 (1.065, 1.157)	1.117 (1.109, 1.126)	0.662		1.061 (1.042, 1.080)	1.062 (1.059, 1.065)	0.856
**RD**							
Acute respiratory infections	1.301 (0.703, 2.407)	1.110 (1.086, 1.134)	0.463		1.148 (0.892, 1.477)	1.053 (1.045, 1.062)	0.335
Pneumonia	1.094 (1.053, 1.137)	1.109 (1.099, 1.119)	0.344		1.062 (1.046, 1.078)	1.055 (1.052, 1.059)	0.252
COPD	1.087 (0.996, 1.185)	1.112 (1.101, 1.124)	0.449		1.038 (1.004, 1.073)	1.046 (1.042, 1.050)	0.487
Asthma	1.098 (0.788, 1.529)	1.159 (1.142, 1.177)	0.643		1.003 (0.896, 1.122)	1.060 (1.054, 1.065)	0.164
Other respiratory diseases	1.120 (1.099, 1.142)	1.113 (1.106, 1.120)	0.367		1.075 (1.066, 1.084)	1.065 (1.062, 1.068)	0.002

Abbreviations: CVD, cardiovascular diseases; RD, respiratory diseases; COPD, chronic obstructive pulmonary disease.

The model was adjusted for calendar year, percentage of White, percentage of Black, percentage of the population who graduated high school, median household income, townsend deprivation index, mean temperature of summer and winter, standard deviation of summer and winter. Relative risks were reported per 1 $\mu g/m^3$ increase in 2-year average $PM_{2.5}$ concentrations. All statistical tests were two-sided.

[a] The comparison of wildfire-specific $PM_{2.5}$ effect values between subgroups. *P*-value was calculated by Z-test.

[b] The comparison of non-wildfire $PM_{2.5}$ effect values between subgroups. *P*-value was calculated by Z-test.

Supplementary Table 18. Subgroup analysis by percentage of the population who graduated high school.

Outcome	Wildfire-specific $PM_{2.5}$				Non-wildfire $PM_{2.5}$		
	Percentage of the population who graduated high school ≤ 25th	Percentage of the population who graduated high school > 25th	*P*-value[a]		Percentage of the population who graduated high school ≤ 25th	Percentage of the population who graduated high school > 25th	*P*-value[b]
**CVD**							
Ischemic heart disease	1.181 (1.154, 1.209)	1.126 (1.115, 1.137)	6.32E-08		1.107 (1.099, 1.115)	1.050 (1.046, 1.054)	1.09E-73
Cerebrovascular disease	1.205 (1.172, 1.238)	1.125 (1.113, 1.138)	5.46E-11		1.125 (1.115, 1.136)	1.052 (1.047, 1.057)	1.69E-75
Heart failure	1.179 (1.154, 1.204)	1.092 (1.083, 1.102)	1.96E-21		1.098 (1.090, 1.106)	1.036 (1.032, 1.040)	2.13E-98
Arrhythmia	1.187 (1.161, 1.214)	1.118 (1.107, 1.129)	3.73E-12		1.096 (1.088, 1.104)	1.037 (1.033, 1.041)	4.78E-75
Hypertension	1.245 (1.225, 1.266)	1.112 (1.105, 1.120)	1.77E-71		1.130 (1.124, 1.136)	1.062 (1.059, 1.066)	2.5E-177
Other cardiovascular diseases	1.169 (1.147, 1.192)	1.114 (1.105, 1.123)	3.74E-11		1.119 (1.112, 1.126)	1.044 (1.040, 1.047)	7.6E-167
**RD**							
Acute respiratory infections	1.125 (1.064, 1.189)	1.111 (1.084, 1.138)	0.561		1.072 (1.054, 1.091)	1.043 (1.033, 1.053)	6.13E-05
Pneumonia	1.159 (1.134, 1.185)	1.110 (1.100, 1.121)	3.16E-07		1.095 (1.087, 1.103)	1.040 (1.036, 1.044)	7.02E-70
COPD	1.193 (1.162, 1.225)	1.108 (1.096, 1.121)	1.72E-13		1.087 (1.078, 1.097)	1.033 (1.028, 1.037)	6.5E-49
Asthma	1.213 (1.163, 1.265)	1.160 (1.140, 1.179)	0.005		1.094 (1.081, 1.108)	1.049 (1.043, 1.056)	1.26E-18
Other respiratory diseases	1.192 (1.175, 1.211)	1.105 (1.098, 1.112)	1.7E-39		1.124 (1.118, 1.130)	1.044 (1.040, 1.047)	7.8E-258

Abbreviations: CVD, cardiovascular diseases; RD, respiratory diseases; COPD, chronic obstructive pulmonary disease.

The model was adjusted for calendar year, percentage of White, percentage of Black, median household income, townsend deprivation index, mean temperature of summer and winter, standard deviation of summer and winter. Relative risks were reported per 1 μg/m³ increase in 2-year average $PM_{2.5}$ concentrations. All statistical tests were two-sided.

[a] The comparison of wildfire-specific $PM_{2.5}$ effect values between subgroups. *P*-value was calculated by Z-test.

[b] The comparison of non-wildfire $PM_{2.5}$ effect values between subgroups. *P*-value was calculated by Z-test.

Supplementary Table 19. Subgroup analysis by townsend deprivation index.

Outcome	Wildfire-specific $PM_{2.5}$				Non-wildfire $PM_{2.5}$		
	Townsend deprivation index ≤ 75th	Townsend deprivation index > 75th	*P*-value[a]		Townsend deprivation index ≤ 75th	Townsend deprivation index > 75th	*P*-value[b]
**CVD**							
Ischemic heart disease	1.104 (1.093, 1.115)	1.226 (1.199, 1.253)	2E-35		1.042 (1.038, 1.046)	1.108 (1.101, 1.116)	5E-109
Cerebrovascular disease	1.105 (1.092, 1.118)	1.219 (1.187, 1.252)	5.01E-22		1.047 (1.042, 1.052)	1.123 (1.114, 1.133)	2.38E-87
Heart failure	1.078 (1.069, 1.088)	1.180 (1.155, 1.205)	2.83E-29		1.034 (1.030, 1.038)	1.100 (1.093, 1.107)	4.9E-122
Arrhythmia	1.085 (1.074, 1.096)	1.235 (1.209, 1.261)	8.15E-56		1.031 (1.027, 1.035)	1.097 (1.089, 1.104)	1.8E-103
Hypertension	1.103 (1.096, 1.110)	1.240 (1.217, 1.264)	2.43E-60		1.061 (1.058, 1.064)	1.137 (1.131, 1.143)	1E-228
Other cardiovascular diseases	1.090 (1.082, 1.099)	1.229 (1.206, 1.252)	4.13E-60		1.039 (1.035, 1.042)	1.117 (1.110, 1.124)	4E-194
**RD**							
Acute respiratory infections	1.089 (1.064, 1.115)	1.223 (1.141, 1.310)	6.82E-06		1.040 (1.030, 1.049)	1.072 (1.053, 1.092)	1.56E-05
Pneumonia	1.078 (1.068, 1.088)	1.198 (1.173, 1.224)	7.8E-38		1.030 (1.026, 1.034)	1.103 (1.096, 1.111)	1.1E-131
COPD	1.080 (1.068, 1.093)	1.210 (1.181, 1.239)	2.17E-33		1.020 (1.015, 1.024)	1.103 (1.094, 1.112)	8E-126
Asthma	1.150 (1.131, 1.169)	1.163 (1.088, 1.243)	0.635		1.059 (1.053, 1.065)	1.055 (1.042, 1.069)	0.481
Other respiratory diseases	1.090 (1.083, 1.097)	1.214 (1.197, 1.231)	8.29E-86		1.035 (1.032, 1.038)	1.131 (1.126, 1.137)	1E-384

Abbreviations: CVD, cardiovascular diseases; RD, respiratory diseases; COPD, chronic obstructive pulmonary disease.

The model was adjusted for calendar year, percentage of White, percentage of Black, percentage of the population who graduated high school, median household income, mean temperature of summer and winter, standard deviation of summer and winter. Relative risks were reported per 1 $\mu g/m^3$ increase in 2-year average $PM_{2.5}$ concentrations. All statistical tests were two-sided.

[a] The comparison of wildfire-specific $PM_{2.5}$ effect values between subgroups. *P*-value was calculated by Z-test.

[b] The comparison of non-wildfire $PM_{2.5}$ effect values between subgroups. *P*-value was calculated by Z-test.

Supplementary Table 20. Study periods of each state.

State	Abbreviation	Study periods
Arizona	AZ	2006-2019
California	CA	2018-2019
Colorado	CO	2014-2019
Delaware	DE	2016-2019
Georgia	GA	2010-2019
Iowa	IA	2006-2019
Indiana	IN	2017-2019
Kentucky	KY	2006-2019
Maryland	MD	2006-2019
Michigan	MI	2006-2019
Minnesota	MN	2010-2019
North Carolina	NC	2006-2019
Nebraska	NE	2014-2019
New Jersey	NJ	2006-2019
New York	NY	2006-2019
Oregon	OR	2006-2019
Rhode Island	RI	2006-2019
Vermont	VT	2017-2019
Washington	WA	2006-2019
Wisconsin	WI	2012-2019

Supplementary Table 21. The ICD codes used for the disease identification in this study.

Types of diseases	ICD-9 code	ICD-10 code
Cardiovascular diseases		
Ischemic heart disease	410-414	I20-I25
Cerebrovascular disease	430-438	I60-I69
Heart failure	398.91, 402.01, 402.11, 402.91, 404.01, 404.03, 404.11, 404.13, 404.91, 404.93, 428	I50
Arrhythmia	426, 427, 785.0	I44, I45, I47-I49, R00.0, R00.1, R00.8, R94.31, T82.1, T82.11, T82.110, T82.111, T82.118, T82.119, Z45.0, Z45.01, Z45.010, Z45.018, Z45.02, Z45.09, Z95.0
Hypertension	401-405	I10-I15
Other cardiovascular diseases	All other codes within the range 390-459	All other codes within the range I00-I99
Respiratory diseases		
Acute respiratory infections	460-466	J00-J06, J20-J22
Pneumonia	480-486	J12-J18
COPD	491, 492, 496	J41-J44
Asthma	493	J45, J46
Other respiratory diseases	All other codes within the range 460-519	All other codes within the range J00-J99
Obesity	2780, 6491	E66, O99.21
Diabetes	6480, 249, 250	E08-E13

Abbreviations: COPD, chronic obstructive pulmonary disease.
