# Supplementary material for: Cardiopulmonary hospitalization risks from wildfire-specific and non-wildfire PM2.5 in 20 US states
Source: Nat Commun. 2026 Sep 17;17:9349. doi: 10.1038/s41467-026-76974-7 (PMC13586224; doi:10.1038/s41467-026-76974-7)
Supplement: Supplementary file 2 [file 41467_2026_76974_MOESM2_ESM.pdf]

nature portfolio

Peer Review File

Cardiopulmonary hospitalization risks from wildfire and non-wildfire PM2.5 in 20 US states

Corresponding Author: Dr Min Zhang

This file contains all reviewer reports in order by version, followed by all author rebuttals in order by version.

Version 0:

Reviewer comments:

Reviewer #1

(Remarks to the Author)
This study compared the associations between long-term exposure to PM2.5 derived from wildfires versus non-wildfire sources on cardiopulmonary hospitalizations using a dataset of over 89 million hospitalization records across 20 US states (2006–2019). The authors employed a self-controlled study design to estimate the hospitalization risks associated with 2-year moving average exposures. The main finding is that for each 1 µg/m3 increase, the effects of wildfire-specific PM2.5 were significantly stronger than those of non-wildfire PM2.5 across all examined cardiopulmonary outcomes. While the large dataset and examination of multiple cause-specific cardiopulmonary outcomes are strengths, I have several concerns regarding the methodology and interpretation of the comparative toxicity findings.
Major:
(1) The self-controlled design, as an extension of the traditional case-crossover design, was not sufficiently justified in the manuscript. The authors should provide methodological justification or supporting references, and examine how sensitive the results are to the choice of control years. A comparison with estimates from a more standard Cox or Poisson regression would help assess the robustness of the findings.
(2) The study used 2-year averages of wildfire and non-wildfire PM2.5 with substantially overlapped exposure windows for the case year and adjacent control years. As a result, the within-person variation in exposures can be very limited, especially for wildfire-specific PM2.5. This can lead to unstable effect estimates and stronger influence of measurement error.
(3) Please justify the choice of using 2-year moving averages to represent long-term exposure and conduct sensitivity analyses using alternative averaging periods, such as 1-year and 3-year averages, to demonstrate that the findings are robust to the definition of the long-term exposure metric.
(4) The manuscript overstates the confounding control by the self-controlled design. While time-invariant characteristics are addressed, several important factors, such as chronic disease status, medication use (which probably change following cardiopulmonary hospitalizations), and smoking behavior, can change substantially over the 3-year case-control window and were therefore not inherently controlled. In addition, some important time-varying confounders, including co-pollutants, were not adjusted. These limitations should be acknowledged and discussed more explicitly.
(5) Wildfire and non-wildfire PM2.5 have very different exposure distributions, with wildfire PM2.5 levels being much lower and highly skewed. Presenting both effects per 1 µg/m³ increase is therefore not comparable and overstates the impact of wildfire PM2.5, since a 1 µg/m³ change is unrealistic for its long-term averages. Using an IQR or SD increment would provide a more appropriate and interpretable comparison.
(6) The study included hospitalization data from only 20 US states. Please provide rationale for selecting these states and discuss generalizability.
(7) The study period varied substantially across states. For example, California contributes only two years of data. States with short observation periods may be under-represented, potentially biasing results. Please discuss the implications.
(8) The authors exclude observations where non-wildfire PM2.5 values were negative after subtracting wildfire-specific PM2.5 from total PM2.5. Although these cases represent a small proportion of the data (<0.01%), such cases are most likely to occur on high-smoke days, when wildfire PM2.5 is large and model uncertainty is greatest. Removing these observations may disproportionately exclude high wildfire PM2.5 exposure periods and underestimate annual wildfire PM2.5 averages. The authors should justify this exclusion, assess its impact, and consider alternative approaches (e.g., truncation at zero rather than deletion).
(9) The calculation of non-wildfire PM2.5 by subtracting wildfire-specific PM2.5 (Childs et al.) from total PM2.5 (Di et al.) may introduce substantial measurement error because the two input products differ in data sources, modeling approaches, spatial resolution, and uncertainty structures. Please discuss the impact on the estimated associations and their comparison.

(10) The study used ZIP code-level covariates only from the Decennial Census (2000, 2010, 2020), with all intermediate years imputed by linear interpolation. This may introduce substantial covariate misclassification. Please consider using the American Community Survey dataset, which provides annually updated ZIP code-level demographic and socioeconomic data for most of the study period.
(11) Please clarify whether individuals can contribute more than one hospitalization event. If multiple events per person are included, the analysis should account for within-person correlation, for example, using robust standard errors.
Minor:
(1) Please report both counts and percentages in Table 1.
(2) Please provide a table with more detailed distribution of the exposure variables (e.g., means, medians, percentiles, and variability measures). This would help assess the plausibility of the exposure contrasts used in the analysis and the robustness of the findings.
(3) Please describe all sensitivity analyses in Methods, not only in Results.
(4) The associations with CVD outcomes were in general stronger among people under 65 years. What could be the explanations?

(Remarks on code availability)

Reviewer #2

(Remarks to the Author)
The manuscript by Zhang et al. analyzed cardiopulmonary hospitalizations across 20 US states from 2006 to 2019, linking each patient ZIP code to 2-year average exposures to wildfire-specific and non-wildfire PM2.5. The authors implemented an extended self-controlled case-crossover design, comparing each hospitalization year with the years immediately before and after to estimate long-term effects while inherently controlling for all time-invariant individual confounders. Conditional logistic regression models assessed associations between both PM2.5 sources and hospitalization risk, adjusting for neighborhood sociodemographic characteristics, temperature, and calendar year. The study found that long-term exposure to PM2.5 from wildfire smoke was more strongly associated with increased hospitalization risks than non-wildfire PM2.5 for all major cardiovascular and respiratory diseases, with the largest impacts observed for cerebrovascular disease, ischemic heart disease, and asthma. Both PM2.5 sources disproportionately affected Hispanic and other minority groups, residents of metropolitan or socioeconomically disadvantaged communities, and individuals with obesity or diabetes, indicating substantial inequities in vulnerability to particle pollution.

Major Comments

1. The Authors use ‘2-year averages of wildfire-specific PM2.5 concentrations’ as their principal measure of long-term exposure when evaluating the cardiopulmonary hospitalization risk. Wildfire events generate increases in PM2.5 that may persist from several weeks to several months. When these episodic peaks are averaged over a 2-year period, their intensity and timing are substantially diluted. As a result, the exposure metric may not accurately reflect short-term fluctuations that could precipitate acute cardiopulmonary events. This raises a central methodological concern: the analysis assumes that hospitalizations stem from chronic, long-term exposure, yet the chosen exposure window used does not permit differentiation between chronic effects and acute responses to wildfire-related PM2.5 spikes.

2. In addition, caution is warranted when using ambient PM2.5 concentrations as a proxy for individual exposure, as factors such as time spent outdoors, physical activity levels, indoor ventilation efficiency, and other behavioral or environmental variables can substantially influence the actual inhaled and deposited doses associated with cardiopulmonary health effects requiring hospitalization.

3. In Figure 1, the color scales used for wildfire-specific and non-wildfire PM2.5 differ, which may inadvertently give the impression that wildfire-specific PM2.5 is more substantial than it actually is. If applying a unified scale would render panel A uniformly blue, this limitation should be clearly acknowledged in the figure caption to prevent misinterpretation.

4. Among the 20 states included, California, Oregon, and Washington appear to be the only ones experiencing high-intensity PM2.5 exposure from both wildfire-specific and non-wildfire sources, whereas in the remaining states non-wildfire PM2.5 predominates. Have the Authors conducted any region-specific comparisons of the relative cardiopulmonary risks attributable to wildfire versus non-wildfire PM2.5 sources?

5. As additional motivation for such an analysis, the vegetative fuels that sustain fires in southern California differ substantially from those in the Pacific Northwest. These differences result in distinct chemical compositions of particulate matter (as well as other gaseous components of smoke not captured in this analysis), which influence smoke toxicity and are likely to modulate the resulting cardiopulmonary health effects.

6. In Figure 3, the lower bound of the color bar is unclear. Many of the tiles shaded in red appear to have confidence intervals that include a relative risk of 1. Should these be interpreted as indicating no meaningful change in relative risk rather than a decrease? Clarifying this point would make the figure easier to interpret.

7. In Figure 4, the meaning of the shaded areas is unclear. Some of the stratified results are also difficult to interpret. For instance, in the top-left panel, individuals without obesity appear to have a higher risk of hypertension following long-term exposure to wildfire-specific PM2.5 than individuals with obesity. Similar patterns are shown for acute respiratory infection

and diabetes. These findings run counter to established evidence that obesity and diabetes increase cardiopulmonary risk.

Minor Comments

1. The Authors adjusted for unemployment, overcrowded households, non-ownership of a car, and non-ownership of a home as potential confounders. These variables constitute the components of the Townsend Deprivation Index, linked to ZIP code as an indicator of overall material deprivation. However, this index does not incorporate measures of geographic access to services, including routine healthcare beyond the recorded hospital visit, which may also function as a confounding factor.

2. The explanation of the self-controlled study design is barebone, making it hard to understand how it works and why it is advantageous.

3. In the Discussion, the finding that individuals residing in metropolitan areas were more susceptible than those in micropolitan or non-CBSA regions is attributed to higher levels of social stress, which may negatively affect immune responses and increase vulnerability to environmental stressors. However, this explanation is not sufficiently developed. The manuscript does not describe nor cite evidence supporting links between environmental stressors, immune responses, and cardiopulmonary disease, making the rationale incomplete and not fully justified.

(Remarks on code availability)

Version 1:

Reviewer comments:

Reviewer #1

(Remarks to the Author)
The authors have done a great job in this revision. I have no further comments. Congratulations!

(Remarks on code availability)

Reviewer #3

(Remarks to the Author)
The authors have addressed the previous concerns adequately and the revised manuscript is improved. The added justification for the self-controlled design is clearer and the additional sensitivity analyses strengthen the robustness of the findings, particularly the analyses using alternative control-year specifications and exposure windows. The responses are detailed, transparent, and methodologically sound. The study is good and important given the increasing contribution of wildfire smoke to ambient PM2.5 exposure and the large sample size and direct comparison between wildfire-specific and non-wildfire PM2.5 provide evidence regarding differential toxicity and long-term cardiopulmonary risks. I find the response to reviewer #2’s concerns convincing overall. I only have a few minor suggestions. Some methodological explanations regarding the self-controlled framework are repeated across the manuscript and could be shortened for clarity. The discussion should also acknowledge more explicitly that overlapping exposure windows would likely attenuate exposure contrasts and bias estimates toward the null rather than inflate associations. In addition, parts of the Discussion comparing prior wildfire studies could be shortened slightly to improve readability. Overall, I believe the manuscript is suitable for publication.

(Remarks on code availability)
NA

Open Access This Peer Review File is licensed under a Creative Commons Attribution 4.0 International License, which permits use, sharing, adaptation, distribution and reproduction in any medium or format, as long as you give appropriate credit to the original author(s) and the source, provide a link to the Creative Commons license, and indicate if changes were made.
In cases where reviewers are anonymous, credit should be given to 'Anonymous Referee' and the source.
The images or other third party material in this Peer Review File are included in the article's Creative Commons license, unless indicated otherwise in a credit line to the material. If material is not included in the article's Creative Commons license and your intended use is not permitted by statutory regulation or exceeds the permitted use, you will need to obtain permission directly from the copyright holder.
To view a copy of this license, visit https://creativecommons.org/licenses/by/4.0/

Response to reviewers

Reviewer #1 (Remarks to the Author):

This study compared the associations between long-term exposure to $PM_{2.5}$ derived from wildfires versus non-wildfire sources on cardiopulmonary hospitalizations using a dataset of over 89 million hospitalization records across 20 US states (2006–2019). The authors employed a self-controlled study design to estimate the hospitalization risks associated with 2-year moving average exposures. The main finding is that for each 1 $\mu g/m^3$ increase, the effects of wildfire-specific $PM_{2.5}$ were significantly stronger than those of non-wildfire $PM_{2.5}$ across all examined cardiopulmonary outcomes. While the large dataset and examination of multiple cause-specific cardiopulmonary outcomes are strengths, I have several concerns regarding the methodology and interpretation of the comparative toxicity findings.

Response: Thank you very much for your careful review of our manuscript and for your thoughtful comments. We greatly appreciate your insights regarding the study design and the interpretation of our findings. In response, we have carefully revised the manuscript to address these concerns more explicitly. Below, we provide a point-by-point response to your comments and describe the corresponding revisions made to the manuscript.

Major:

(1) The self-controlled design, as an extension of the traditional case-crossover design, was not sufficiently justified in the manuscript. The authors should provide methodological justification or supporting references, and examine how sensitive the results are to the choice of control years. A comparison with estimates from a more standard Cox or Poisson regression would help assess the robustness of the findings.

Response: Thanks for this constructive comment. We have revised the manuscript to more clearly justify the use of the self-controlled design and to assess the robustness of our findings.

First, we clarified that the self-controlled design used in this study is an extension of

the traditional case-crossover framework, which compares exposure during hazard periods with exposure during control periods within the same individual and inherently controls for time-invariant confounders. While the case-crossover design was originally developed to study acute effects, we previously extended this self-controlled framework to examine associations between medium-term (3-month average) exposure to wildfire smoke $PM_{2.5}$ and hospitalization risks across a comprehensive range of cardiovascular and respiratory diseases (Wei et al., 2025). That work demonstrated the feasibility and validity of applying this approach to subacute exposure windows and yielded consistent and biologically plausible associations (Wei et al., 2025; Wei et al., 2026). The underlying principle of the case-crossover framework is a within-individual comparison of exposure levels between a case period and control periods, typically analyzed using conditional logistic regression with each individual forming a matched stratum (Janes et al., 2005; Gault et al., 2017). If exposure is causally related to the event, exposure levels during the case period are expected to differ from those during the control periods. Although the traditional case-crossover design focuses on short-term exposures, its validity fundamentally relies on within-individual contrasts under the assumption that time-invariant confounders remain constant across comparison periods. When exposure is defined as multi-year averages, the same logic applies as long as exposure contrasts exist between case and control years. Building on this prior work, the current study applies the same self-controlled framework to investigate long-term effect. We have added this methodological justification and relevant references to the Methods section of the revised manuscript (Page 22-23, Line 426-441).

Second, to address concerns regarding the choice of control years, we conducted additional sensitivity analyses using alternative control-year specifications, including selecting two, three, and four years before and after the year of hospitalization (case year) as control years, respectively. The associations between wildfire-specific and non-wildfire $PM_{2.5}$ and the hospitalization risk were consistent with our primary analysis, with slightly changing estimate sizes (Table R1; Supplementary Table 11), indicating that the overall associations were not sensitive to the selection of control

years. For example, when selecting two years before and after the year of hospitalization (case year) as control years, we found that each 1-$\mu g/m^3$ increase in 2-year wildfire-specific $PM_{2.5}$ was significantly associated with increased hospitalization risks for all cardiopulmonary diseases, with relative risk ranging from 1.081 (95% CI: 1.076, 1.088) for heart failure to 1.254 (95% CI: 1.248, 1.260) for hypertension. In comparison, a 1 $\mu g/m^3$ increase in non-wildfire $PM_{2.5}$ was associated with increased hospitalization risks for all cardiopulmonary diseases, but with relative risks ranging from 1.037 (95% CI: 1.034, 1.040) for COPD to 1.082 (95% CI: 1.080, 1.084) for hypertension. However, as the control year moves further away from the case year, unmeasured slow-varying confounders are more likely to change, and the sample size tends to decrease. For this reason, we selected the year immediately before and after the case year as control years, in order to better account for time-varying confounding while minimizing the loss of sample size.

Furthermore, we agree that comparing results with standard Cox or Poisson regression models would be informative. However, due to the structure of the SID database, such analyses are unfortunately not appropriate in our study setting. First, SID includes only hospitalization records and does not provide data on non-hospitalized individuals. Therefore, we cannot construct appropriate risk sets or person-time denominators required for a Cox proportional hazards model. Second, a Poisson regression would require valid population denominators at the ZIP-code level. Because ZIP code boundaries are not definitive, population estimates at the ZIP code level may be subject to substantial bias. Importantly, all measurable covariates available in SID (individual-level factors, disease severity, and neighborhood-level characteristics) have already been adjusted for in our self-controlled design. In addition, this design inherently controls for time-invariant and slowly varying unmeasured confounders, which conventional Cox or Poisson models would not adequately address under the current data constraints. For these reasons, we believe the self-controlled approach is methodologically appropriate and preferable given the structure of the SID data. We have clarified this point in the revised manuscript.

Revised text in the Methods and Results:

Page 22-23, Line 426-451 (Methods): In our previous study (Wei 2025), we extended the traditional case-crossover design, a self-controlled design originally developed for studying short-term transient effects and widely used in environmental epidemiology (Wei 2026; Janes 2005; Consiglio 2013), to evaluate medium-term effects. The underlying principle of the case-crossover framework is a within-individual comparison of exposure levels between a case period and control periods, typically analyzed using conditional logistic regression with each individual forming a matched stratum. If exposure is causally related to the event, exposure levels during the case period are expected to differ from those during the control periods. Although the traditional case-crossover design focuses on short-term exposures, its validity fundamentally relies on within-individual contrasts under the assumption that time-invariant confounders remain constant across comparison periods. When exposure is defined as multi-year averages, the same logic applies as long as exposure contrasts exist between case and control years.

In this study, we further extended this framework to evaluate long-term effects of 2-year average exposures to wildfire-specific and non-wildfire $PM_{2.5}$ on cardiopulmonary hospitalization risks. Specifically, for each case year (year of hospitalization), the years before and after served as that hospitalization's own controls. With this self-controlled design, unmeasured time-invariant and slowly varying confounders, such as genetics, chronic coexisting conditions (e.g., diabetes), and smoking status, are inherently adjusted by design. The symmetric bidirectional selection of control years before and after a case year removed the unmeasured

删除[Min Zhang]: The self-controlled design adjusts for all time-invariant and slowly varying confounders through self-matching, regardless of whether these confounders are measured or not.

删除[Min Zhang]: the traditional case-crossover design, a self-controlled design originally developed for studying short-term effects and widely used,

confounding from long-term temporal trends (e.g., gradual changes in population-level wildfire smoke level). Selecting one single year before and after the case minimized temporal variations in unmeasured confounders.

Page 24, Line 461-470 (Methods): To test the robustness of our results, we performed the following sensitivity analyses: 1) changed the degrees of freedom for temperature to 9; 2) additionally adjusted for the mean and standard deviation of summer and winter relative humidity; 3) additionally adjusted for annual concentrations of nitrogen dioxide and ozone; 4) identified hospitalizations based on the principal diagnosis code at discharge; 5) truncated negative non-wildfire $PM_{2.5}$ values to zero in the models, rather than deleted the negative values; 6) assigned 3-year and 5-year moving averages of $PM_{2.5}$ concentrations as alternative long-term exposure measures; 7) selected two, three, and four years before and after the year of hospitalization (case year) as control years, respectively; 8) to dertermine region-specific differences, we further performed stratified analyses by US state.

Page 10-11, Line 160-180 (Results): Overall, the associations between long-term exposure to wildfire-specific $PM_{2.5}$ and non-wildfire $PM_{2.5}$ and risks of cardiopulmonary hospitalizations remained robust after changing covariates adjusted in the model (Supplementary Table 5-6). The effect estimates remained largely unchanged when hospitalizations were identified based solely on the principal diagnosis code at discharge (Supplementary Table 7) or when negative values of non-wildfire $PM_{2.5}$ were modeled as zero instead of being excluded (Supplementary Table 8). When alternative exposure windows were considered using 3- or 4-year

删除[Min Zhang [2]]: These results remained robust after changing the degrees of freedom for temperature up to 9, adjusting for annual precipitation and vapor pressure, or identifying hospitalizations based on the principal diagnosis code at discharge (eTable 3-5 in **Supplement 1**).

moving averages, the association between wildfire-specific $PM_{2.5}$ and cardiopulmonary hospitalizations became stronger with longer exposure duration, whereas the association with non-wildfire $PM_{2.5}$ remained relatively stable (Supplementary Table 9). Furthermore, results were consistent under alternative control-year specifications, indicating that the self-controlled design was not materially sensitive to the selection of control years (Supplementary Table 10). In stratified analyses by US state, associations appeared stronger in states with high $PM_{2.5}$ levels, particularly in California (Supplementary Table 11).

Table R1. Sensitivity analysis results selecting two, three, and four years before and after the year of hospitalization (case year) as control years, respectively.

Outcomes	Relative risk (95% CI) per 1 μg/m^3 increase		Relative risk (95% CI) per SD μg/m^3 increase	
	Wildfire-specific $PM_{2.5}$	Non-wildfire $PM_{2.5}$	Wildfire-specific $PM_{2.5}$	Non-wildfire $PM_{2.5}$
Two years before and after the year of hospitalization (case year)				
Ischemic heart disease	1.200 (1.192, 1.208)	1.071 (1.069, 1.074)	1.063 (1.061, 1.066)	1.157 (1.152, 1.163)
Cerebrovascular disease	1.189 (1.180, 1.198)	1.078 (1.075, 1.081)	1.064 (1.061, 1.067)	1.167 (1.160, 1.174)
Heart failure	1.082 (1.076, 1.088)	1.062 (1.060, 1.064)	1.028 (1.026, 1.030)	1.131 (1.126, 1.137)
Arrhythmia	1.152 (1.144, 1.159)	1.047 (1.044, 1.049)	1.049 (1.047, 1.052)	1.100 (1.094, 1.105)
Hypertension	1.254 (1.248, 1.260)	1.082 (1.080, 1.084)	1.080 (1.079, 1.082)	1.175 (1.171, 1.180)
Other cardiovascular diseases	1.160 (1.154, 1.167)	1.064 (1.062, 1.066)	1.053 (1.051, 1.055)	1.137 (1.132, 1.141)
Acute respiratory infections	1.229 (1.209, 1.249)	1.069 (1.063, 1.075)	1.071 (1.065, 1.077)	1.148 (1.135, 1.162)
Pneumonia	1.170 (1.162, 1.177)	1.062 (1.060, 1.065)	1.057 (1.054, 1.059)	1.135 (1.129, 1.140)
COPD	1.161 (1.152, 1.170)	1.037 (1.034, 1.040)	1.050 (1.048, 1.053)	1.080 (1.073, 1.086)
Asthma	1.241 (1.227, 1.255)	1.068 (1.065, 1.072)	1.066 (1.063, 1.070)	1.143 (1.135, 1.152)
Other respiratory diseases	1.183 (1.178, 1.189)	1.079 (1.077, 1.081)	1.069 (1.067, 1.071)	1.168 (1.164, 1.172)
Ischemic heart disease	1.200 (1.192, 1.208)	1.071 (1.069, 1.074)	1.063 (1.061, 1.066)	1.157 (1.152, 1.163)
Three years before and after the year of hospitalization (case year)				
Ischemic heart disease	1.264 (1.256, 1.271)	1.080 (1.078, 1.082)	1.082 (1.080, 1.084)	1.177 (1.172, 1.182)
Cerebrovascular disease	1.295 (1.286, 1.304)	1.064 (1.061, 1.066)	1.098 (1.095, 1.100)	1.136 (1.130, 1.141)

Heart failure	1.312 (1.305, 1.319)	1.076 (1.074, 1.078)	1.099 (1.097, 1.101)	1.163 (1.158, 1.167)
Arrhythmia	1.292 (1.284, 1.299)	1.064 (1.062, 1.066)	1.091 (1.089, 1.093)	1.138 (1.133, 1.142)
Hypertension	1.256 (1.250, 1.261)	1.096 (1.095, 1.098)	1.081 (1.079, 1.083)	1.209 (1.205, 1.212)
Other cardiovascular diseases	1.241 (1.234, 1.247)	1.057 (1.055, 1.059)	1.078 (1.076, 1.080)	1.121 (1.117, 1.125)
Acute respiratory infections	1.215 (1.197, 1.233)	1.077 (1.072, 1.082)	1.067 (1.062, 1.072)	1.165 (1.154, 1.177)
Pneumonia	1.264 (1.257, 1.271)	1.048 (1.046, 1.050)	1.086 (1.084, 1.088)	1.103 (1.098, 1.107)
COPD	1.253 (1.245, 1.262)	1.055 (1.053, 1.058)	1.077 (1.075, 1.080)	1.121 (1.116, 1.127)
Asthma	1.274 (1.261, 1.287)	1.056 (1.053, 1.060)	1.075 (1.071, 1.078)	1.118 (1.110, 1.125)
Other respiratory diseases	1.308 (1.302, 1.313)	1.070 (1.068, 1.071)	1.112 (1.110, 1.114)	1.148 (1.144, 1.151)
Ischemic heart disease	1.264 (1.256, 1.271)	1.080 (1.078, 1.082)	1.082 (1.080, 1.084)	1.177 (1.172, 1.182)
Four years before and after the year of hospitalization (case year)				
Ischemic heart disease	1.222 (1.215, 1.229)	1.085 (1.083, 1.087)	1.070 (1.068, 1.072)	1.190 (1.185, 1.195)
Cerebrovascular disease	1.257 (1.249, 1.266)	1.062 (1.059, 1.064)	1.086 (1.083, 1.088)	1.131 (1.126, 1.137)
Heart failure	1.340 (1.334, 1.347)	1.065 (1.063, 1.067)	1.107 (1.105, 1.109)	1.138 (1.134, 1.142)
Arrhythmia	1.273 (1.266, 1.281)	1.049 (1.047, 1.051)	1.086 (1.084, 1.088)	1.104 (1.100, 1.109)
Hypertension	1.181 (1.176, 1.186)	1.123 (1.121, 1.125)	1.059 (1.057, 1.060)	1.270 (1.266, 1.273)
Other cardiovascular diseases	1.220 (1.215, 1.226)	1.058 (1.056, 1.060)	1.072 (1.070, 1.074)	1.122 (1.118, 1.126)
Acute respiratory infections	1.160 (1.144, 1.176)	1.094 (1.089, 1.099)	1.051 (1.046, 1.056)	1.202 (1.191, 1.214)
Pneumonia	1.248 (1.242, 1.255)	1.040 (1.038, 1.042)	1.081 (1.079, 1.083)	1.086 (1.082, 1.090)
COPD	1.163 (1.156, 1.171)	1.041 (1.039, 1.044)	1.051 (1.049, 1.053)	1.090 (1.085, 1.095)
Asthma	1.230 (1.218, 1.241)	1.064 (1.061, 1.067)	1.063 (1.060, 1.066)	1.134 (1.127, 1.141)
Other respiratory diseases	1.303 (1.297, 1.308)	1.073 (1.071, 1.074)	1.110 (1.109, 1.112)	1.155 (1.151, 1.158)
Ischemic heart disease	1.222 (1.215, 1.229)	1.085 (1.083, 1.087)	1.070 (1.068, 1.072)	1.190 (1.185, 1.195)
Note: The model was adjusted for calendar year, percentage of White, percentage of Black, percentage of the population who graduated high school, median household income, townsend deprivation index, mean temperature of summer and winter, and standard deviation of summer and winter temperature.				

Reference:

1. Wei Y, Castro E, Yin K, et al. Medium-term Exposure to Wildfire Smoke PM 2.5 and Cardiorespiratory Hospitalization Risks. Epidemiology. 2025;36(5):606-615. doi:10.1097/EDE.0000000000001881.

2. Wei Y, Schwartz JD, Zhang M, Wright RO. Case-Crossover Design for Assessing Associations With Short-Term, Intermediate-Term, and Long-Term Exposures. J Surg Res. Published online January 17, 2026. doi:10.1016/j.jss.2025.11.072.

3. Janes H, Sheppard L, Lumley T. Case-crossover analyses of air pollution exposure data: referent selection strategies and their implications for bias. Epidemiology.

2005;16(6):717-726. doi:10.1097/01.ede.0000181315.18836.9d.

4. Gault N, Castañeda-Sanabria J, De Rycke Y, Guillo S, Foulon S, Tubach F. Self-controlled designs in pharmacoepidemiology involving electronic healthcare databases: a systematic review. BMC Med Res Methodol. 2017;17(1):25. Published 2017 Feb 8. doi:10.1186/s12874-016-0278-0.

(2) The study used 2-year averages of wildfire and non-wildfire PM2.5 with substantially overlapped exposure windows for the case year and adjacent control years. As a result, the within-person variation in exposures can be very limited, especially for wildfire-specific PM2.5. This can lead to unstable effect estimates and stronger influence of measurement error.

Response: Thank you for this insightful comment. We selected a 2-year exposure average (case year and prior year) a priori to balance exposure misclassification while capturing the majority of the long-term effect. Evaluating exposure only for the year of hospitalization would avoid overlapping exposure windows but could introduce misclassification, as individuals hospitalized early in the year would be assigned an average exposure for the entire year. In contrast, limiting the exposure window to the year prior to hospitalization would exclude the hospitalization year, which is most likely to have the strongest effect based on existing literature (Wei et al., 2024).

We share your concern about limited exposure variation. However, its potential impact on the accuracy of effect estimates is fundamentally a power issue. Given that our analysis includes millions of hospitalizations (i.e., strata) for each disease category, we should be able to detect the effects even with limited exposure variation.

To fully address your concern, we quantified within-person variability in both 2-year averages of wildfire-specific and non-wildfire $PM_{2.5}$ across case and control years (Table R2). For wildfire-specific $PM_{2.5}$, the median within-person standard deviation ranged from 0.092 to 0.100 μg/m^3 across different outcomes, while for non-wildfire $PM_{2.5}$ it ranged from 0.367 to 0.400 μg/m^3, indicating the presence of within-person variability for both exposures. The smaller within-person variability observed for

wildfire-specific $PM_{2.5}$ likely reflects its substantially lower overall exposure levels (the mean concentration was 0.3 μg/m^3) and does not imply a lack of meaningful within-person exposure contrast. The ratio of within- to between-individual variability was 0.3009-0.3592 for wildfire-specific $PM_{2.5}$ and 0.06215-0.0709 for non-wildfire $PM_{2.5}$, indicating that within-person variability was relatively greater for wildfire-specific $PM_{2.5}$ than for non-wildfire $PM_{2.5}$, although between-individual variability remained the dominant source of variation for both exposures.

Table R2. The within-person variability in wildfire-specific $PM_{2.5}$ and non-wildfire $PM_{2.5}$ concentrations.

Outcomes	Wildfire-specific $PM_{2.5}$			Non-wildfire $PM_{2.5}$	
	Within-person standard deviation (IQR), μg/m^3	Within-to-between variance ratio[a]		Within-person standard deviation (IQR), μg/m^3	Within-to-between variance ratio[a]
Ischemic heart disease	0.0963 (0.0911)	0.3332		0.391 (0.406)	0.0642
Cerebrovascular disease	0.0948 (0.0906)	0.3200		0.379 (0.391)	0.0646
Heart failure	0.0950 (0.0902)	0.3275		0.379 (0.388)	0.0645
Arrhythmia	0.0922 (0.0868)	0.3271		0.381 (0.387)	0.0635
Hypertension	0.0958 (0.0866)	0.3371		0.381 (0.402)	0.0654
Other cardiovascular diseases	0.0933 (0.0873)	0.3238		0.379 (0.387)	0.0647
Acute respiratory infections	0.0953 (0.0891)	0.3224		0.388 (0.400)	0.0667
Pneumonia	0.0940 (0.0922)	0.3186		0.381 (0.394)	0.0640
COPD	0.0922 (0.0915)	0.3391		0.387 (0.392)	0.06215
Asthma	0.1000 (0.0876)	0.3592		0.400 (0.398)	0.0709
Other respiratory diseases	0.0915 (0.0891)	0.3009		0.367 (0.382)	0.0630

[a] Within-to-between variance ratio is the ratio of within-to between-individual variability, which was calculated by dividing the within-individual variance by the between-individual variance.

Reference:

Wei Y, Feng Y, Danesh Yazdi M, et al. Exposure-response associations between chronic exposure to fine particulate matter and risks of hospital admission for major

cardiovascular diseases: population based cohort study. BMJ. 2024;384:e076939. Published 2024 Feb 21. doi:10.1136/bmj-2023-076939.

(3) Please justify the choice of using 2-year moving averages to represent long-term exposure and conduct sensitivity analyses using alternative averaging periods, such as 1-year and 3-year averages, to demonstrate that the findings are robust to the definition of the long-term exposure metric.

Response: We selected a 2-year exposure average (case year and prior year) a priori to balance exposure misclassification while capturing the majority of the long-term effect. Evaluating exposure only for the year of hospitalization would avoid overlapping exposure windows but could introduce misclassification, as individuals hospitalized early in the year would be assigned an average exposure for the entire year. In contrast, limiting the exposure window to the year prior to hospitalization would exclude the hospitalization year, which is most likely to have the strongest effect based on existing literature (Wei et al., 2024). This rationale has now been explicitly described in the revised Methods section.

To assess the robustness of our findings to the definition of long-term exposure, we conducted sensitivity analyses using alternative averaging periods, including 3-year and 4-year moving averages. The results were highly consistent with those from our main analysis, which used 2-year moving averages as exposure windows (Table R3; Supplementary Table 9). Across all averaging periods examined, both wildfire-specific $PM_{2.5}$ and non-wildfire $PM_{2.5}$ were associated with an increased risk of cardiopulmonary hospitalizations. Moreover, for all of the associations, stronger wildfire-specific $PM_{2.5}$ effects on risk of cardiopulmonary hospitalizations than non-wildfire $PM_{2.5}$ per 1 $\mu g/m^3$ change in exposure. These findings indicate that our results are robust to alternative definitions of long-term exposure. Moreover, we observed that at longer exposure windows, the risk of cardiopulmonary hospitalization associated with wildfire-specific $PM_{2.5}$ increased, whereas the risk associated with non-wildfire $PM_{2.5}$ remained relatively stable. These findings are

consistent with previous study (Hao et al., 2026). Together, our results suggest that wildfire-specific $PM_{2.5}$ may exert stronger cumulative toxic effects over longer exposure periods compared with non-wildfire $PM_{2.5}$, and may further indicate that a per-unit increase in wildfire-specific $PM_{2.5}$ is more harmful than a comparable increase in non-wildfire $PM_{2.5}$.

Revised text in the Manuscript:

Page 20, Line 384-386 (Methods): For each hospitalization, the exposure window of interest was defined as the 2-year averages of wildfire-specific $PM_{2.5}$ concentrations, covering both the year of hospitalization and the year before. We selected this exposure window a priori to balance capturing longer-term exposure with minimizing potential exposure misclassification. Using only the hospitalization year could introduce misclassification, as individuals admitted early in the year would be assigned exposure averaged over the entire calendar year. Conversely, restricting exposure to the year prior to hospitalization would exclude the hospitalization year, which may have the strongest effect based on prior evidence (Wei et al., 2024).

Table R3. Cardiopulmonary hospitalization risks in 3- and 4- year average of wildfire-specific $PM_{2.5}$ and non-wildfire $PM_{2.5}$ concentrations.

Outcomes	Relative risk (95% CI) per 1 μg/m^3 increase		Relative risk (95% CI) per SD μg/m^3 increase	
	Wildfire-specific $PM_{2.5}$	Non-wildfire $PM_{2.5}$	Wildfire-specific $PM_{2.5}$	Non-wildfire $PM_{2.5}$
Lag02 (3-year averages)				
Ischemic heart disease	1.372 (1.352, 1.393)	1.036 (1.031, 1.040)	1.112 (1.107, 1.118)	1.080 (1.070, 1.091)
Cerebrovascular disease	1.425 (1.400, 1.450)	1.032 (1.026, 1.037)	1.136 (1.129, 1.143)	1.070 (1.058, 1.082)
Heart failure	1.319 (1.301, 1.337)	1.030 (1.025, 1.034)	1.101 (1.096, 1.106)	1.065 (1.056, 1.074)
Arrhythmia	1.367 (1.346, 1.387)	1.024 (1.019, 1.028)	1.112 (1.106, 1.118)	1.053 (1.043, 1.063)
Hypertension	1.409 (1.394, 1.424)	1.037 (1.034, 1.041)	1.125 (1.121, 1.129)	1.083 (1.076, 1.091)
Other cardiovascular diseases	1.369 (1.352, 1.386)	1.027 (1.023, 1.031)	1.116 (1.111, 1.121)	1.060 (1.052, 1.069)
Acute respiratory infections	1.442 (1.389, 1.497)	1.038 (1.027, 1.049)	1.130 (1.116, 1.144)	1.084 (1.06, 1.1090)
Pneumonia	1.334 (1.314, 1.353)	1.020 (1.015, 1.024)	1.106 (1.101, 1.112)	1.045 (1.035, 1.055)
COPD	1.267 (1.245, 1.288)	1.013 (1.008, 1.019)	1.081 (1.075, 1.087)	1.031 (1.020, 1.043)
Asthma	1.472 (1.434, 1.511)	1.042 (1.035, 1.050)	1.122 (1.113, 1.130)	1.090 (1.075, 1.106)

Other respiratory diseases	1.408 (1.393, 1.422)	1.029 (1.025, 1.032)	1.145 (1.141, 1.150)	1.064 (1.056, 1.071)
Ischemic heart disease	1.372 (1.352, 1.393)	1.036 (1.031, 1.040)	1.112 (1.107, 1.118)	1.080 (1.070, 1.091)
Lag03 (4-year averages)				
Ischemic heart disease	1.560 (1.531, 1.590)	1.066 (1.060, 1.072)	1.161 (1.154, 1.169)	1.150 (1.136, 1.164)
Cerebrovascular disease	1.590 (1.555, 1.625)	0.991 (0.987, 0.996)	1.175 (1.166, 1.184)	1.149 (1.133, 1.166)
Heart failure	1.550 (1.524, 1.575)	1.053 (1.048, 1.059)	1.164 (1.157, 1.170)	1.117 (1.105, 1.129)
Arrhythmia	1.526 (1.498, 1.554)	1.055 (1.049, 1.061)	1.154 (1.147, 1.162)	1.122 (1.108, 1.136)
Hypertension	1.676 (1.655, 1.698)	1.082 (1.078, 1.087)	1.193 (1.188, 1.199)	1.183 (1.172, 1.193)
Other cardiovascular diseases	1.540 (1.517, 1.564)	1.062 (1.056, 1.067)	1.163 (1.156, 1.169)	1.135 (1.124, 1.147)
Acute respiratory infections	1.634 (1.560, 1.711)	1.068 (1.053, 1.082)	1.178 (1.160, 1.196)	1.149 (1.117, 1.182)
Pneumonia	1.591 (1.563, 1.620)	1.042 (1.036, 1.048)	1.177 (1.170, 1.184)	1.093 (1.081, 1.106)
COPD	1.497 (1.466, 1.529)	1.025 (1.018, 1.032)	1.143 (1.135, 1.150)	1.057 (1.042, 1.072)
Asthma	1.688 (1.633, 1.745)	1.069 (1.059, 1.079)	1.168 (1.157, 1.180)	1.147 (1.126, 1.169)
Other respiratory diseases	1.583 (1.564, 1.602)	1.055 (1.051, 1.060)	1.200 (1.194, 1.205)	1.122 (1.113, 1.132)
Ischemic heart disease	1.560 (1.531, 1.590)	1.066 (1.060, 1.072)	1.161 (1.154, 1.169)	1.150 (1.136, 1.164)
Note: The model was adjusted for calendar year, percentage of White, percentage of Black, percentage of the population who graduated high school, median household income, townsend deprivation index, mean temperature of summer and winter, and standard deviation of summer and winter temperature.				

Reference:

Hao H, Xu K, Zhang D, et al. Long-term exposure to wildfire smoke particulate matter and incident stroke: a US nationwide study. Eur Heart J. Published online January 27, 2026. doi:10.1093/eurheartj/ehaf875.

Wei Y, Feng Y, Danesh Yazdi M, et al. Exposure-response associations between chronic exposure to fine particulate matter and risks of hospital admission for major cardiovascular diseases: population based cohort study. BMJ. 2024;384:e076939. Published 2024 Feb 21. doi:10.1136/bmj-2023-076939.

(4) The manuscript overstates the confounding control by the self-controlled design. While time-invariant characteristics are addressed, several important factors, such as chronic disease status, medication use (which probably change following cardiopulmonary hospitalizations), and smoking behavior, can change substantially

over the 3-year case-control window and were therefore not inherently controlled. In addition, some important time-varying confounders, including co-pollutants, were not adjusted. These limitations should be acknowledged and discussed more explicitly.

Response: We agree that the self-controlled design does not inherently control for all potential confounders. Although this design accounts for some time-invariant characteristics, several individual-level factors, such as chronic disease status, medication use, and smoking behavior, may change over the 3-year case-control window and therefore are not fully controlled. Information on these factors was not available in our data. In addition, residual confounding by some important time-varying confounders may remain. These limitations have now been more explicitly acknowledged and discussed in the revised manuscript, particularly in the Limitations section.

Revised text in the Discussion:

Page 17-18, Line 325-329: Fifth, residual confounding may remain due to unmeasured time-varying factors, including individual behaviors, indoor exposures, medication use, and co-pollutants (e.g., nitrogen dioxide and ozone), as well as differences between ambient $PM_{2.5}$ concentrations and actual personal exposure.

(5) Wildfire and non-wildfire $PM_{2.5}$ have very different exposure distributions, with wildfire $PM_{2.5}$ levels being much lower and highly skewed. Presenting both effects per 1 μg/m³ increase is therefore not comparable and overstates the impact of wildfire PM2.5, since a 1 μg/m³ change is unrealistic for its long-term averages. Using an IQR or SD increment would provide a more appropriate and interpretable comparison.

Response: Thank you for this thoughtful comment. We present effect estimates per 1 μg/m³ increase to place wildfire and non-wildfire $PM_{2.5}$ on the same exposure scale, thereby allowing a direct comparison of their relative toxicity. When comparing the two $PM_{2.5}$ types, all effect estimates are expressed using the same increment, ensuring consistency in scale.

We agree that per-IQR or per-SD increments better reflect realistic exposure contrasts

within each pollutant. However, because wildfire and non-wildfire $PM_{2.5}$ have markedly different distributions, using distribution-based increments would make the resulting effect estimates not directly comparable across pollutant types.

To provide additional context, we have included per-SD results in the Supplementary Material (Table R4; Supplementary Table 2) and added discussion in the manuscript clarifying the differences in exposure distributions and corresponding effect estimates.

Revised text in the Manuscript:

Page 9, Line 136-138 (Results): Compared to non-wildfire $PM_{2.5}$, a 1-μg/m^3 increase in wildfire-specific $PM_{2.5}$ was associated with greater risks of hospitalizations for all the examined cardiopulmonary diseases (**Figure 2**).

Page 10, Line 155-158 (Results): However, given the substantially lower concentrations of wildfire-specific $PM_{2.5}$, the cardiopulmonary hospitalization risks increase per standard deviation is greater for non-wildfire $PM_{2.5}$ (Supplementary Table 4).

Page 15, Line 259-267 (Discussion): Because components of wildfire-specific $PM_{2.5}$ are smaller in size, they may more easily penetrate the human body, which facilitate their adverse effects. Furthermore, although the risk per SD increase was approximately three times higher for non-wildfire $PM_{2.5}$ than for wildfire-specific $PM_{2.5}$, the SD of non-wildfire $PM_{2.5}$ was about six times larger. Importantly, under future climate change scenarios, wildfire activity in the US is expected to increase substantially, with annual wildfire-specific $PM_{2.5}$ concentrations potentially reaching 10 μg/m^3 in some regions (Qiu et al., 2025). Therefore, despite its currently lower concentrations, the public health impact of wildfire-specific $PM_{2.5}$ should not be underestimated.

Page 18, Line 336-338 (Conclusion): This study demonstrates that, at an equivalent concentration increase, long-term exposure to wildfire-specific $PM_{2.5}$ poses a greater risk of cardiopulmonary hospitalization than non-wildfire $PM_{2.5}$.

Table R4. The hospitalization risks of cardiopulmonary diseases associated with a SD increase in 2-year average of wildfire-specific $PM_{2.5}$ and non-wildfire $PM_{2.5}$

concentrations.

Outcomes	Wildfire-specific $PM_{2.5}$		Non-wildfire $PM_{2.5}$	
	SD, μg/m^3	Relative risk (95% CI)	SD, μg/m^3	Relative risk (95% CI)
Ischemic heart disease	0.336	1.042 (1.039, 1.045)	2.124	1.143 (1.135, 1.151)
Cerebrovascular disease	0.360	1.045 (1.041, 1.049)	2.061	1.152 (1.142, 1.162)
Heart failure	0.346	1.033 (1.031, 1.036)	2.049	1.115 (1.108, 1.122)
Arrhythmia	0.340	1.038 (1.035, 1.042)	2.084	1.111 (1.103, 1.119)
Hypertension	0.342	1.040 (1.038, 1.042)	2.059	1.183 (1.177, 1.189)
Other cardiovascular diseases	0.349	1.040 (1.037, 1.042)	2.055	1.133 (1.126, 1.139)
Acute respiratory infections	0.333	1.036 (1.028, 1.043)	2.061	1.114 (1.095, 1.133)
Pneumonia	0.351	1.037 (1.034, 1.040)	2.087	1.121 (1.113, 1.128)
COPD	0.330	1.036 (1.032, 1.039)	2.124	1.101 (1.092, 1.111)
Asthma	0.297	1.045 (1.040, 1.050)	2.022	1.125 (1.113, 1.137)
Other respiratory diseases	0.396	1.044 (1.041, 1.046)	2.044	1.139 (1.133, 1.145)
Ischemic heart disease	0.336	1.042 (1.039, 1.045)	2.124	1.143 (1.135, 1.151)

Note: The model was adjusted for calendar year, percentage of White, percentage of Black, percentage of the population who graduated high school, median household income, townsend deprivation index, mean temperature of summer and winter, and standard deviation of summer and winter temperature, annual NO2 concentration, and annual O3 concentration.

Reference:

Qiu M, Li J, Gould CF, et al. Wildfire smoke exposure and mortality burden in the USA under climate change. Nature. 2025;647(8091):935-943. doi:10.1038/s41586-025-09611-w.

(6) The study included hospitalization data from only 20 US states. Please provide rationale for selecting these states and discuss generalizability.

Response: The study included hospitalization data from 20 U.S. states because we purchased the State Inpatient Databases (SID) only for these states. Data from other states were not yet available to us through this source. While this limits the geographic coverage, the included states represent a diverse set of regions across the United States. We have now added a discussion in the revised manuscript addressing

the potential implications for generalizability and noting that findings may not fully represent states not included in the analysis.

Revised text in the Discussion:

Page 17, Line 317-319: Third, due to data availability, this study included only 20 states with varying observation periods, and the most recent data are nearly six years old, which may limit generalizability.

删除[Min Zhang [2]]: Second,

删除[Min Zhang [2]]: used in this study

删除[Min Zhang [2]]: and since the climate has been changing rapidly and wildfires have been worsening, it is unclear whether the effects of wildfires would be the same using more current data.

(7) The study period varied substantially across states. For example, California contributes only two years of data. States with short observation periods may be under-represented, potentially biasing results. Please discuss the implications.

Response: Thank you for this comment. We acknowledge that the study period varied across states, with some states, such as California, contributing a relatively short observation period, restricted by available data sources. For example, the ZIP code information for California was not available for years before 2018. This may lead to under-representation of certain states. We have now discussed this limitation explicitly in the revised manuscript.

In addition, in response to the second reviewer's comments, we conducted state-specific analyses (Supplementary Table 11). Even in California, despite the shorter data period (2018-2019), we observed positive associations between both wildfire-specific and non-wildfire $PM_{2.5}$ and cardiopulmonary hospitalization risk. These additional analyses provide reassurance that our main findings are not driven solely by states with longer observation periods.

Revised text in the Discussion:

Page 17, Line 317-319: Third, due to data availability, this study included only 20 states with varying observation periods, and the most recent data are nearly six years old, which may limit generalizability.

删除[Min Zhang [2]]: Second,

删除[Min Zhang [2]]: used in this study

删除[Min Zhang [2]]: and since the climate has been changing rapidly and wildfires have been worsening, it is unclear whether the effects of wildfires would be the same using more current data.

(8) The authors exclude observations where non-wildfire $PM_{2.5}$ values were negative

after subtracting wildfire-specific $PM_{2.5}$ from total $PM_{2.5}$. Although these cases represent a small proportion of the data (<0.01%), such cases are most likely to occur on high-smoke days, when wildfire $PM_{2.5}$ is large and model uncertainty is greatest. Removing these observations may disproportionately exclude high wildfire $PM_{2.5}$ exposure periods and underestimate annual wildfire $PM_{2.5}$ averages. The authors should justify this exclusion, assess its impact, and consider alternative approaches (e.g., truncation at zero rather than deletion).

Response: Non-wildfire $PM_{2.5}$ was calculated by subtracting wildfire-specific $PM_{2.5}$ (Childs et al. 2022) from total $PM_{2.5}$ (Di et al. 2019). Because these two exposure surfaces are generated using different models, negative values for non-wildfire $PM_{2.5}$ occasionally arise, reflecting model uncertainty rather than true negative concentrations. To assess whether excluding these observations disproportionately affects high wildfire $PM_{2.5}$ exposure periods or biases estimated associations, we conducted sensitivity analyses using truncating negative non-wildfire $PM_{2.5}$ values to zero. Across these alternative specifications, the effect estimates were nearly identical to those from the primary analysis (Table R5; Supplementary Table 8), indicating that our results are not sensitive to the handling of these negative values. These findings suggest that excluding observations with negative non-wildfire $PM_{2.5}$ is unlikely to materially affect estimated associations or bias inference regarding wildfire $PM_{2.5}$ exposure.

Revised text in the Discussion:

Page 17, Line 311-313: Second, estimating non-wildfire $PM_{2.5}$ by subtracting wildfire-specific $PM_{2.5}$ from total $PM_{2.5}$ may introduce non-differential measurement error due to differences in modeling approaches; however, sensitivity analyses treating negative values as zero yielded nearly identical effect sizes (Supplementary Table S8), indicating that our findings are robust to this error.

Table R5. Sensitivity analyses results using alternative approaches to handle negative values of non-wildfire $PM_{2.5}$.

Outcomes [a]	Relative risk (95% CI) per 1 μg/m³ increase	Relative risk (95% CI) per SD μg/m³ increase

	Wildfire-specific $PM_{2.5}$	Non-wildfire $PM_{2.5}$	Wildfire-specific $PM_{2.5}$	Non-wildfire $PM_{2.5}$
Ischemic heart disease	1.128 (1.118, 1.138)	1.065 (1.062, 1.069)	1.041 (1.038, 1.044)	1.143 (1.135, 1.151)
Cerebrovascular disease	1.130 (1.118, 1.141)	1.071 (1.067, 1.076)	1.045 (1.041, 1.049)	1.153 (1.143, 1.163)
Heart failure	1.099 (1.091, 1.108)	1.055 (1.051, 1.058)	1.033 (1.030, 1.036)	1.115 (1.108, 1.123)
Arrhythmia	1.116 (1.107, 1.126)	1.052 (1.048, 1.055)	1.038 (1.035, 1.041)	1.111 (1.103, 1.119)
Hypertension	1.122 (1.115, 1.129)	1.085 (1.082, 1.088)	1.040 (1.038, 1.042)	1.183 (1.177, 1.189)
Other cardiovascular diseases	1.116 (1.108, 1.125)	1.063 (1.059, 1.066)	1.039 (1.037, 1.042)	1.133 (1.126, 1.140)
Acute respiratory infections	1.111 (1.087, 1.136)	1.054 (1.045, 1.062)	1.036 (1.028, 1.043)	1.114 (1.095, 1.133)
Pneumonia	1.108 (1.099, 1.118)	1.056 (1.053, 1.060)	1.037 (1.034, 1.040)	1.121 (1.113, 1.129)
COPD	1.112 (1.101, 1.123)	1.047 (1.042, 1.051)	1.036 (1.032, 1.039)	1.102 (1.092, 1.111)
Asthma	1.159 (1.141, 1.177)	1.060 (1.054, 1.066)	1.045 (1.040, 1.050)	1.125 (1.113, 1.137)
Other respiratory diseases	1.114 (1.108, 1.120)	1.066 (1.063, 1.069)	1.044 (1.041, 1.046)	1.139 (1.134, 1.145)

Note: The model was adjusted for calendar year, percentage of White, percentage of Black, percentage of the population who graduated high school, median household income, townsend deprivation index, mean temperature of summer and winter, and standard deviation of summer and winter temperature.

[a] The negative values of non-wildfire $PM_{2.5}$ resulting from the subtraction were treated as zero.

Reference:

1. Childs ML, Li J, Wen J, et al. Daily Local-Level Estimates of Ambient Wildfire Smoke PM(2.5) for the Contiguous US. Environmental science & technology. 2022;56(19):13607-13621.

2. Di Q, Amini H, Shi L, et al. An ensemble-based model of PM(2.5) concentration across the contiguous United States with high spatiotemporal resolution. Environment international. 2019;130:104909.

(9) The calculation of non-wildfire $PM_{2.5}$ by subtracting wildfire-specific $PM_{2.5}$ (Childs et al.) from total $PM_{2.5}$ (Di et al.) may introduce substantial measurement error because the two input products differ in data sources, modeling approaches, spatial resolution, and uncertainty structures. Please discuss the impact on the estimated associations and their comparison.

Response: Thank you for raising this point. We agree that estimating non-wildfire

$PM_{2.5}$ by subtracting wildfire-specific $PM_{2.5}$ (Childs et al. 2022) from total $PM_{2.5}$ (Di et al. 2019) may introduce additional exposure measurement error, given the different modeling approaches used. Because this error is unlikely to be systematically related to health outcomes, it is expected to be largely non-differential and would tend to attenuate the estimated associations for non-wildfire $PM_{2.5}$. With respect to the comparison between wildfire and non-wildfire $PM_{2.5}$, this implies that any observed stronger associations for wildfire $PM_{2.5}$ are likely conservative rather than overstated. Furthermore, to assess the impact of subtraction-related measurement error, we conducted sensitivity analyses in which negative non-wildfire $PM_{2.5}$ values resulting from the subtraction were set to zero. Across the alternative specification, estimated effect sizes changed very little and overall conclusions remained unchanged (Table R5; Supplementary Table 8). These results suggest that our findings are robust to how subtraction-related measurement error is handled and are unlikely to be driven by artifacts of the exposure construction.

Revised text in the Discussion:

Page 17, Line 311-313: Second, estimating non-wildfire $PM_{2.5}$ by subtracting wildfire-specific $PM_{2.5}$ from total $PM_{2.5}$ may introduce non-differential measurement error due to differences in modeling approaches; however, sensitivity analyses treating negative values as zero yielded nearly identical effect sizes (Supplementary Table S8), indicating that our findings are robust to this error.

Reference:

1. Childs ML, Li J, Wen J, et al. Daily Local-Level Estimates of Ambient Wildfire Smoke PM(2.5) for the Contiguous US. Environmental science & technology. 2022;56(19):13607-13621.

2. Di Q, Amini H, Shi L, et al. An ensemble-based model of PM(2.5) concentration across the contiguous United States with high spatiotemporal resolution. Environment international. 2019;130:104909.

(10) The study used ZIP code-level covariates only from the Decennial Census (2000, 2010, 2020), with all intermediate years imputed by linear interpolation. This may introduce substantial covariate misclassification. Please consider using the American Community Survey dataset, which provides annually updated ZIP code-level demographic and socioeconomic data for most of the study period.

Response: Thank you for this comment. We apologize for the lack of clarity in the original description. In fact, our study used a combination of the Decennial Census and the American Community Survey (ACS), rather than relying solely on the Decennial Census.

Specifically, the percentage of White and percentage of Black were derived from the Decennial Census (2000, 2010, and 2020), with values for intermediate years estimated using linear interpolation. Percentage of the population aged ≥65 years who graduated high school, median household income, and the Townsend deprivation index were derived from the 2000 Decennial Census and the 2011 ACS, with intermediate years interpolated, and from 2012 to 2019 were based on ACS estimates. We chose this combined approach because the Decennial Census provides full population coverage and more stable estimates, whereas ACS data are sample-based. Using both sources allowed us to balance representativeness with temporal resolution across the study period. We have revised the Methods section to clarify the sources and construction of these covariates.

Revised text in the Methods:

Page 21, Line 401-411: We extracted zip code-level characteristics from databases generated by US Decennial Census and American Community Survey (ACS).[29-32] Percentage of White and percentage of Black were obtained from the Decennial Census (2000, 2010, and 2020), with values for intermediate years estimated using linear interpolation. Percentage of the population aged ≥65 years who graduated high school, median household income, and Townsend Deprivation Index (TDI) were derived from the 2000 Decennial Census and the 2011 ACS, interpolated for intermediate years, and ACS estimates were used for 2012-2019.

删除[Min Zhang]: neighborhood

删除[Min Zhang]: for 2000, 2010, and 2020

删除[Min Zhang]: For the remaining years, values were estimated using linear interpolation. The following zip code-level variables were included in this study: percentage of White, percentage of Black, percentage of the population aged ≥65 years who graduated high school, median household income, and Townsend Deprivation Index (TDI).

删除[Min Zhang [2]]: obtained from annually updated ACS estimates for 2012-2019

(11) Please clarify whether individuals can contribute more than one hospitalization event. If multiple events per person are included, the analysis should account for within-person correlation, for example, using robust standard errors.

Response: Thank you for this comment. The SID does not contain unique patient identifiers; therefore, we were unable to determine whether multiple hospitalizations were contributed by the same individual and could not directly account for within-person correlation (e.g., using clustered robust standard errors). If such within-person correlation existed, standard errors may be underestimated, potentially leading to overly narrow confidence intervals, although point estimates would not be expected to change materially. Importantly, to reduce the likelihood of false-positive findings due to multiple testing, we applied Bonferroni-corrected 95% confidence intervals, which provide a conservative inference.

Revised text in the Methods and Discussion:

Page 17, Line 319-322: Fourth, the SID lacks unique patient identifiers, and therefore repeated hospitalizations by the same individuals could not be identified. We were not be able to account for potential within-person correlation, which may have led to underestimated standard errors and overly narrow confidence intervals (Murray et al., 2014).

Page 20, Line 397-398: To address the potential inflation of type I error due to multiple comparisons, we applied Bonferroni-corrected 95% CI. As a conservative approach, this correction widens confidence intervals and reduces the probability of false-positive findings.

删除[Min Zhang [2]]: Considering the increased risk of false positives from multiple testing, we applied a Bonferroni correction to adjust 95% CI.

Reference:

Murray A Mittleman, Elizabeth Mostofsky, Exchangeability in the case-crossover design, International Journal of Epidemiology, Volume 43, Issue 5, October 2014, Pages 1645 – 1655, https://doi.org/10.1093/ije/dyu081.

Minor:

(1) Please report both counts and percentages in Table 1.

Response: Thanks. We have revised Table 1 to include both counts and percentages.

(2) Please provide a table with more detailed distribution of the exposure variables (e.g., means, medians, percentiles, and variability measures). This would help assess the plausibility of the exposure contrasts used in the analysis and the robustness of the findings.

Response: Thanks. A more detailed table of the exposure distributions, including means, medians, selected percentiles, and measures of variability (SD and IQR), has been added in the Supplementary Material (Table R6; Supplementary Table 3).

Revised text in the Results:

Page 9, Line 131-134: The 2-year average non-wildfire $PM_{2.5}$ concentrations were approximately 20-27 times higher than that of wildfire-specific $PM_{2.5}$ (Supplementary Table 3).

删除[Min Zhang]: The highest temperature in summer was 35.53 °C, while the lowest temperature in winter is -19.26 °C.

删除[Min Zhang [2]]:

Table R6. The distribution of 2-year averages of wildfire-specific $PM_{2.5}$ and non-wildfire $PM_{2.5}$ (μg/m^3).

Variables	Mean (SD)	Median (IQR)	Percentiles				
			P5	P25	P75	P95	Min-Max
Ischemic heart disease							
Wildfire-specific $PM_{2.5}$	0.376 (0.336)	0.322 (0.261)	0.085	0.200	0.461	0.721	0.004-7.787
Non-wildfire $PM_{2.5}$	8.292 (2.124)	8.271 (2.568)	4.604	6.998	9.567	11.92	0.290-21.566
Cerebrovascular disease							
Wildfire-specific $PM_{2.5}$	0.383 (0.360)	0.318 (0.266)	0.086	0.196	0.462	0.773	0.004-7.787
Non-wildfire $PM_{2.5}$	8.156 (2.061)	8.154 (2.409)	4.570	6.936	9.345	11.723	0.290-20.687
Heart failure							
Wildfire-specific $PM_{2.5}$	0.386 (0.346)	0.328 (0.264)	0.095	0.205	0.469	0.750	0.004-7.787
Non-wildfire $PM_{2.5}$	8.250 (2.049)	8.242 (2.412)	4.739	7.013	9.425	11.819	0.290-20.195
Arrhythmia							
Wildfire-specific $PM_{2.5}$	0.378 (0.340)	0.322 (0.263)	0.082	0.199	0.462	0.733	0.004-7.787
Non-wildfire $PM_{2.5}$	8.134 (2.084)	8.124 (2.463)	4.509	6.889	9.352	11.754	0.290-20.687
Hypertension							
Wildfire-specific $PM_{2.5}$	0.375 (0.342)	0.315 (0.255)	0.094	0.199	0.454	0.734	0.004-7.787
Non-wildfire $PM_{2.5}$	8.266 (2.059)	8.210 (2.454)	4.782	7.013	9.467	11.854	0.290-19.797

Other cardiovascular diseases							
Wildfire-specific $PM_{2.5}$	0.379 (0.349)	0.318 (0.265)	0.084	0.196	0.461	0.747	0.004-7.787
Non-wildfire $PM_{2.5}$	8.148 (2.055)	8.142 (2.401)	4.576	6.934	9.335	11.710	0.290-20.195
Acute respiratory infections							
Wildfire-specific $PM_{2.5}$	0.371 (0.333)	0.315 (0.266)	0.080	0.194	0.459	0.723	0.004-7.787
Non-wildfire $PM_{2.5}$	8.300 (2.061)	8.259 (2.446)	4.794	7.054	9.500	11.870	0.290-19.414
Pneumonia							
Wildfire-specific $PM_{2.5}$	0.379 (0.351)	0.319 (0.270)	0.084	0.194	0.465	0.759	0.004-7.787
Non-wildfire $PM_{2.5}$	8.162 (2.087)	8.159 (2.454)	4.527	6.922	9.376	11.766	0.290-20.175
COPD							
Wildfire-specific $PM_{2.5}$	0.370 (0.330)	0.319 (0.261)	0.086	0.198	0.459	0.710	0.004-7.787
Non-wildfire $PM_{2.5}$	8.276 (2.124)	8.248 (2.532)	4.612	6.989	9.521	11.947	0.290-20.195
Asthma							
Wildfire-specific $PM_{2.5}$	0.364 (0.297)	0.319 (0.258)	0.084	0.199	0.457	0.682	0.004-7.787
Non-wildfire $PM_{2.5}$	8.527 (2.022)	8.501 (2.399)	5.015	7.325	9.725	11.929	0.290-19.586
Other respiratory diseases							
Wildfire-specific $PM_{2.5}$	0.392 (0.396)	0.317 (0.272)	0.084	0.194	0.465	0.817	0.004-7.787
Non-wildfire $PM_{2.5}$	7.981 (2.044)	7.952 (2.356)	4.478	6.783	9.139	11.614	0.290-19.797

(3) Please describe all sensitivity analyses in Methods, not only in Results.

Response: Thanks. Descriptions of all sensitivity analyses have been added to the Methods section in the revised manuscript (Page 12 Line 204-214).

(4) The associations with CVD outcomes were in general stronger among people under 65 years. What could be the explanations?

Response: Thank you for this comment. These results are generally consistent with previous studies showing stronger associations between air pollution exposure and CVD outcomes among younger populations (Zhang et al., 2026; Alexeeff et al., 2023; Wei et al., 2024; Wong et al., 2015; Zhang et al., 2025; Wang et al., 2025). One possible explanation is survivor bias (or depletion of susceptible individuals), whereby individuals who are more vulnerable to $PM_{2.5}$-related cardiovascular effects may experience events or mortality at younger ages, leaving a relatively healthier population among those aged 65 years and older. We have added corresponding

discussion in the revised manuscript, as described below.

"*Consistent with previous studies (Zhang et al., 2026; Wei et al., 2024; Zhang et al., 2025; Wang et al., 2025), we observed generally stronger associations between $PM_{2.5}$ exposure and cardiovascular hospitalization risk among patients under 65 years of age. This pattern may be partly explained by survivor bias, whereby individuals who are more susceptible to $PM_{2.5}$-related cardiovascular effects may experience events or even mortality earlier in life, resulting in attenuated associations among older adults.*"

Reference:

1. Zhang M, Castro E, Shtein A, et al. Wildfire smoke PM2.5 and mortality rate in the contiguous United States: A causal modeling study. Sci Adv. 2026;12(6):eadw5890. doi:10.1126/sciadv.adw5890.

2. Alexeeff SE, Deosaransingh K, Van Den Eeden S, Schwartz J, Liao NS, Sidney S. Association of Long-term Exposure to Particulate Air Pollution With Cardiovascular Events in California. JAMA Netw Open. 2023;6(2):e230561. Published 2023 Feb 1. doi:10.1001/jamanetworkopen.2023.0561.

3. Wei Y, Feng Y, Danesh Yazdi M, et al. Exposure-response associations between chronic exposure to fine particulate matter and risks of hospital admission for major cardiovascular diseases: population based cohort study. BMJ. 2024;384:e076939. Published 2024 Feb 21. doi:10.1136/bmj-2023-076939.

4. Wong CM, Lai HK, Tsang H, et al. Satellite-Based Estimates of Long-Term Exposure to Fine Particles and Association with Mortality in Elderly Hong Kong Residents. Environ Health Perspect. 2015;123(11):1167-1172. doi:10.1289/ehp.1408264.

5. Zhang, Y., Xu, R., Huang, W. et al. Respiratory risks from wildfire-specific PM2.5 across multiple countries and territories. Nat Sustain 8, 474-484 (2025). https://doi.org/10.1038/s41893-025-01533-9.

6. Wang W, Li L, Zhu Q, et al. Differential Effects of Wildfire Smoke Fine Particulate Matter Exposure on Respiratory Disease Emergency Department Visits in

the Western United States. Am J Respir Crit Care Med. 2025;211(11):2086-2095. doi:10.1164/rccm.202502-0350OC.
7. Yu W, Guo Y, Shi L, Li S. The association between long-term exposure to low-level PM2.5 and mortality in the state of Queensland, Australia: A modelling study with the difference-in-differences approach. PLoS Med. 2020;17(6):e1003141. Published 2020 Jun 18. doi:10.1371/journal.pmed.1003141.

Reviewer #2 (Remarks to the Author):

The manuscript by Zhang et al. analyzed cardiopulmonary hospitalizations across 20 US states from 2006 to 2019, linking each patient ZIP code to 2-year average exposures to wildfire-specific and non-wildfire $PM_{2.5}$. The authors implemented an extended self-controlled case-crossover design, comparing each hospitalization year with the years immediately before and after to estimate long-term effects while inherently controlling for all time-invariant individual confounders. Conditional logistic regression models assessed associations between both $PM_{2.5}$ sources and hospitalization risk, adjusting for neighborhood sociodemographic characteristics, temperature, and calendar year. The study found that long-term exposure to $PM_{2.5}$ from wildfire smoke was more strongly associated with increased hospitalization risks than non-wildfire $PM_{2.5}$ for all major cardiovascular and respiratory diseases, with the largest impacts observed for cerebrovascular disease, ischemic heart disease, and asthma. Both $PM_{2.5}$ sources disproportionately affected Hispanic and other minority groups, residents of metropolitan or socioeconomically disadvantaged communities, and individuals with obesity or diabetes, indicating substantial inequities in vulnerability to particle pollution.

Response: Thank you very much for your careful review of our manuscript. We are grateful for your positive evaluation and have addressed your comments and suggestions, as appropriate, in the revised manuscript. Detailed responses are provided below.

Major Comments

1. The Authors use '2-year averages of wildfire-specific $PM_{2.5}$ concentrations' as their principal measure of long-term exposure when evaluating the cardiopulmonary hospitalization risk. Wildfire events generate increases in $PM_{2.5}$ that may persist from several weeks to several months. When these episodic peaks are averaged over a 2-year period, their intensity and timing are substantially diluted. As a result, the exposure metric may not accurately reflect short-term fluctuations that could

precipitate acute cardiopulmonary events. This raises a central methodological concern: the analysis assumes that hospitalizations stem from chronic, long-term exposure, yet the chosen exposure window used does not permit differentiation between chronic effects and acute responses to wildfire-related $PM_{2.5}$ spikes.

Response: Thank you for this thoughtful comment. Our study was designed to evaluate associations with long-term average exposure to wildfire-specific $PM_{2.5}$, consistent with prior studies of chronic air pollution exposure, including wildfire-specific $PM_{2.5}$ exposure (Hao et al., 2025; Hao et al., 2025; Ma et al., 2024; Casey et al., 2024). While wildfire-specific $PM_{2.5}$ is episodic in nature, the use of multi-year average concentrations is a standard approach for capturing cumulative exposure over time. We acknowledge that this metric does not allow separation of acute and chronic effects, which is a general limitation of long-term exposure studies. We have clarified in the revised manuscript that our findings should be interpreted as reflecting associations with long-term average exposure rather than acute triggering effects of short-term wildfire smoke episodes.

Reference:

1. Li J, Lu X, Liu F, et al. Chronic Effects of High Fine Particulate Matter Exposure on Lung Cancer in China. Am J Respir Crit Care Med. 2020;202(11):1551-1559. doi:10.1164/rccm.202001-0002OC.

2. Zhu M, Han Y, Mou Y, et al. Effect of Long-Term Fine Particulate Matter Exposure on Lung Cancer Incidence and Mortality in Chinese Nonsmokers. Am J Respir Crit Care Med. 2025;211(4):600-609. doi:10.1164/rccm.202408-1661OC.

3. Hao H, Xu K, Zhang D, et al. Long-Term Wildfire Smoke Exposure and Increased Risk of Heart Failure in Older Adults. J Am Coll Cardiol. 2025;85(25):2439-2451. doi:10.1016/j.jacc.2025.04.058.

4. Hao H, Xu K, Zhang D, et al. Long-term exposure to wildfire smoke particulate matter and incident stroke: a US nationwide study. Eur Heart J. Published online January 27, 2026. doi:10.1093/eurheartj/ehaf875.

5. Ma Y, Zang E, Liu Y, et al. Long-term exposure to wildland fire smoke PM2.5 and mortality in the contiguous United States. Proc Natl Acad Sci U S A.

2024;121(40):e2403960121. doi:10.1073/pnas.2403960121.
6. Casey JA, Kioumourtzoglou MA, Padula A, et al. Measuring long-term exposure to wildfire PM2.5 in California: Time-varying inequities in environmental burden. Proc Natl Acad Sci U S A. 2024;121(8):e2306729121. doi:10.1073/pnas.2306729121.

2. In addition, caution is warranted when using ambient $PM_{2.5}$ concentrations as a proxy for individual exposure, as factors such as time spent outdoors, physical activity levels, indoor ventilation efficiency, and other behavioral or environmental variables can substantially influence the actual inhaled and deposited doses associated with cardiopulmonary health effects requiring hospitalization.

Response: Thanks for your important comment. We agree that ambient $PM_{2.5}$ concentrations represent outdoor exposure and may not fully capture individual-level exposure due to variability in time-activity patterns, indoor environments, and other behavioral or environmental factors. We have now clarified this limitation in the revised manuscript.

Revised text in the Discussion:

Page 17, Line 309-313: Fifth, residual confounding may remain due to unmeasured time-varying factors, including individual behaviors, indoor exposures, medication use, and co-pollutants (e.g., nitrogen dioxide and ozone), as well as differences between ambient $PM_{2.5}$ concentrations and actual personal exposure.

Reference:

1. Wei Y, Qiu X, Yazdi MD, et al. The Impact of Exposure Measurement Error on the Estimated Concentration-Response Relationship between Long-Term Exposure to PM2.5 and Mortality. Environ Health Perspect. 2022;130(7):77006. doi:10.1289/EHP10389.

3. In Figure 1, the color scales used for wildfire-specific and non-wildfire $PM_{2.5}$ differ,

which may inadvertently give the impression that wildfire-specific $PM_{2.5}$ is more substantial than it actually is. If applying a unified scale would render panel A uniformly blue, this limitation should be clearly acknowledged in the figure caption to prevent misinterpretation.

Response: Thank you for this helpful comment. We have revised Figure 1 to use a unified color scale for both wildfire-specific and non-wildfire $PM_{2.5}$ to ensure direct comparability and avoid potential misinterpretation, as follows:

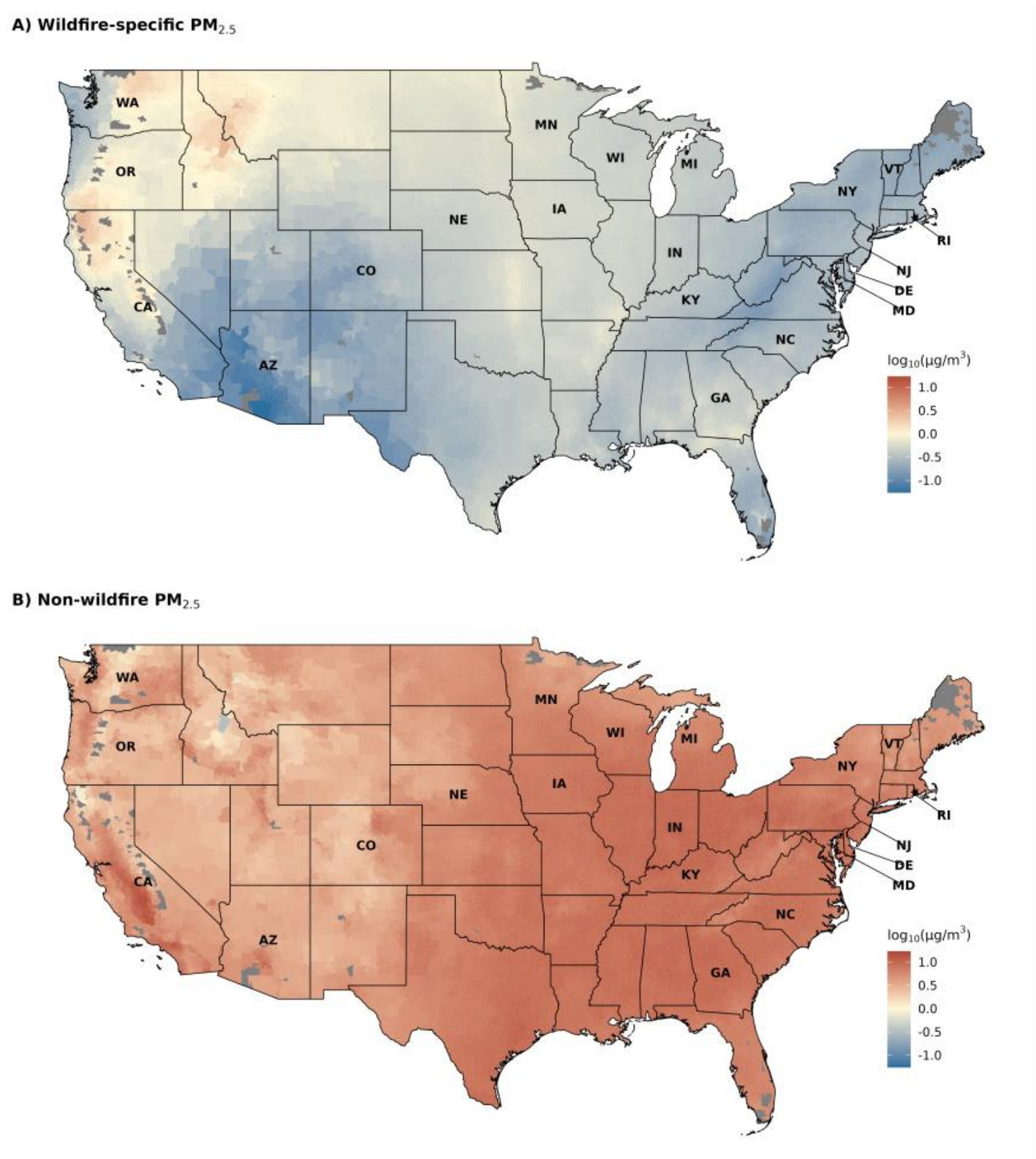

Fig. 1. Average concentrations of wildfire-specific $PM_{2.5}$ and non-wildfire $PM_{2.5}$ ($\mu g/m^3$) in the contiguous United States from 2006-2019. States shown with abbreviations in the inset are included in the study. Values are plotted on a log_{10} scale, with red indicating higher concentrations, blue lower concentrations, and light yellow corresponding to $log_{10} = 0$ (i.e., 1 $\mu g/m^3$).

删除[Min Zhang [2]]: **ure**

删除[Min Zhang [2]]: The states marked with abbreviations in the box are the ones included in our study.

4. Among the 20 states included, California, Oregon, and Washington appear to be the only ones experiencing high-intensity $PM_{2.5}$ exposure from both wildfire-specific and

non-wildfire sources, whereas in the remaining states non-wildfire $PM_{2.5}$ predominates. Have the Authors conducted any region-specific comparisons of the relative cardiopulmonary risks attributable to wildfire versus non-wildfire $PM_{2.5}$ sources? As additional motivation for such an analysis, the vegetative fuels that sustain fires in southern California differ substantially from those in the Pacific Northwest. These differences result in distinct chemical compositions of particulate matter (as well as other gaseous components of smoke not captured in this analysis), which influence smoke toxicity and are likely to modulate the resulting cardiopulmonary health effects.

Response: Thank you for this insightful suggestion. Following this recommendation, we conducted additional state-stratified analyses to compare cardiopulmonary risks associated with wildfire-specific and non-wildfire $PM_{2.5}$ across the 20 states included in our study. We observed that effect estimates were generally stronger in California compared with Oregon and Washington (Table S11). One possible explanation is that California experienced the highest overall $PM_{2.5}$ (both wildfire-specific and non-wildfire $PM_{2.5}$) concentrations during the study period, which may contribute to more obvious risks. In addition, differences in sample size across states may have influenced the precision and magnitude of state-specific effect estimates.

Regarding the role of vegetative fuels, we agree that fuel composition varies across regions and may influence the chemical characteristics and toxicity of wildfire smoke. However, $PM_{2.5}$, as a major toxic component of wildfire smoke, can be transported over thousands of miles, potentially reducing the spatial specificity of source-related compositional differences at the population level. Therefore, while regional variation in fuel type is biologically plausible as a modifier of health effects, our current analysis does not allow us to directly attribute the observed heterogeneity to differences in vegetative fuels. Further research incorporating detailed chemical speciation or source apportionment data would be needed to clarify this issue. We

have added corresponding text to the Discussion section to acknowledge this important consideration.

Revised text in the Results and Discussion:

Page 10-11, Line 160-180 (Results): Overall, the associations between long-term exposure to wildfire-specific $PM_{2.5}$ and non-wildfire $PM_{2.5}$ and risks of cardiopulmonary hospitalizations remained robust after changing covariates adjusted in the model (Supplementary Table 5-6). The effect estimates remained largely unchanged when hospitalizations were identified based solely on the principal diagnosis code at discharge (Supplementary Table 7) or when negative values of non-wildfire $PM_{2.5}$ were modeled as zero instead of being excluded (Supplementary Table 8). When alternative exposure windows were considered using 3- or 4-year moving averages, the association between wildfire-specific $PM_{2.5}$ and cardiopulmonary hospitalizations became stronger with longer exposure duration, whereas the association with non-wildfire $PM_{2.5}$ remained relatively stable (Supplementary Table 9). Furthermore, results were consistent under alternative control-year specifications, indicating that the self-controlled design was not materially sensitive to the selection of control years (Supplementary Table 10). In stratified analyses by US state, associations appeared stronger in states with high $PM_{2.5}$ levels, particularly in California (Supplementary Table 11).

删除[Min Zhang [2]]: These results remained robust after changing the degrees of freedom for temperature up to 9, adjusting for annual precipitation and vapor pressure, or identifying hospitalizations based on the principal diagnosis code at discharge (eTable 3-5 in **Supplement 1**).

Page 18, Line 331-334 (Discussion): Finally, although we distinguished wildfire-specific and non-wildfire $PM_{2.5}$, chemical composition may vary by fuel type

(Mastrolonardo et al., 2017), which we could not assess; future studies incorporating source characterization and chemical speciation are needed to explore potential heterogeneity in health effects.

Reference:

Mastrolonardo G, Hudspith VA, Francioso O, et al. Size fractionation as a tool for separating charcoal of different fuel source and recalcitrance in the wildfire ash layer. Sci Total Environ. 2017;595:461-471. doi:10.1016/j.scitotenv.2017.03.295.

5. In Figure 3, the lower bound of the color bar is unclear. Many of the tiles shaded in red appear to have confidence intervals that include a relative risk of 1. Should these be interpreted as indicating no meaningful change in relative risk rather than a decrease? Clarifying this point would make the figure easier to interpret.

Response: Thank you for this helpful comment. Indeed, tiles shaded in red have confidence intervals that include a relative risk of 1 and therefore should not be interpreted as statistically significant decreases in risk. To improve interpretability of Figure 3, we have revised in the figure caption that the color scale reflects point estimates only, and that estimates with confidence intervals overlapping 1 should be interpreted as indicating no statistically meaningful change in relative risk, as follows:

"***Fig. 3.*** *Heatmap of point estimates of relative risks from subgroup analyses by age, sex, race/ethnicity, and patient location. In the heatmap, colors represent point estimates of relative risk, with white indicating a value of 1 and darker colors indicating point estimates further from 1. Tiles marked with a cross symbol (×) indicate that the corresponding 95% confidence interval overlaps 1 and therefore should not be interpreted as statistically significant changes in relative risk. Tiles without a cross (×) indicate that the corresponding 95% confidence interval does not overlap 1. Age was stratified at 18 years for acute respiratory infections and asthma, and at 65 years for all other disease outcomes. All numerical results, as well as the results of between-group comparisons, can be found in Supplementary Table 13-16.*"

删除[Min Zhang]: ***ure***

删除[Min Zhang]: *Different colors in the heatmap represent different values of relative risks. Values equal to 1 are shown in white, and the deeper the color, the further the value is from 1. Boxes marked with a cross symbol (×) denote that the 95% confidence interval contains 1.*

删除[Min Zhang]: *e*

删除[Min Zhang]: *s*

删除[Min Zhang]: *5*

删除[Min Zhang]: *8*

删除[Min Zhang]: *The Z-test was used when comparing two subgroups, and the Wald test was applied when comparing more than two subgroups.*

6. In Figure 4, the meaning of the shaded areas is unclear. Some of the stratified results are also difficult to interpret. For instance, in the top-left panel, individuals without obesity appear to have a higher risk of hypertension following long-term exposure to wildfire-specific $PM_{2.5}$ than individuals with obesity. Similar patterns are shown for acute respiratory infection and diabetes. These findings run counter to established evidence that obesity and diabetes increase cardiopulmonary risk.

Response: Thank you for this thoughtful comment. The shaded areas in the figure 4 are used solely to visually distinguish wildfire-specific $PM_{2.5}$ and non-wildfire $PM_{2.5}$ and do not carry any statistical meaning. We have revised the figure caption to explicitly clarify this point.

We agree that some stratified patterns in the figure could be misinterpreted if viewed in isolation. Accordingly, we have revised the manuscript to more carefully describe the results. As stated in the revised text, the presence of co-existing obesity or diabetes was generally associated with higher particle-related hospitalization risks for most types of cardiopulmonary diseases. We have refined the language in the revised manuscript to ensure a more cautious and accurate interpretation of these stratified findings.

Revised text in the manuscript:

Figure 4: Figure 4. Scatter plot of point estimates of relative risks from subgroup analyses based on comorbidities, disease severity, and community-level factors. The greater the distance of a point from the diagonal line, the higher the relative risk it represents within a specific subgroup. The shaded areas are used solely for visual differentiation between wildfire-specific $PM_{2.5}$ and non-wildfire $PM_{2.5}$ and do not indicate statistical significance. All relative risks shown have 95% confidence intervals entirely above 1. The percentage of the population who graduated high school was divided into two groups based on the lower quartile (25th percentile): lower and higher educational attainment. Townsend deprivation index was categorized into two groups based on the upper quartile (75th percentile): lower and higher deprivation levels. Detailed numerical results, as well as the results of

between-group comparisons, are provided in Supplementary eTables 9–13. The Z-test was used when comparing two subgroups.

Page 6, Line 79-81 (Abstract): Stronger effects of both wildfire-specific and non-wildfire $PM_{2.5}$ were observed among minorities, those living in metropolitan areas, those with fewer years of education, and more deprived communities.

删除[Min Zhang]: individuals with obesity or diabetes, and

Page 15, Line 265-267 (Results): Moreover, the presence of co-existing obesity or diabetes was generally associated with higher particle-related hospitalization risks for most types of cardiopulmonary diseases (**Figure 4**).

删除[Min Zhang]: increased

Page 19, Line 352-354 (Discussion): Moreover, both wildfire-specific and non-wildfire $PM_{2.5}$ were generally associated with stronger effects for most types of cardiopulmonary diseases among individuals with co-existing obesity or diabetes, highlighting the synergistic effect of $PM_{2.5}$ exposure and cardiopulmonary risk factors.

删除[Min Zhang]: had

Page 19, Line 352-354 (Conclusion): Furthermore, both wildfire-specific and non-wildfire $PM_{2.5}$ had stronger effects among minorities, residents of metropolitan areas, communities with lower lower education attainment, and greater deprivation level.

删除[Min Zhang]: those with co-existing obesity or diabetes, and

Minor Comments

1. The Authors adjusted for unemployment, overcrowded households, non-ownership of a car, and non-ownership of a home as potential confounders. These variables constitute the components of the Townsend Deprivation Index, linked to ZIP code as an indicator of overall material deprivation. However, this index does not incorporate measures of geographic access to services, including routine healthcare beyond the recorded hospital visit, which may also function as a confounding factor.

Response: Thanks. Indeed, the Townsend Deprivation Index (TDI) does not directly incorporate geographic access to healthcare or other services. The TDI was used here as a validated composite measure of overall material deprivation, capturing key socioeconomic dimensions (i.e., unemployment, household overcrowding,

non-ownership of a car, and non-ownership of a home) at the ZIP code level. While the TDI itself may not reflect healthcare accessibility, it serves as a standard surrogate measure of socioeconomic confounding factors in epidemiological studies. We acknowledge that incorporating direct measures of healthcare accessibility could provide additional insights, which could be considered in future work.

Revised text in the Discussion:

Page 19, Line 352-354: Fifth, residual confounding may remain due to unmeasured time-varying factors, including individual behaviors, indoor exposures, medication use, and co-pollutants (e.g., nitrogen dioxide and ozone), as well as differences between ambient $PM_{2.5}$ concentrations and actual personal exposure.

2. The explanation of the self-controlled study design is barebone, making it hard to understand how it works and why it is advantageous.

Response: Thanks. We have expanded the description of the self-controlled study design to improve clarity and help readers better understand how the design works and its advantages. The revised description in the section of Methods is provided below:

Revised text in the Methods:

Page 22-23, Line 426-451: In our previous study (Wei 2025), we extended the traditional case-crossover design, a self-controlled design originally developed for studying short-term transient effects and widely used in environmental epidemiology (Wei 2026; Janes 2005; Consiglio 2013), to evaluate medium-term effects. The underlying principle of the case-crossover framework is a within-individual comparison of exposure levels between a case period and control periods, typically analyzed using conditional logistic regression with each individual forming a matched stratum. If exposure is causally related to the event, exposure levels during the case

period are expected to differ from those during the control periods. Although the traditional case-crossover design focuses on short-term exposures, its validity fundamentally relies on within-individual contrasts under the assumption that time-invariant confounders remain constant across comparison periods. When exposure is defined as multi-year averages, the same logic applies as long as exposure contrasts exist between case and control years.

In this study, we further extended this framework to evaluate long-term effects of 2-year average exposures to wildfire-specific and non-wildfire $PM_{2.5}$ on cardiopulmonary hospitalization risks. Specifically, for each case year (year of hospitalization), the years before and after served as that hospitalization's own controls. With this self-controlled design, unmeasured time-invariant and slowly varying confounders, such as genetics, chronic coexisting conditions (e.g., diabetes), and smoking status, are inherently adjusted by design. The symmetric bidirectional selection of control years before and after a case year removed the unmeasured confounding from long-term temporal trends (e.g., gradual changes in population-level wildfire smoke level). Selecting one single year before and after the case minimized temporal variations in unmeasured confounders.

删除[Min Zhang]: The self-controlled design adjusts for all time-invariant and slowly varying confounders through self-matching, regardless of whether these confounders are measured or not.

删除[Min Zhang]: the traditional case-crossover design, a self-controlled design originally developed for studying short-term effects and widely used,

3. In the Discussion, the finding that individuals residing in metropolitan areas were more susceptible than those in micropolitan or non-CBSA regions is attributed to

higher levels of social stress, which may negatively affect immune responses and increase vulnerability to environmental stressors. However, this explanation is not sufficiently developed. The manuscript does not describe nor cite evidence supporting links between environmental stressors, immune responses, and cardiopulmonary disease, making the rationale incomplete and not fully justified.

Response: Thank you for this important comment. We have expanded the Discussion to better elaborate on the proposed mechanism linking social and environmental stressors, immune dysregulation, and cardiopulmonary outcomes. Specifically, we now describe how chronic psychosocial stress may activate neuroendocrine pathways (e.g., the hypothalamic-pituitary-adrenal axis), promote systemic inflammation, and impair immune responses, thereby increasing susceptibility to environmental exposures. We have also added relevant references to support these links and to provide a more comprehensive rationale for the observed differences between metropolitan and non-metropolitan areas.

In addition, higher background other pollutants (e.g., nitrogen dioxide and ozone) in metropolitan areas (Liu et al., 2021; Simon et al., 2015) may interact with particles, leading to potentially enhanced oxidative stress and greater cardiopulmonary toxicity (Ware et al., 2016; Balmes et al., 2019; Dijkema et al., 2016).

Revised text in the Discussion:

Page 19, Line 348-351: In addition, individuals residing in metropolitan areas (larger urban centers) were more susceptible than those in micropolitan areas or non-Core Based Statistical Areas [non-CBSA], which represent smaller urban clusters and rural areas, respectively. This may be explained by higher background other pollutants (e.g., nitrogen dioxide and ozone) in metropolitan areas (Liu et al., 2021; Simon et al., 2015), which may interact with particles and further enhance oxidative stress and

删除[Min Zhang [2]]: residing

删除[Min Zhang [2]]: non-CBSA

cardiopulmonary toxicity (Ware et al., 2016; Balmes et al., 2019; Dijkema et al., 2016). Additionally, higher levels of social stress in metropolitan areas may activate neuroendocrine stress responses and promote chronic systemic inflammation,[62,63] leading to immune dysregulation and increased susceptibility to cardiopulmonary disease, (Akhmerov 2022; Lee 2016) particularly in the presence of environmental stressors such as particles. (Aslaner et al., 2022).

删除[Min Zhang]: ,

删除[Min Zhang]: which can negatively impact immune responses

删除[Min Zhang]:

删除[Min Zhang]: and thereby increase vulnerability to environmental stressors.

Reference:

1. Liu J, Clark LP, Bechle MJ, et al. Disparities in Air Pollution Exposure in the United States by Race/Ethnicity and Income, 1990-2010. Environ Health Perspect. 2021;129(12):127005. doi:10.1289/EHP8584.

2. Simon H, Reff A, Wells B, Xing J, Frank N. Ozone trends across the United States over a period of decreasing NOx and VOC emissions. Environ Sci Technol. 2015;49(1):186-195. doi:10.1021/es504514z.

3. Ware LB, Zhao Z, Koyama T, et al. Long-Term Ozone Exposure Increases the Risk of Developing the Acute Respiratory Distress Syndrome. Am J Respir Crit Care Med. 2016;193(10):1143-1150. doi:10.1164/rccm.201507-1418OC.

4. Balmes JR. Long-Term Exposure to Ozone and Cardiopulmonary Mortality: Epidemiology Strikes Again. Am J Respir Crit Care Med. 2019;200(8):958-959. doi:10.1164/rccm.201906-1105ED.

5. Dijkema MBA, van Strien RT, van der Zee SC, et al. Spatial variation in nitrogen dioxide concentrations and cardiopulmonary hospital admissions. Environ Res. 2016;151:721-727. doi:10.1016/j.envres.2016.09.008.

6. Aslaner DM, Saldaña TA, MacKenzie DM, et al. Short-term PM exposure and social stress cause pulmonary and cardiac dysfunction. Toxicol Lett. 2022;370:66-73. doi:10.1016/j.toxlet.2022.09.006.

7. Akhmerov A, Parimon T. Extracellular Vesicles, Inflammation, and Cardiovascular Disease. Cells. 2022;11(14):2229. Published 2022 Jul 18. doi:10.3390/cells11142229.

8. Lee KY, Ito K, Maneechotesuwan K. Inflammation to Pulmonary Diseases. Mediators Inflamm. 2016;2016:7401245. doi:10.1155/2016/7401245.

Response to Reviewers

Reviewer #1 (Remarks to the Author):

The authors have done a great job in this revision. I have no further comments. Congratulations!

Response: We sincerely thank the reviewer for the positive comments and encouragement. We are glad that the revision has addressed the concerns satisfactorily.

Reviewer #3 (Remarks to the Author):

The authors have addressed the previous concerns adequately and the revised manuscript is improved. The added justification for the self-controlled design is clearer and the additional sensitivity analyses strengthen the robustness of the findings, particularly the analyses using alternative control-year specifications and exposure windows. The responses are detailed, transparent, and methodologically sound. The study is good and important given the increasing contribution of wildfire smoke to ambient PM2.5 exposure and the large sample size and direct comparison between wildfire-specific and non-wildfire PM2.5 provide evidence regarding differential toxicity and long-term cardiopulmonary risks. I find the response to reviewer #2's concerns convincing overall. I only have a few minor suggestions. Some methodological explanations regarding the self-controlled framework are repeated across the manuscript and could be shortened for clarity. The discussion should also acknowledge more explicitly that overlapping exposure windows would likely attenuate exposure contrasts and bias estimates toward the null rather than inflate associations. In addition, parts of the Discussion comparing prior wildfire studies could be shortened slightly to improve readability. Overall, I believe the manuscript is suitable for publication.

Response: We sincerely thank the reviewer for the thoughtful and positive evaluation of our work. We greatly appreciate the recognition of the methodological

improvements, additional sensitivity analyses, and the overall contribution of the study.

In response to the reviewer's suggestions, we have further streamlined several repeated methodological explanations regarding the self-controlled framework to improve clarity and readability. We have also revised the Discussion to more explicitly acknowledge that overlapping exposure windows would likely attenuate exposure contrasts and bias estimates toward the null. In addition, we slightly shortened parts of the Discussion comparing prior wildfire studies to improve conciseness and readability.

We are grateful for the reviewer's constructive comments and support for publication.

Revised text in the manuscript:

Page 18-19, Line 361-372 (Methods): In our previous study[28], we extended the traditional case-crossover design, a self-controlled design originally developed for studying short-term transient effects and widely used in environmental epidemiology[83-85], to evaluate medium-term effects. The design compares exposure during a case period with exposure during matched control periods within the same individual using conditional logistic regression, thereby inherently controlling for time-invariant confounders. Although traditionally applied to short-term exposures, the same framework can be extended to multi-year average exposures provided that exposure contrasts exist between case and control periods.

Page 9-10, Line 166-174 (Discussion): We observed that a 1-μg/m^3 increase in wildfire-specific $PM_{2.5}$ concentrations was associated with a 10.9%–16.0% increase in pulmonary hospitalization risks, which is generally higher than estimates reported in previous studies (0.25%–2.3%)[21,28].

Page 10, Line 179-185 (Discussion): For the effects of wildfire emissions on cardiovascular diseases, existing evidence remains less conclusive[30-33]. Some studies

删除[Min Zhang]: The underlying principle of the case-crossover framework is a within-individual comparison of exposure levels between a case period and control periods, typically analyzed using conditional logistic regression with each individual forming a matched stratum. If exposure is causally related to the event, exposure levels during the case period are expected to differ from those during the control periods. Although the traditional case-crossover design focuses on short-term exposures, its validity fundamentally relies on within-individual contrasts under the assumption that time-invariant confounders remain constant across comparison periods. When exposure is defined as multi-year averages, the same logic applies as long as exposure contrasts exist between case and control years.

删除[Min Zhang]: a magnitude

删除[Min Zhang]: greater

删除[Min Zhang]: that

删除[Min Zhang]: . A study of eight countries and territories found a 0.25% (RR= 1.0025, 95% CI: 1.0023, 1.0028) increase in all-cause pulmonary hospitalizations per 1-μg/m^3 increase in the 2-day average concentration of wildfire smoke $PM_{2.5}$

删除[Min Zhang]: . Another study of 16 US states reported a 2.1%–2.3% increase in hospitalizations for chronic obstructive pulmonary disease (COPD), asthma, and other respiratory diseases associated with a 1-μg/m^3 increase in 3-month moving average exposure to wildfire smoke $PM_{2.5}$

设置格式[Min Zhang]: 上标

删除[Min Zhang]: Wei et al. found significant associations between 3-month average exposure to wildfire-specific $PM_{2.5}$ and increased hospitalization risks of cerebrovascular disease, hypertension, and other cardiovascular diseases in US

设置格式[Min Zhang]: 字体颜色: 自动设置

have reported positive associations[28,34], whereas others have found little evidence of increased risk during wildfire episodes[35-39] or in association with short-term exposure to wildfire-specific $PM_{2.5}$[26,27,40-42].

Page 14, Line 275-280 (Discussion): Furthermore, the use of 2-year average exposure windows creates overlap between cases and controls, likely attenuating exposure contrasts and biasing effect estimates toward the null rather than inflate associations. Finally, although we distinguished wildfire-specific and non-wildfire $PM_{2.5}$, chemical composition may vary by fuel type[68], which we could not assess; future studies incorporating source characterization and chemical speciation are needed to explore potential heterogeneity in health effects.

设置格式[Min Zhang]: 字体: （默认）Times New Roman, 小四, 字体颜色: 自动设置

设置格式[Min Zhang]: 字体颜色: 自动设置

设置格式[Min Zhang]: 上标

删除[Min Zhang]: . Alexeeff et al. observed that exposure to elevated $PM_{2.5}$ levels during the Mendocino Complex wildfire was associated with increased cardiovascular hospitalization risks, whereas no significant association was observed during the Camp Fire

设置格式[Min Zhang]: 字体颜色: 自动设置

删除[Min Zhang]: .

删除[Min Zhang]: Several studies
