# Supplementary material for: Cardiopulmonary hospitalization risks from wildfire-specific and non-wildfire PM2.5 in 20 US states
Source: Nat Commun. 2026 Sep 17;17:9349. doi: 10.1038/s41467-026-76974-7 (PMC13586224; doi:10.1038/s41467-026-76974-7)
Supplement: Supplementary file 3 [file 41467_2026_76974_MOESM3_ESM.pdf]

nature portfolio

Corresponding author(s): Min Zhang

Last updated by author(s): May 19, 2026

Reporting Summary

Nature Portfolio wishes to improve the reproducibility of the work that we publish. This form provides structure for consistency and transparency in reporting. For further information on Nature Portfolio policies, see our Editorial Policies and the Editorial Policy Checklist.

Statistics

For all statistical analyses, confirm that the following items are present in the figure legend, table legend, main text, or Methods section.

n/a	Confirmed	
☐	☒	The exact sample size (*n*) for each experimental group/condition, given as a discrete number and unit of measurement
☐	☒	A statement on whether measurements were taken from distinct samples or whether the same sample was measured repeatedly
☐	☒	The statistical test(s) used AND whether they are one- or two-sided<br>*Only common tests should be described solely by name; describe more complex techniques in the Methods section.*
☐	☒	A description of all covariates tested
☐	☒	A description of any assumptions or corrections, such as tests of normality and adjustment for multiple comparisons
☐	☒	A full description of the statistical parameters including central tendency (e.g. means) or other basic estimates (e.g. regression coefficient) AND variation (e.g. standard deviation) or associated estimates of uncertainty (e.g. confidence intervals)
☐	☒	For null hypothesis testing, the test statistic (e.g. *F*, *t*, *r*) with confidence intervals, effect sizes, degrees of freedom and *P* value noted<br>*Give P values as exact values whenever suitable.*
☒	☐	For Bayesian analysis, information on the choice of priors and Markov chain Monte Carlo settings
☒	☐	For hierarchical and complex designs, identification of the appropriate level for tests and full reporting of outcomes
☒	☐	Estimates of effect sizes (e.g. Cohen's *d*, Pearson's *r*), indicating how they were calculated

Our web collection on statistics for biologists contains articles on many of the points above.

Software and code

Policy information about availability of computer code

Data collection	No software was used.
Data analysis	All analyses were implemented by R (version 4.5.1) using 'mgcv' R packages. The analysis code is publicly available on GitHub at https://github.com/yycome/Intro_CCS.

For manuscripts utilizing custom algorithms or software that are central to the research but not yet described in published literature, software must be made available to editors and reviewers. We strongly encourage code deposition in a community repository (e.g. GitHub). See the Nature Portfolio guidelines for submitting code & software for further information.

Data

Policy information about availability of data

All manuscripts must include a data availability statement. This statement should provide the following information, where applicable:

- Accession codes, unique identifiers, or web links for publicly available datasets
- A description of any restrictions on data availability
- For clinical datasets or third party data, please ensure that the statement adheres to our policy

The State Inpatient Databases (SID) are available upon request to the Healthcare Cost and Utilization Project (HCUP) upon application and approval, subject to data use agreements (https://hcup-us.ahrq.gov/db/state/siddbdocumentation.jsp). Access requires completion of a data use agreement and institutional approval through HCUP. The local-level wildfire-specific PM2.5 data are publicly available at https://github.com/echolab-stanford/daily-10km-smokePM. The originally predicted, grid level total PM2.5 data are publicly available at https://www.earthdata.nasa.gov/data/catalog/sedac-ciesin-sedac-aqdh-dapm25-us-1km-1.0.

Meteorological data, including temperature, precipitation, and vapor pressure, are publicly available at https://daac.ornl.gov/DAYMET/guides/Daymet_Daily_V4.html. Percentage of White and percentage of Black were obtained from the Decennial Census (2000, 2010, and 2020), with values for intermediate years estimated using linear interpolation. Percentage of the population aged ≥65 years who graduated high school, median household income, and Townsend Deprivation Index (TDI) were derived from the 2000 Decennial Census and the 2011 ACS, interpolated for intermediate years, and ACS estimates were used for 2012-2019.

Research involving human participants, their data, or biological material

Policy information about studies with human participants or human data. See also policy information about sex, gender (identity/presentation), and sexual orientation and race, ethnicity and racism.

Reporting on sex and gender	The term 'sex' has been carefully used in this study. The information of sex has been collected from SID database, and sex-based analyses were performed in the stratified analyses.
Reporting on race, ethnicity, or other socially relevant groupings	Yes, we have complied. The information of ethnicity was collected from SID database, and race/ethnicity-based analyses were performed in the stratified analyses.
Population characteristics	A total of 57,264,966 cardiovascular hospitalizations and 32,691,394 respiratory hospitalizations were included in this study. Covariate-related characteristics, including age, sex, and race/ethnicity, are presented descriptively to characterize the study population. However, given the self-controlled study design, these individual-level characteristics were not included as model covariates, as the design inherently controls for all time-invariant factors through within-individual comparisons. The primary control of confounding is achieved by comparing exposure levels across different time periods within the same individual.<br>Overall, the population was predominantly older adults, with variation in mean age across disease categories (approximately 40–73 years). Sex and race/ethnicity distributions also varied by outcome group, as detailed in Table S1of the manuscript.
Recruitment	The SID contain the universe of the inpatient discharge abstracts in participating States, translated into a uniform format to facilitate multi-State comparisons and analyses.<br>Together, the SID encompass about 97 percent of all U.S. community hospital discharges. Some States include discharges from specialty facilities, such as acute psychiatric hospitals.<br>The SID contain a core set of clinical and nonclinical information on all patients, including individuals covered by Medicare, Medicaid, or private insurance, as well as those who are uninsured.
Ethics oversight	This study was approved by the Institutional Review Board at the Icahn School of Medicine at Mount Sinai.

Note that full information on the approval of the study protocol must also be provided in the manuscript.

Field-specific reporting

Please select the one below that is the best fit for your research. If you are not sure, read the appropriate sections before making your selection.

☐ Life sciences ☐ Behavioural & social sciences ☒ Ecological, evolutionary & environmental sciences

For a reference copy of the document with all sections, see nature.com/documents/nr-reporting-summary-flat.pdf

Ecological, evolutionary & environmental sciences study design

All studies must disclose on these points even when the disclosure is negative.

Study description	This study used a population-based self-controlled case-crossover design to evaluate the long-term associations between wildfire-specific and non-wildfire PM2.5 exposure and cardiopulmonary hospitalization risks.<br><br>The primary treatment factors were long-term (2-year average) exposures to wildfire-specific PM2.5 and non-wildfire PM2.5. The outcome was the occurrence of cardiopulmonary hospitalization. The design does not involve experimental manipulation or factorial structure; instead, it relies on within-individual comparisons across different time periods.<br><br>Each hospitalization (case event) served as the experimental unit, with multiple control years selected both before and after the case year to construct matched comparison periods. This results in repeated observations within individuals over time, effectively forming a self-matched structure that controls for time-invariant confounders. No biological or technical replicates were applicable, as the study is based on observational administrative and environmental data rather than experimental measurements.<br><br>The model further accounts for potential time-varying confounders, including calendar year and seasonal meteorological factors. No interaction terms were pre-specified in the primary analysis. The vulnerable populations were identified. by subgroup analysis.
Research sample	A total of 57,264,966 hospitalizations for cardiovascular diseases and 32,691,394 for pulmonary diseases from 20 US states were included in this study. The study population represents hospitalized patients within these states over the study period, and no organisms, experimental animals, or biological specimens were involved. Therefore, no information regarding organism taxa, sex, age range, or experimental manipulation is applicable.
Sampling strategy	The hospitalization data were obtained from the State Inpatient Databases (SID) covering 20 U.S. states (Arizona, California, Colorado,

	Delaware, Georgia, Iowa, Indiana, Kentucky, Maryland, Michigan, Minnesota, North Carolina, Nebraska, New Jersey, New York, Oregon, Rhode Island, Vermont, Washington, and Wisconsin) during 2006–2019. The study did not involve a sampling procedure; instead, all eligible hospitalization records meeting the inclusion criteria were included. Therefore, the sample size was determined by data availability and study period coverage rather than a priori statistical sample size calculation. Given the large-scale, multi-state administrative dataset, the resulting sample provides sufficient statistical power for the analyses. Cardiopulmonary hospitalizations were identified using the first three discharge diagnosis codes based on ICD-9 or ICD-10.
Data collection	The hospitalization data used in this study were not directly collected by the study team. Instead, they were originally recorded at the hospital level by trained clinical staff and professional medical coders as part of routine clinical care and administrative billing processes.<br>Diagnoses and procedures were documented in hospital discharge records using standardized ICD-9 and ICD-10 coding systems. These records were captured through hospital electronic health record (EHR) and administrative billing systems. The State Inpatient Databases (SID), compiled by the Healthcare Cost and Utilization Project (HCUP), subsequently standardize and aggregate these records for research use.<br>The exposure data (wildfire-specific and non-wildfire PM2.5) were derived from satellite-based and modeled air pollution datasets, as described in the Methods section of the manuscript.
Timing and spatial scale	The hospitalization data were obtained from the State Inpatient Databases (SID) covering the period from January 1, 2006 to December 31, 2019. Data were collected continuously on an annual basis, with no gaps in coverage across the study period. No separate cohorts were defined; instead, all eligible hospitalization records during the study period were included in a population-based design.<br>The spatial scale of the hospitalization data corresponds to patient-level records aggregated across hospitals within 20 U.S. states. Environmental exposure data, including wildfire-specific and non-wildfire PM2.5, were originally available at a fine spatial resolution (grid-level) and were temporally resolved at daily intervals, which were subsequently aggregated to multi-year exposure metrics and linked to hospitalization data at the appropriate spatial units.
Data exclusions	No data were excluded from analyses.
Reproducibility	The analyses in this study were based on reproducible national administrative and environmental datasets, which ensures inherent reproducibility of the findings.<br>To further assess robustness and reproducibility, we conducted extensive sensitivity analyses. These included: (1) varying the degrees of freedom for temperature to 9; (2) additionally adjusting for the mean and standard deviation of summer and winter relative humidity; (3) further adjusting for annual concentrations of nitrogen dioxide and ozone; (4) defining hospitalizations based on the principal diagnosis code only; (5) truncating negative non-wildfire PM2.5 values to zero rather than excluding them; (6) using alternative exposure definitions based on 3-year and 5-year moving averages of PM2.5; (7) using alternative control time windows (two, three, and four years before and after the case year); and (8) conducting state-stratified analyses to assess regional heterogeneity.<br>All sensitivity analyses produced consistent results with the main findings, and no attempts to replicate the analyses yielded conflicting or failed results, confirming the robustness and reproducibility of the study findings.
Randomization	The hospitalization data was allocated into groups by cause of diseases. Therefore, randomization was not applicable.
Blinding	Blinding was not applicable in this study. The analyses were conducted using de-identified, routinely collected administrative hospitalization data and publicly available environmental datasets.<br>There was no experimental group allocation, and the study investigators had no involvement in the original data collection process. All outcomes and exposures were derived from pre-existing, objective records using predefined algorithms (e.g., ICD coding for hospitalizations and modeled PM2.5 exposure estimates). Therefore, investigator blinding during data collection or analysis was not relevant to this study design.

Did the study involve field work? ☐ Yes ☒ No

Reporting for specific materials, systems and methods

We require information from authors about some types of materials, experimental systems and methods used in many studies. Here, indicate whether each material, system or method listed is relevant to your study. If you are not sure if a list item applies to your research, read the appropriate section before selecting a response.

Materials & experimental systems

n/a	Involved in the study
☒	☐ Antibodies
☒	☐ Eukaryotic cell lines
☒	☐ Palaeontology and archaeology
☒	☐ Animals and other organisms
☐	☒ Clinical data
☒	☐ Dual use research of concern
☒	☐ Plants

Methods

n/a	Involved in the study
☒	☐ ChIP-seq
☒	☐ Flow cytometry
☒	☐ MRI-based neuroimaging

Clinical data

Policy information about clinical studies

All manuscripts should comply with the ICMJE guidelines for publication of clinical research and a completed CONSORT checklist must be included with all submissions.

Clinical trial registration	SID are administrative hospital discharge datasets that capture inpatient stays across participating U.S. states.
Study protocol	case-crossover design
Data collection	20 US states, 2006-2019
Outcomes	The hospitalization of cardiovascular (ischemic heart disease, cerebrovascular disease, heart failure, arrhythmia, other cardiovascular diseases) and pulmonary diseases (acute respiratory infections, pneumonia, chronic obstructive pulmonary disease [COPD], asthma, other respiratory diseases)

Plants

Seed stocks	No plants included.
Novel plant genotypes	No plants included.
Authentication	No plants included.
